# Design, synthesis and computational approach of vanillyl–imidazolidinyl–sulfamethoxazole derivatives as potent antimicrobial candidates tackling microbial resistance†

**DOI:** 10.1039/d5md00221d

**Published:** 2025-06-06

**Authors:** Preetesh Kumar Panda, Kakarla Pakeeraiah, Suvadeep Mal, Monalisa Mahapatra, Ajit Kumar Bishoyi, Sudhir Kumar Paidesetty

**Affiliations:** a Medicinal Chemistry Research Laboratory, School of Pharmaceutical Sciences, Siksha ‘O’ Anusandhan (Deemed to be University) Bhubaneswar Odisha 751003 India psudhirkumar@soa.ac.in; b Central Research Laboratory, IMS and SUM Hospital, Siksha ‘O’ Anusandhan (Deemed to be University) Bhubaneswar Odisha 751003 India; c School of Pharmacy and Life Sciences, Centurion University of technology and management Bhubaneswar Odisha 752050 India mona.biotech1989@gmail.com; d Faculty of Pharmacy, C. V. Raman Global University Bhubaneswar Odisha 752054 India

## Abstract

Superbugs are dominating the world due to the misuse and overuse of antibiotics. This study designed and synthesised two sets of compounds, oxazolones (3a–3j) and their respective imidazolones (4a–4j) bearing a sulfonamide functional group, with increased efficacy and capability to tackle microbial resistance. The structural conformation of compounds was determined using different techniques, including ^1^H/^13^C NMR, FT-IR, HRMS and elemental analysis. The binding affinity of the specific targets of these congeners were predicted through molecular docking. The docking results indicated that compounds 4j (−10.36 kcal mol^−1^) and 4g (−8.62 kcal mol^−1^) showed minimum binding energy with strong affinity against target penicillin-binding protein 2a of methicillin resistant *S. aureus* (MRSA) and C14α-demethylase (CYP51) of *C. albicans*, respectively. Furthermore, these compounds were investigated for their antimicrobial efficacies. Compared with gentamicin, the imidazolone-derived compounds 4d and 4g showed significant inhibition in-terms of zone of inhibition and MIC values. However, the oxazolone-derived compound 3i showed a maximum zone of inhibition of 20 mm against a MDR *T. rubrum* strain, which is better than that of ketoconazole. Following these findings, HOMO–LUMO analysis was carried out, and compound 4g showed the smallest energy gap of 3.15 eV. The antibacterial activity of imidazolones is more effective than oxazolones, whereas the action is reversed for fungal strains. To combat against resistant pathogens, multifaced treatments should be followed, and compounds such as 4d and 4g might play a significant role in this regard. The synthetic and biological outcome of the newer vanillyl–imidazolidinyl–sulfamethoxazole derivatives mark a footstep in the drug discovery pipeline in the bacterial resistance era.

## Abstract 2

Potent vanillyl–imidazolidinyl–sulfamethoxazole derivatives were designed and synthesized among which compounds 4d and 4g have shown significant results combating against MDR pathogens.

## Graphical abstract

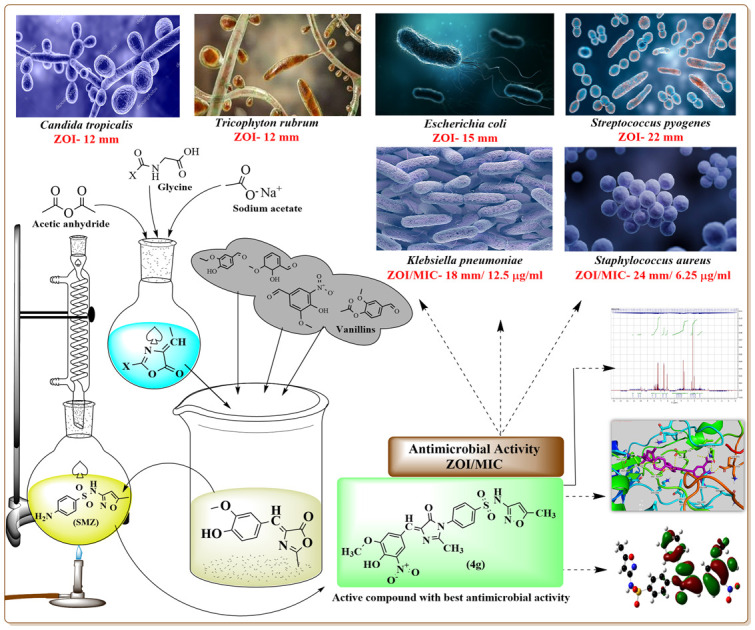

## 1. Introduction

Antimicrobial resistance (AMR) occurs when bacteria become ineffective against antimicrobial agents due to their overuse, resulting in genetic mutations. This adaptation leads to ineffective treatments and exposes significant clinical challenges. Globally, almost 4.95 million annual deaths are associated with AMR, where India contributes a 33% share of around 1.04 million, among which almost 290 000 deaths are solely due to the consequences of AMR as per 2019 reports published by WHO.^1^ A UK review on AMR warns that it could cause 10 million deaths annually by 2050, with a list of bacteria such as *Escherichia coli*, *Staphylococcus aureus*, *Klebsiella pneumoniae*, *Streptococcus pneumoniae*, *Acinetobacter baumannii* and *Pseudomonas aeruginosa* being the most dominant strains likely to contribute to around 660 000–1 270 000 deaths caused by AMR. Overuse of antimicrobials has been a persistent problem contributing to AMR, resulting in much advanced MDR pathogens *viz.* methicillin resistant *S. aureus* (MRSA), carbapenem resistant bacteria, extended spectrum β-lactamase (ESBL), *Mycobacterium tuberculosis* (MDR-TB, XDR-TB), *etc.* Hence, the development of new molecules or the modification of existing molecules has become a prime target for researchers to tackle microbial resistance.^2,3^

**Fig. 1:**
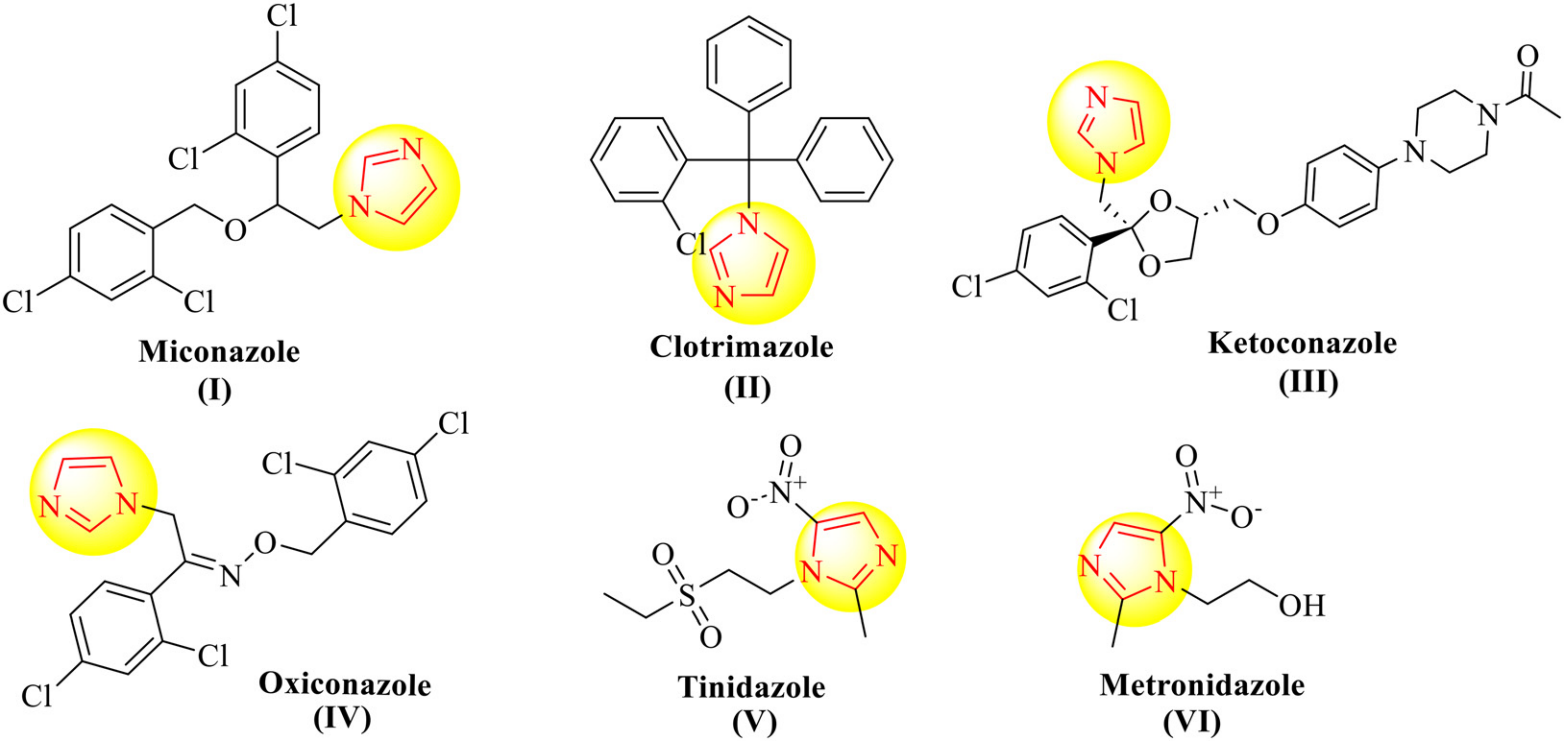
Imidazole containing marketed antimicrobials.

Nitrogen-containing heterocyclic rings such as oxazolones, azlactones or oxazol-5-(4*H*)-ones are unique pharmacophores for treating multiple infectious diseases. Oxazolones are partially saturated five-membered rings containing oxygen and nitrogen atoms with a ketone system. They act as intermediates in the synthesis of different bioactive compounds and are effective against microbial infections, respiratory issues, cardiac problems, and cancer.^4,5^ Replacement of the heteroatom oxygen in oxazole and oxazolones by nitrogen forms imidazole and imidazolones, respectively, which play an integral part in the structure of antifungal drugs such as miconazole, clotrimazole and ketoconazole, which helps inhibit ergosterol synthesis in the cell membrane of fungi (Fig. 1).

Karolina Witek *et al.* developed a series of imidazolone derivatives, among which 1-benzhydrylpiperazine 5-spirofluorenehydantoin substituted imidazolone exhibited remarkable efficiency to break the resistance pattern in a *S. aureus* strain, and it was efficacious as an adjuvant for oxacillin.^6^ A series of 5-arylideneimidazolones were developed and their antimicrobial potency was screened by inhibition of an MDR efflux system through RTE assays with AcrAB-TolC in *Enterobacter aerogenes* (EA289) reported by Banoon *et al.* (2024).^7^ In the series, compound (*Z*)-5-(3,4,5-trimethoxybenzylidene)-3,5-dihydro-4*H*-imidazol-4-one with an imidazolone moiety showed a broad spectrum of antimicrobial activity possibly due to trimethoxybenzylidene attachment and N1 substitution. However, the study has limitations since it was inefficient against resistant pathogens.^7^

Sulfonamides and metal–sulfonamide complexes are gaining more attention due to their antimicrobial potency against bacterial infections harmful to humans. Besides antimicrobials, sulfonamides have also been exploited for their diverse applications as carbonic anhydrase inhibitors, anti-arrhythmic, antifungal, antioxidant, and anti-inflammatory effects.^8–12^ Among sulfanilamides, the drugs sulfamethoxazole (SMZ), silver sulfadiazine, and sulfadoxine are most renowned for their antibacterial efficacy.^13^ Synergistic doses like cotrimoxazole (sulfamethoxazole and trimethoprim), sulfadoxine and pyrimethamine are being used to treat infections caused by both Gram-positive and Gram-negative bacteria alongside the treatment of resistant strains like MRSA (methicillin-resistant *Staphylococcus aureus*).^14^ Therefore, these compounds were incorporated within the imidazolone moieties to enhance the capability of tacking resistant microbial strains. In the supporting literature, Mondal S. *et al.* designed and synthesized some potent Schiff base sulfamethoxazole and sulfathiazole derivatives. Among these candidates, compounds having the 2,4-dichlorosalicylaldehyde ring substituted to sulfamethoxazole through an azomethine linker achieved the lowest MIC value of 16.00 μg mL^−1^ against sulfonamide resistant pathogens including *S. aureus*, *K. pneumoniae* and *E. coli*.^15^

Vanillin, a natural aromatic phenolic compound found in vanilla beans, holds significant importance in addressing microbial resistance by disrupting microbial cell walls and membranes, inhibiting biofilm formation, and interfering with quorum sensing, reducing bacterial communication and virulence.^16,17^ Additionally, vanillin can induce synergism to cephalosporins, gentamicin or imipenem, helping to combat resistant strains of *S. aureus* and *E. coli*. Its adjuvant therapy with norfloxacin increases antibiotic efficacy against *P. aeruginosa*.^18^ Its natural origin, safety, and versatility make it a promising candidate for developing novel antimicrobial strategies.

**Fig. 2:**
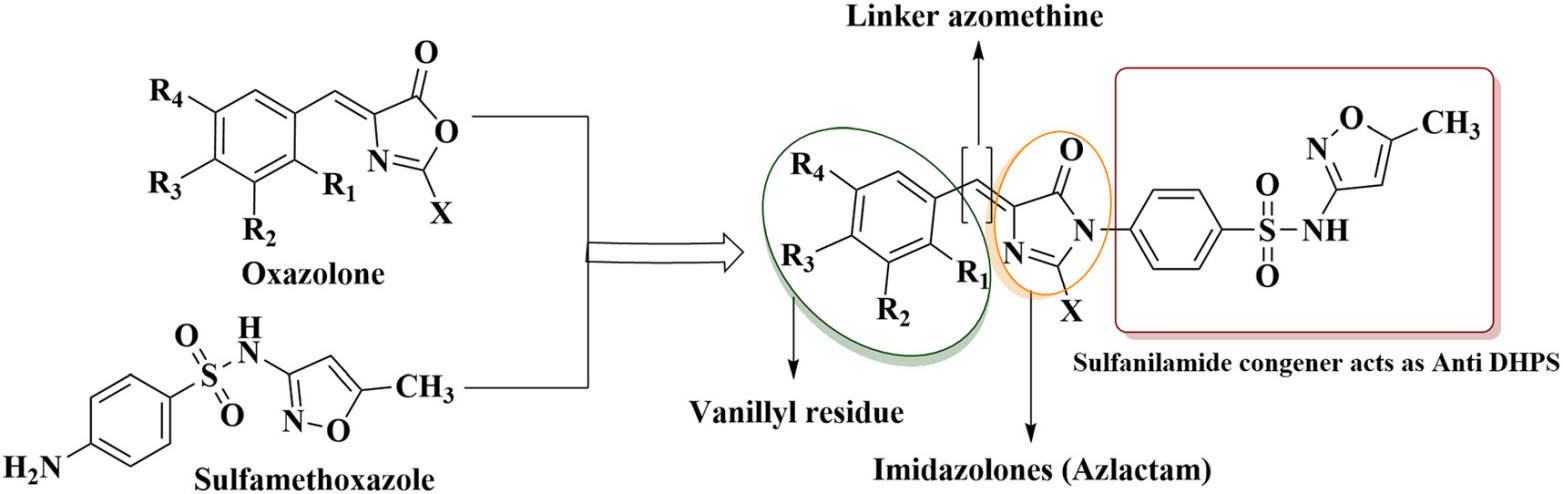
Rational approach behind the study.

The motive of the present study has focused on the advancement of vanillyl attachment to the heterocyclic ring, keeping the valuable roles of imidazolones, sulfonamide or oxazolones in a single structural moiety using molecular hybridization (Fig. 2). In this current research work, azlactones (oxazolones) are screened for antibacterial and antifungal activities and are intermediates for the synthesis of imidazolones. Furthermore, other screened compounds were predicted through computational studies including molecular docking, HOMO–LUMO analysis and ADMET predictions.

## 2. Experimental

### 2.1. Materials

All the chemicals used for this research were of AR grade provided by Sigma-Aldrich and used without purification. Various analytical methods were used to characterize the products and determine their purity. Functional groups were detected by ATR (JASCO FT/IR4600 spectrophotometer), structural environmental status of hydrocarbons was determined by ^1^H/^13^C NMR (Bruker NMR 400 MHz) using TMS as an internal standard, and chemical shifts were reported in terms of ppm, *δ* values. Elemental analysis was carried out using a PerkinElmer 240 analyzer. Similarly, melting points were determined using an Elico apparatus. A thin layer chromatography (TLC) system was used to monitor the progress of the reaction using silica gel 60 F254-coated TLC plates with cyclohexane/ethyl acetate as the solvent system followed by the separation of core compounds from the obtained synthesized mass through column chromatography using different ratios of cyclohexane/ethyl acetate as the solvent.

### 2.2. Methods

#### 2.2.1. Synthesis of vanillyl–oxazolone congeners (3a–3j)

An equimolar concentration (2.23 mmol) of acetyl/benzoyl glycine (1a, 1b), different vanillin derivatives (2a–2e) and sodium acetate were added in a reaction bottle followed by 3 mL of acetic anhydride as the solvent. The reaction was stirred at room temperature for 5–6 h. After completion of the reaction, the homogeneous mixture was poured into ice-cold water and stored overnight in a refrigerator to obtain precipitates (3a–3e) and (3f–3j). The obtained compound was collected after filtration and recrystallized with hot ethanol. The whole reaction process was monitored by TLC using cyclohexane/ethyl acetate as the solvent system.^19^

#### 2.2.2. Synthesis of vanillin–imidazolone congeners (4a–4j)

An equimolar concentration (2.23 mmol) of an ethanolic solution of individual vanillin–oxazolone derivatives (3a–3j) and sulfamethoxazole, along with a few drops of acetic acid were refluxed for 2–3 h at about 80 °C. After completion, the reaction mixture was directly poured into ice-cold water for precipitation and stored overnight in the refrigerator. The obtained precipitate was filtered, collected, and recrystallized from hot ethanol to afford imidazolone congeners (4a–4j). The reaction process was monitored using TLC with cyclohexane/ethyl acetate as the solvent system.^20^

#### 2.2.3. Spectral characterization of oxazolone (3a–3j) and imidazolones (4a–4j)

##### 2.2.3.1. (*Z*)-4-(4-Hydroxy-3-methoxybenzylidene)-2-methyloxazol-5(4*H*)-one (3a)

An equimolar concentration of acetyl glycine (1a), vanillin (2a) and sodium acetate were added in a reaction bottle followed by 3 mL of acetic anhydride as the solvent. Yield: 80–85%; melting point: 104–108 °C; UV-visible (*λ*-max, CH_3_OH): 306 nm; IR (ATR, *γ*, cm^−1^): 3332 (OH str.), 3019 (CH Ar.), 2846 (OCH_3_ str.), 1755 (C=O str.), 1687 (C=N str.), 1647 (CH=C str.), 1597 (C=C str.), 1275 (C–O str.), 795 (CH Ar. bend); ^1^H NMR (400 MHz, DMSO-*d*_6_) *δ* 9.92 (s, 1H, OH), 7.56 (s, 1H, benzylidenyl CH̲), 7.36 (s, 1H, phenylH̲-2), 7.52 (d, 1H, phenylH̲-5), 7.08 (d, 1H, phenylH̲-6), 3.37 (s, 3H, OCH̲_3_), 1.80 (s, 3H, CH̲_3_); ^13^C NMR (400 MHz, DMSO-*d*_6_) *δ* 165.56, 152.11, 144.81, 142.00, 135.59, 131.50, 124.74, 124.28, 124.15, 112.37, 56.52, 20.90; analysis of C_12_H_11_NO_4_ calcd.%: C, 61.86; H, 4.78; N, 6.10; found%: C, 62.66; H, 5.55; N, 7.32; ESI-HRMS (*m*/*z*) anal. Calcd. C_12_H_11_NO_4_ [M + H]: 233.54; found: 234.56 (M + 1).

##### 2.2.3.2. (*Z*)-4-(4-Hydroxy-3-methoxy-5-nitrobenzylidene)-2-methyloxazol-5(4*H*)-one (3b)

An equimolar concentration of acetyl glycine (1a), 5-nitrovanillin (2b) and sodium acetate were added in a reaction bottle followed by 3 mL of acetic anhydride as the solvent. Yield: 80–85%; melting point: 104–108 °C; UV-visible (*λ*-max, CH_3_OH): 306 nm; IR (ATR, *γ*, cm^−1^) 3347 (OH str.), 3089 (CH Ar.), 2847 (OCH_3_ str.), 1771 (C=O str.), 1661 (C=N str.), 1606 (CH=C str.), 1537 (C=C str.), 1263 (C–O str.), 791 (CH Ar. bend); ^1^H NMR (400 MHz, DMSO-*d*_6_) *δ* 9.99 (s, 1H, OH), 8.19 (s, 1H, benzylidenyl-CH̲), 7.25 (s, 1H, phenylH̲-2), 7.93 (s, 1H, phenylH̲-6), 3.64 (s, 3H, OCH̲_3_), 1.81 (s, 3H, CH̲_3_); ^13^C NMR (400 MHz, DMSO-*d*_6_) *δ* 167.88, 155.96, 149.13, 144.48, 134.67, 134.67, 120.94, 119.51, 118.70, 116.84, 55.72, 16.11; analysis of C_12_H_10_N_2_O_6_ calcd.%: C, 51.89; H, 3.68; N, 10.12; found%: C, 52.36; H, 4.19; N, 11.23; ESI-HRMS (*m*/*z*) anal. Calcd. C_12_H_10_N_2_O_6_ [M + H]: 278.26; found: 279.32 (M + 1).

##### 2.2.3.3. (*Z*)-4-(3-Ethoxy-4-hydroxybenzylidene)-2-methyloxazol-5(4*H*)-one (3c)

An equimolar concentration of acetyl glycine (1a), ethylvanillin (2c) and sodium acetate were added in a reaction bottle followed by 3 mL of acetic anhydride as the solvent. Yield: 80–85%; melting point: 104–108 °C; UV-visible (*λ*-max, CH_3_OH): 306 nm; IR (ATR, *γ*, cm^−1^) 3353 (OH str.), 3069 (CH Ar.), 2847 (OCH_3_ str.), 1753 (C=O str.), 1692 (C=N str.), 1598 (CH=C str.), 1581 (C=C str.), 1286 (C–O str.), 781 (CH Ar. bend); ^1^H NMR (400 MHz, DMSO-*d*_6_) *δ* 9.95 (s, 1H, OH), 7.54 (s, 1H, benzylidenyl CH̲), 7.29 (s, 1H, phenylH̲-2), 7.53 (d, 1H, phenylH̲-5), 7.50 (d, 1H, phenylH-6̲), 4.11 (m, 2H, OCH̲_2_CH_3_), 3.34 (s, 3H, OCH_2_CH̲_3_), 1.28 (s, 3H, CH̲_3_); ^13^C NMR (400 MHz, DMSO-*d*_6_) *δ* 168.65, 168.63, 145.09, 136.07, 135.55, 135.54, 124.19, 123.94, 113.66, 113.33, 64.73, 20.44, 14.88; analysis of C_13_H_13_NO_4_ calcd.%: C, 63.20; H, 5.34; N, 5.68; found%: C, 64.55; H, 6.10; N, 6.57; ESI-HRMS (*m*/*z*) anal. Calcd. C_13_H_13_NO_4_ [M + H]: 247.55; found: 248.36 (M + 1).

##### 2.2.3.4. (*Z*)-2-Methoxy-4-((2-methyl-5-oxooxazol-4(5*H*)-ylidene)methyl)phenyl acetate (3d)

An equimolar concentration of acetyl glycine (1a), vanillin acetate (2d) and sodium acetate were added in a reaction bottle followed by 3 mL of acetic anhydride as the solvent. Yield: 80–85%; melting point: 104–108 °C; UV-visible (*λ*-max, CH_3_OH): 306 nm; IR (ATR, *γ*, cm^−1^) 3336 (OH str.), 3016 (CH Ar.), 2843 (OCH_3_ str.), 1760 (C=O str.), 1681 (C=N str.), 1595 (CH=C str.), 1578 (C=C str.), 1272 (C–O str.), 783 (CH Ar. bend); ^1^H NMR (400 MHz, DMSO-*d*_6_) *δ* 7.53 (s, 1H, benzylidenyl CH̲), 7.54 (s, 1H, phenylH̲-6), 7.30 (d, 1H, phenylH̲-2), 7.52 (d, 1H, phenylH̲-3), 3.76 (s, 3H, OCH̲_3_), 2.08 (s, 3H, COCH̲_3_), 1.81 (s, 3H, CH̲_3_); ^13^C NMR (400 MHz, DMSO-*d*_6_) *δ* 135.58, 133.07, 132.50, 129.62, 125.50, 124.27, 124.14, 123.93, 116.41, 112.35, 56.34, 20.90, 15.95; analysis of C_14_H_13_NO_5_ calcd.%: C, 61.14; H, 4.79; N, 5.11; found%: C, 62.14; H, 5.46; N, 6.29; ESI-HRMS (*m*/*z*) anal. Calcd. C_14_H_13_NO_5_ [M + H]: 275.33; found: 276.65 (M + 1).

##### 2.2.3.5. (*Z*)-4-(2-Hydroxy-3-methoxybenzylidene)-2-methyloxazol-5(4*H*)-one (3e)

An equimolar concentration of acetyl glycine (1a), *o*-vanillin (2e) and sodium acetate were added in a reaction bottle followed by 3 mL of acetic anhydride as the solvent. Yield: 80–85%; melting point: 104–108 °C; UV-visible (*λ*-max, CH_3_OH): 306 nm; IR (ATR, *γ*, cm^−1^) 3334 (OH str.), 3000 (CH Ar.), 2843 (OCH_3_ str.), 1763 (C=O str.), 1677 (C=N str.), 1605 (CH=C str.), 1577 (C=C str.), 1273 (C–O str.), 781 (CH Ar. bend); ^1^H NMR (400 MHz, DMSO-*d*_6_) *δ* 10.05 (s, 1H, OH), 8.54 (s, 1H, benzylidenyl CH̲), 7.39 (d, 1H, phenylH̲-6), 7.43 (d, 1H, phenylH̲-4), 7.44 (m, 1H, phenylH̲-5), 3.85 (s, 3H, OCH̲_3_), 2.12 (s, 3H, CH̲_3_); ^13^C NMR (400 MHz, DMSO-*d*_6_) *δ* 157.25, 153.79, 141.12, 129.37, 127.63, 125.45, 121.51, 120.74, 119.15, 112.50, 56.50, 20.63; analysis of C_12_H_11_NO_4_ calcd.%: C, 61.85; H, 4.80; N, 6.11; found%: C, 62.56; H, 5.23; N, 7.31; ESI-HRMS (*m*/*z*) anal. Calcd. C_12_H_11_NO_4_ [M + H]: 233.31; found: 234.66 (M + 1).

##### 2.2.3.6. (*Z*)-4-(4-Hydroxy-3-methoxybenzylidene)-2-phenyloxazol-5(4*H*)-one (3f)

An equimolar concentration of benzoyl glycine (1b), vanillin (2a) and sodium acetate were added in a reaction bottle followed by 3 mL of acetic anhydride as the solvent. Yield: 80–85%; melting point: 104–108 °C; UV-visible (*λ*-max, CH_3_OH): 306 nm; IR (ATR, *γ*, cm^−1^) 3400 (OH str.), 3082 (CH Ar.), 2924 (OCH_3_ str.), 1792, 1752 (C=O str.), 1649 (C=N str.), 1596 (CH=C str.), 1556 (C=C str.), 1262 (C–O str.), 781 (CH Ar. bend); ^1^H NMR (400 MHz, DMSO-*d*_6_) *δ* 10.17 (s, 1H, OH), 8.09 (s, 1H, benzylidenyl CH̲), 7.96 (s, 1H, phenylH̲-2), 7.59 (d, 1H, phenylH̲-5), 7.49 (d, 1H, phenylH̲-6), 7.24 (d, 1H, oxazolonyl phenylH̲-2), 7.32 (d, 1H, oxazolonyl phenylH̲-6), 7.12 (m, 1H, oxazolonyl phenylH̲-3), 7.21 (m, 1H, oxazolonyl phenylH̲-5), 7.09 (m, 1H, oxazolonyl phenylH̲-4), 3.84 (s, 3H, OCH̲_3_); ^13^C NMR (400 MHz, DMSO-*d*_6_) *δ* 161.21, 143.30, 142.72, 140.22, 137.75, 136.76, 132.11, 131.22, 127.17, 124.40, 122.61, 120.74, 115.19, 110.23, 105.88, 102.13, 52.65, 26.28; analysis of C_17_H_13_NO_4_ calcd.%: C, 69.19; H, 4.49; N, 4.76; found%: C, 70.45; H, 5.41; N, 5.14; ESI-HRMS (*m*/*z*) anal. Calcd. C_17_H_13_NO_4_ [M + H]: 295.33; found: 296.17 (M + 1).

##### 2.2.3.7. (*Z*)-4-(4-Hydroxy-3-methoxy-5-nitrobenzylidene)-2-phenyloxazol-5(4*H*)-one (3g)

An equimolar concentration of benzoyl glycine (1b), 5-nitrovanillin (2b) and sodium acetate were added in a reaction bottle followed by 3 mL of acetic anhydride as the solvent. Yield: 80–85%; melting point: 104–108 °C; UV-visible (*λ*-max, CH_3_OH): 306 nm; IR (ATR, *γ*, cm^−1^) 3380 (OH str.), 3096 (CH Ar.), 2938 (OCH_3_ str.), 1764 (C=O str.), 1655 (C=N str.), 1598 (CH=C str.), 1556 (C=C str.), 1289 (C–O str.), 777 (CH Ar. bend); ^1^H NMR (400 MHz, DMSO-*d*_6_) *δ* 9.91 (s, 1H, OH), 7.99 (s, 1H, benzylidenyl CH̲), 7.97 (s, 1H, phenylH̲-2), 7.95 (s, 1H, phenylH̲-6), 7.54 (d, 1H, oxazolonyl phenylH̲-2), 7.53 (d, 1H, oxazolonyl phenylH̲-6), 7.45 (m, 1H, oxazolonyl phenylH̲-3), 7.47 (m, 1H, oxazolonyl phenylH̲-5), 7.31 (m, 1H, oxazolonyl phenylH̲-4), 3.70 (*s*, 3H, OCH̲_3_); ^13^C NMR (400 MHz, DMSO-*d*_6_) *δ* 192.68, 188.65, 129.57, 128.98, 128.73, 127.98, 127.45, 124.21, 123.92, 64.75, 55.69, 55.32, 52.32, 23.20, 20.86; analysis of C_17_H_12_N_2_O_6_ calcd.%: C, 60.47; H, 3.06; N, 8.15; found%: C, 61.26; H, 4.20; N, 9.11; ESI-HRMS (*m*/*z*) anal. Calcd. C_17_H_12_N_2_O_6_ [M + H]: 340.13; found: 341.46 (M + 1).

##### 2.2.3.8. (*Z*)-4-(3-Ethoxy-4-hydroxybenzylidene)-2-phenyloxazol-5(4*H*)-one (3h)

An equimolar concentration of acetyl glycine (1b), ethylvanillin (2c) and sodium acetate were added in a reaction bottle followed by 3 mL of acetic anhydride as the solvent. Yield: 80–85%; melting point: 104–108 °C; UV-visible (*λ*-max, CH_3_OH): 306 nm; IR (ATR, *γ*, cm^−1^) 3405 (OH str.), 3091 (CH Ar.), 2984, 2932 (OCH_3_ str.), 1789, 1761 (C=O str.), 1650 (C=N str.), 1599 (CH=C str.), 1557 (C=C str.), 1267 (C–O str.), 781 (CH Ar. bend); ^1^H NMR (400 MHz, DMSO-*d*_6_) *δ* 10.52 (s, 1H, OH), 7.78 (s, 1H, benzylidenyl CH̲), 8.00 (s, 1H, phenylH̲-2), 7.91 (d, 1H, phenylH̲-5), 7.39 (d, 1H, phenylH̲-6), 7.56 (d, 1H, oxazolonyl phenylH̲-2), 7.50 (d, 1H, oxazolonyl phenylH̲-6), 7.58 (m, 1H, oxazolonyl phenylH̲-3), 7.61 (m, 1H, oxazolonyl phenylH̲-5), 7.34 (m, 1H, oxazolonyl phenylH̲-4), 3.58 (m, 2H, OCH̲_2_CH_3_), 1.13 (s, 3H, OCH_2_CH̲_3_); ^13^C NMR (400 MHz, DMSO-*d*_6_) *δ* 158.14, 154.08, 151.90, 147.65, 141.19, 138.65, 133.10, 130.33, 129.63, 123.50, 119.74, 116.38, 109.45, 107.08, 105.59, 55.98, 17.42, 13.35; analysis of C_18_H_15_NO_4_ calcd.%: C, 69.95; H, 4.92; N, 4.60; found%: C, 70.33; H, 5.20; N, 5.11; ESI-HRMS (*m*/*z*) anal. Calcd. C_18_H_15_NO_4_ [M + H]: 309.55; found: 310.29 (M + 1).

##### 2.2.3.9. (*Z*)-2-Methoxy-4-((5-oxo-2-phenyloxazol-4(5*H*)-ylidene)methyl)phenyl acetate (3i)

An equimolar concentration of acetyl glycine (1b), vanillin acetate (2d) and sodium acetate were added in a reaction bottle followed by 3 mL of acetic anhydride as the solvent. Yield: 80–85%; melting point: 104–108 °C; UV-visible (*λ*-max, CH_3_OH): 306 nm; IR (ATR, *γ*, cm^−1^) 3381 (OH str.), 3089 (CH Ar.), 2847 (OCH_3_ str.), 1792, 1753 (C=O str.), 1649 (C=N str.), 1597 (CH=C str.), 1556 (C=C str.), 1268 (C–O str.), 781 (CH Ar. bend); ^1^H NMR (400 MHz, DMSO-*d*_6_) *δ* 7.97 (s, 1H, benzylidenyl CH̲), 7.47 (s, 1H, phenylH̲-6), 7.50 (d, 1H, phenylH̲-2), 7.55 (d, 1H, phenylH̲-3), 7.44 (d, 1H, oxazolonyl phenylH̲-2), 7.49 (d, 1H, oxazolonyl phenylH̲-6), 7.24 (m, 1H, oxazolonyl phenylH̲-3), 7.27 (m, 1H, oxazolonyl phenylH̲-5), 7.09 (m, 1H, oxazolonyl phenylH̲-4), 3.82 (s, 3H, OCH̲_3_), 2.46 (s, 3H, COCH̲_3_); ^13^C NMR (400 MHz, DMSO-*d*_6_) *δ* 168.96, 166.01, 161.21, 151.90, 151.13, 140.60, 133.60, 133.31, 132.74, 132.58, 129.57, 129.21, 129.08, 128.19, 127.07, 123.61, 123.26, 116.67, 116.38, 114.46, 112.40, 56.55, 23.18, 20.89; analysis of C_19_H_15_NO_5_ calcd.%: C, 67.70; H, 4.53; N, 4.19; found%: C, 69.05; H, 5.56; N, 5.23; ESI-HRMS (*m*/*z*) anal. Calcd. C_19_H_15_NO_5_ [M + H]: 337.38; found: 338.65 (M + 1).

##### 2.2.3.10. (*Z*)-4-(2-Hydroxy-3-methoxybenzylidene)-2-phenyloxazol-5(4*H*)-one (3j)

An equimolar concentration of acetyl glycine (1b), *o*-vanillin (2e) and sodium acetate were added in a reaction bottle followed by 3 mL of acetic anhydride as the solvent. Yield: 80–85%; melting point: 104–108 °C; UV-visible (*λ*-max, CH_3_OH): 306 nm; IR (ATR, *γ*, cm^−1^) 3358 (OH str.), 2938 (CH Ar.), 2839 (OCH_3_ str.), 1763, 1705 (C=O str.), 1653 (C=N str.), 1598 (CH=C str.), 1557 (C=C str.), 1269 (C–O str.), 781 (CH Ar. bend); ^1^H NMR (400 MHz, DMSO-*d*_6_) *δ* 10.05 (s, 1H, OH), 8.52 (s, 1H, benzylidenyl CH̲), 7.43 (d, 1H, phenylH̲-4), 7.33 (d, 1H, phenylH̲-6), 7.46 (m, 1H, phenylH̲-5), 7.91 (d, 1H, oxazolonyl phenylH̲-2), 7.95 (d, 1H, oxazolonyl phenylH̲-6), 7.89 (m, 1H, oxazolonyl phenylH̲-3), 7.84 (m, 1H, oxazolonyl phenylH̲-5), 6.94 (m, 1H, oxazolonyl phenylH̲-4), 3.85 (s, 3H, OCH̲_3_); ^13^C NMR (400 MHz, DMSO-*d*_6_) *δ* 166.45, 160.61, 157.55, 155.66, 149.43, 145.46, 140.52, 136.76, 130.70, 130.13, 129.95, 122.61, 122.22, 119.15, 113.01, 56.38, 20.62; analysis of C_17_H_13_NO_4_ calcd.%: C, 69.19; H, 4.50; N, 4.79; found%: C, 70.05; H, 5.24; N, 5.44; ESI-HRMS (*m*/*z*) anal. Calcd. C_17_H_13_NO_4_ [M + H]: 295.33; found: 296.21 (M + 1).

##### 2.2.3.11. (*Z*)-4-(4-(4-Hydroxy-3-methoxybenzylidene)-2-methyl-5-oxo-4,5-dihydro-1*H*-imidazol-1-yl)-*N*-(5-methylisoxazol-3-yl)benzenesulfonamide (4a)

An equimolar concentration of vanillin–oxazolone derivative (3a) and sulfamethoxazole, along with a few drops of acetic acid were refluxed for 2–3 h at 80 °C to obtain a light yellowish precipitate. Yield: 80–85%; melting point: 104–108 °C; UV-visible (*λ*-max, CH_3_OH): 306 nm; IR (ATR, *γ*, cm^−1^) 3205 (OH/NH str.), 2978 (CH Ar.), 2857 (OCH_3_ str.), 1703 (C=O str.), 1619 (C=N str.), 1592 (CH=C str.), 1497 (C=C str.), 1262 (C–O str.), 1363, 1151 (SO_2_ str.), 1134 (C–N str.), 929 (S–N str.), 781 (CH Ar. bend); ^1^H NMR (400 MHz, DMSO-*d*_6_) *δ* 9.93 (s, 1H, OH), 8.16 (s, 1H, NH), 7.10 (s, 1H, benzylidenyl CH̲), 7.62 (s, 1H, phenylH̲-2), 7.60 (d, 1H, phenylH̲-5), 7.65 (d, 1H, phenylH̲-6), 7.72 (d, 1H, sulfamoyl phenylH̲-2), 7.73 (d, 1H, sulfamoyl phenylH̲-6), 7.87 (d, 1H, sulfamoyl phenylH̲-3), 7.89 (d, 1H, sulfamoyl phenylH̲-5), 6.61 (s, 1H, isoxazolylH̲-4), 3.81 (s, 3H, OCH̲_3_), 2.24 (s, 3H, imidazolidinyl CH̲_3_), 2.24 (s, 3H, isoxazolyl CH̲_3_); ^13^C NMR (400 MHz, DMSO-*d*_6_) *δ* 170.43, 170.34, 168.21, 158.45, 158.39, 154.12, 153.82, 153.82, 138.14, 137.84, 135.70, 132.29, 130.76, 129.61, 123.44, 122.62, 116.10, 114.84, 113.97, 55.91, 23.09, 12.23; analysis of C_22_H_20_N_4_O_6_S calcd.%: C, 56.48; H, 4.38; N, 12.00; S, 6.90; found%: C, 57.45; H, 5.03; N, 12.25; S, 7.54; ESI-HRMS (*m*/*z*) anal. Calcd. C_22_H_20_N_4_O_6_S [M + H]: 468.66; found: 469.33 (M + 1).

##### 2.2.3.12. (*Z*)-4-(4-(4-Hydroxy-3-methoxy-5-nitrobenzylidene)-2-methyl-5-oxo-4,5-dihydro-1*H*-imidazol-1-yl)-*N*-(5-methylisoxazol-3-yl)benzenesulfonamide (4b)

An equimolar concentration of vanillin–oxazolone derivative (3b) and sulfamethoxazole, along with few drops of acetic acid were refluxed for 2–3 h at 80 °C to obtain a light yellowish precipitate. Yield: 80–85%; melting point: 104–108 °C; UV-visible (*λ*-max, CH_3_OH): 306 nm; IR (ATR, *γ*, cm^−1^) 3096 (CH Ar.), 2923, 2852 (OCH_3_ str.), 1698 (C=O str.), 1652 (C=N str.), 1612 (CH=C str.), 1591 (C=C str.), 1260 (C–O str.), 1370, 1159 (SO_2_ str.), 1059 (C–N str.), 1006 (S–N str.), 795 (CH Ar. bend); ^1^H NMR (400 MHz, DMSO-*d*_6_) *δ* 10.96 (s, 1H, OH), 9.86 (s, 1H, NH), 7.15 (s, 1H, benzylidenyl CH̲), 7.66 (s, 1H, phenylH̲-2), 7.70 (s, 1H, phenylH̲-6), 6.52 (s, 1H, isoxazolylH̲-4), 7.73 (d, 1H, sulfamoyl phenylH̲-2), 7.75 (d, 1H, sulfamoyl phenylH̲-6), 7.84 (d, 1H, sulfamoyl phenylH̲-3), 7.81 (d, 1H, sulfamoyl phenylH̲-5), 3.85 (s, 3H, OCH̲_3_), 2.27 (s, 3H, imidazolidinyl CH̲_3_), 2.46 (s, 3H, isoxazolyl CH̲_3_); ^13^C NMR (400 MHz, DMSO-*d*_6_) *δ* 197.99, 194.22, 186.96, 183.42, 174.93, 168.02, 164.40, 160.05, 156.07, 153.82, 144.93, 139.85, 133.43, 129.37, 123.10, 113.08, 106.16, 76.23, 67.64, 20.15, 17.54, 12.57; S, 6.45; found%: C, 52.16; H, 4.25; N, 14.33; S, 7.34; ESI-HRMS (*m*/*z*) anal. Calcd. C_22_H_19_N_5_O_8_S [M + H]: 513.53; found: 514.33 (M + 1).

##### 2.2.3.13. (*Z*)-4-(4-(3-Ethoxy-4-hydroxybenzylidene)-2-methyl-5-oxo-4,5-dihydro-1*H*-imidazol-1-yl)-*N*-(5-methylisoxazol-3-yl)benzenesulfonamide (4c)

An equimolar concentration of vanillin–oxazolone derivative (3c) and sulfamethoxazole, along with few drops of acetic acid were refluxed for 2–3 h at 80 °C to obtain a light yellowish precipitate. Yield: 80–85%; melting point: 104–108 °C; UV-visible (*λ*-max, CH_3_OH): 306 nm; IR (ATR, *γ*, cm^−1^) 3213 (OH/NH str.), 2981 (CH Ar.), 2853 (OCH_3_ str.), 1763 (C=O str.), 1673 (C=N str.), 1612 (CH=C str.), 1592 (C=C str.), 1264 (C–O str.), 1344, 1157 (SO_2_ str.), 1092 (C–N str.), 1036 (S–N str.), 789 (CH Ar. bend); ^1^H NMR (400 MHz, DMSO-*d*_6_) *δ* 9.91 (s, 1H, OH), 9.67 (s, 1H, NH), 8.55 (s, 1H, benzylidenyl CH̲), 6.54 (s, 1H, isoxazolylH̲-4), 7.09 (s, 1H, phenylH̲-2), 7.16 (d, 1H, phenylH̲-5), 7.21 (d, 1H, phenylH̲-6), 7.72 (d, 1H, sulfamoyl phenyl-2), 7.74 (d, 1H, sulfamoyl phenylH̲-6), 7.59 (d, 1H, sulfamoyl phenylH̲-3), 7.61 (d, 1H, sulfamoyl phenylH̲-5), 2.29 (s, 3H, isoxazolyl CH̲_3_), 2.03 (s, 3H, imidazolidinyl CH̲_3_), 4.04 (m, 3H, OCH̲_2_CH_3_), 1.37 (s, 3H, OCH_2_CH̲_3_); ^13^C NMR (400 MHz, DMSO-*d*_6_) *δ* 184.20, 184.08, 176.52, 172.76, 168.84, 166.41, 165.03, 157.95, 145.38, 134.65, 131.42, 130.54, 130.29, 107.20, 107.18, 96.27, 86.86, 68.86, 63.62, 62.31, 22.07, 21.84, 21.38; analysis of C_23_H_22_N_4_O_6_S calcd.%: C, 57.31; H, 4.65; N, 11.68; S, 6.70; found%: C, 58.22; H, 5.64; N, 12.55; S, 7.60; ESI-HRMS (*m*/*z*) anal. Calcd. C_23_H_22_N_4_O_6_S [M + H]: 482.55; found: 483.22 (M + 1).

##### 2.2.3.14. (*Z*)-2-Methoxy-4-((2-methyl-1-(4-(*N*-(5-methylisoxazol-3-yl)sulfamoyl)phenyl)-5-oxo-1,5-dihydro-4*H*-imidazol-4-ylidene)methyl)phenyl acetate (4d)

An equimolar concentration of vanillin–oxazolone derivative (3d) and sulfamethoxazole, along with a few drops of acetic acid were refluxed for 2–3 h at 80 °C to obtain a light yellowish precipitate. Yield: 80–85%; melting point: 104–108 °C; UV-visible (*λ*-max, CH_3_OH): 306 nm; IR (ATR, *γ*, cm^−1^) 3342, 3220 (OH/NH str.), 2926 (CH Ar.), 2853 (OCH_3_ str.), 1793, 1764 (C=O str.), 1653 (C=N str.), 1619 (CH=C str.), 1593 (C=C str.), 1268 (C–O str.), 1374, 1155 (SO_2_ str.), 1091 (C–N str.), 1036 (S–N str.), 777 (CH Ar. bend); ^1^H NMR (400 MHz, DMSO-*d*_6_) *δ* 10.37 (s, 1H, NH), 8.40 (s, 1H, benzylidenyl CH̲), 6.55 (s, 1H, isoxazolylH̲-4), 7.80 (s, 1H, phenylH̲-6), 6.92 (d, 1H, phenylH̲-2), 7.82 (d, 1H, phenylH̲-3), 7.14 (d, 1H, sulfamoyl phenylH̲-2), 7.16 (d, 1H, sulfamoyl phenylH̲-6), 7.31 (d, 1H, sulfamoyl phenylH̲-3), 7.30 (d, 1H, sulfamoyl phenylH̲-5), 3.81 (s, 3H, OCH̲_3_), 2.07 (s, 3H, imidazolidinyl CH̲_3_), 2.23 (s, 3H, isoxazolyl CH̲_3_), 2.49 (m, 3H, COCH̲_3_); ^13^C NMR (400 MHz, DMSO-*d*_6_) *δ* 192.63, 169.00, 168.71, 166.98, 158.51, 152.08, 151.08, 144.73, 135.09, 130.70, 129.16, 129.35, 124.62, 119.11, 115.90, 114.21, 113.83, 113.09, 112.38, 95.80, 55.87, 24.62, 22.87, 12.55; analysis of C_23_H_20_N_4_O_7_S calcd.%: C, 55.66; H, 4.10; N, 11.30; S, 6.50; found%: C, 56.60; H, 5.46; N, 12.33; S, 7.55; ESI-HRMS (*m*/*z*) anal. Calcd. C_23_H_20_N_4_O_7_S [M + H]: 496.53; found: 497.22 (M + 1).

##### 2.2.3.15. (*Z*)-4-(4-(2-Hydroxy-3-methoxybenzylidene)-2-methyl-5-oxo-4,5-dihydro-1*H*-imidazol-1-yl)-*N*-(5-methylisoxazol-3-yl)benzenesulfonamide (4e)

An equimolar concentration of vanillin–oxazolone derivative (3e) and sulfamethoxazole, along with a few drops of acetic acid were refluxed for 2–3 h at 80 °C to obtain a light yellowish precipitate. Yield: 80–85%; melting point: 104–108 °C; UV-visible (*λ*-max, CH_3_OH): 306 nm; IR (ATR, *γ*, cm^−1^) 3336 (OH/NH str.), 2997 (CH Ar.), 2840 (OCH_3_ str.), 1712 (C=O str.), 1681 (C=N str.), 1613 (CH=C str.), 1578 (C=C str.), 1256 (C–O str.), 1359, 1160 (SO_2_ str.), 1090 (C–N str.), 1005 (S–N str.), 777 (CH Ar. bend); ^1^H NMR (400 MHz, DMSO-*d*_6_) *δ* 12.41 (s, 1H, OH), 10.92 (s, 1H, NH), 7.97 (d, 1H, sulfamoyl phenylH̲-2), 8.56 (d, 1H, sulfamoyl phenylH̲-6), 7.88 (d, 1H, sulfamoyl phenylH̲-3), 7.86 (d, 1H, sulfamoyl phenylH̲-5), 7.85 (s, 1H, benzylidenyl CH̲), 6.55 (s, 1H, isoxazolylH̲-4), 7.72 (d, 1H, phenylH̲-4), 6.65 (m, 1H, phenylH̲-5), 7.19 (d, 1H, phenylH̲-6), 3.85 (s, 3H, OCH̲_3_), 2.03 (s, 3H, imidazolidinyl CH̲_3_), 2.26 (s, 3H, isoxazolyl CH̲_3_); ^13^C NMR (400 MHz, DMSO-*d*_6_) *δ* 170.41, 169.66, 169.62, 158.47, 157.73, 153.22, 146.77, 144.03, 129.36, 128.57, 125.47, 125.32, 124.57, 120.73, 119.65, 119.17, 113.09, 112.52, 95.97, 56.59, 24.47, 12.57; analysis of C_22_H_20_N_4_O_6_S calcd.%: C, 56.45; H, 4.33; N, 12.00; S, 6.85; found%: C, 57.22; H, 5.11; N, 12.46; S, 7.60; ESI-HRMS (*m*/*z*) anal. Calcd. C_22_H_20_N_4_O_6_S [M + H]: 468.56; found: 469.33 (M + 1).

##### 2.2.3.16. (*Z*)-4-(4-(4-Hydroxy-3-methoxybenzylidene)-5-oxo-2-phenyl-4,5-dihydro-1*H*-imidazol-1-yl)-*N*-(5-methylisoxazol-3-yl)benzenesulfonamide (4f)

An equimolar concentration of vanillin–oxazolone derivative (3f) and sulfamethoxazole, along with a few drops of acetic acid were refluxed for 2–3 h at 80 °C to obtain a light yellowish precipitate. Yield: 80–85%; melting point: 104–108 °C; UV-visible (*λ*-max, CH_3_OH): 306 nm; IR (ATR, *γ*, cm^−1^) 3396, 3205 (OH/NH str.), 2981 (CH Ar.), 2850 (OCH_3_ str.), 1760 (C=O str.), 1717 (C=N str.), 1620 (CH=C str.), 1595 (C=C str.), 1247 (C–O str.), 1366, 1201 (SO_2_ str.), 1092 (C–N str.), 1010 (S–N str.), 784 (CH Ar. bend); ^1^H NMR (400 MHz, DMSO-*d*_6_) *δ* 10.11 (s, 1H, OH), 10.57 (s, 1H, NH), 7.89 (s, 1H, benzylidenyl CH̲), 6.53 (s, 1H, isoxazolylH̲-4), 7.04 (s, 1H, phenylH̲-2), 7.13 (d, 1H, phenylH̲-5), 7.08 (d, 1H, phenylH̲-6), 7.97 (d, 1H, sulfamoyl phenyl-2), 7.95 (d, 1H, sulfamoyl phenylH̲-6), 7.87 (d, 1H, sulfamoyl phenylH̲-3), 7.91 (d, 1H, sulfamoyl phenylH̲-5), 7.51 (d, 1H, imidazolidinyl phenylH̲-2), 7.47 (d, 1H, imidazolidinyl phenylH̲-6), 7.27 (m, 1H, imidazolidinyl phenylH̲-3), 7.22 (m, 1H, imidazolidinyl phenylH̲-5), 7.49 (m, 1H, imidazolidinyl phenylH̲-4), 3.82 (s, 3H, OCH̲_3_), 2.26 (s, 3H, isoxazolyl CH̲_3_); ^13^C NMR (400 MHz, DMSO-*d*_6_) *δ* 170.43, 170.40, 168.94, 166.64, 165.99, 163.68, 160.68, 158.49, 153.83, 153.79, 151.49, 151.15, 133.61, 129.95, 129.36, 129.09, 128.55, 128.18, 127.06, 124.59, 124.02, 123.63, 114.50, 113.09, 95.81, 56.37, 12.56; analysis of C_27_H_22_N_4_O_6_S calcd.%: C, 61.20; H, 4.25; N, 10.60; S, 6.10; found%: C, 62.19; H, 5.20; N, 11.50; S, 7.40; ESI-HRMS (*m*/*z*) anal. Calcd. C_27_H_22_N_4_O_6_S [M + H]: 530.60; found: 532.00 (M + 1).

##### 2.2.3.17. (*Z*)-4-(4-(4-Hydroxy-3-methoxy-5-nitrobenzylidene)-5-oxo-2-phenyl-4,5-dihydro-1*H*-imidazol-1-yl)-*N*-(5-methylisoxazol-3-yl)benzenesulfonamide (4g)

An equimolar concentration of vanillin–oxazolone derivative (3g) and sulfamethoxazole, along with few drops of acetic acid were refluxed for 2–3 h at 80 °C to obtain a light yellowish precipitate. Yield: 80–85%; melting point: 104–108 °C; UV-visible (*λ*-max, CH_3_OH): 306 nm; IR (ATR, *γ*, cm^−1^) 3355, 3222 (OH/NH str.), 2927 (CH Ar.), 2853 (OCH_3_ str.), 1625 (C=O str.), 1615 (C=N str.), 1592 (CH=C str.), 1539 (C=C str.), 1247 (C–O str.), 1375, 1152 (SO_2_ str.), 1037 (C–N str.), 1007 (S–N str.), 782 (CH Ar. bend); ^1^H NMR (400 MHz, DMSO-*d*_6_) *δ* 10.37 (s, 1H, OH), 9.95 (s, 1H, NH), 7.90 (s, 1H, benzylidenyl CH̲), 6.53 (s, 1H, isoxazolylH̲-4), 7.09 (s, 1H, phenylH̲-2), 7.37 (s, 1H, phenylH̲-6), 7.98 (d, 1H, sulfamoyl phenylH̲-2), 7.96 (d, 1H, sulfamoyl phenylH̲-6), 7.76 (d, 1H, sulfamoyl phenylH̲-3), 7.83 (d, 1H, sulfamoyl phenylH̲-5), 7.56 (d, 1H, imidazolidinyl phenylH̲-2), 7.58 (d, 1H, imidazolidinyl phenylH̲-6), 7.28 (m, 1H, imidazolidinyl phenylH̲-3), 7.35 (m, 1H, imidazolidinyl phenylH̲-5), 7.49 (m, 1H, imidazolidinyl phenylH̲-4), 3.83 (s, 3H, OCH̲_3_), 2.24 (s, 3H, isoxazolyl CH̲_3_); ^13^C NMR (400 MHz, DMSO-*d*_6_) *δ* 170.41, 166.47, 166.22, 158.47, 153.81, 134.70, 133.98, 132.39, 129.36, 129.11, 129.00, 128.94, 128.53, 128.09, 124.57, 121.89, 119.16, 113.08, 95.92, 95.80, 56.10, 52.58, 12.57; analysis of C_27_H_21_N_5_O_8_S calcd.%: C, 56.41; H, 3.72; N, 12.23; S, 5.66; found%: C, 57.40; H, 4.72; N, 13.07; S, 6.17; ESI-HRMS (*m*/*z*) anal. Calcd. C_27_H_21_N_5_O_8_S [M + H]: 575.62; found: 576.11 (M + 1).

##### 2.2.3.18. (*Z*)-4-(4-(3-Ethoxy-4-hydroxybenzylidene)-5-oxo-2-phenyl-4,5-dihydro-1*H*-imidazol-1-yl)-*N*-(5-methylisoxazol-3-yl)benzenesulfonamide (4h)

An equimolar concentration of vanillin–oxazolone derivative (3h) and sulfamethoxazole, along with a few drops of acetic acid were refluxed for 2–3 h at 80 °C to obtain a light yellowish precipitate. Yield: 80–85%; melting point: 104–108 °C; UV-visible (*λ*-max, CH_3_OH): 306 nm; IR (ATR, *γ*, cm^−1^) 3240 (OH/NH str.), 3066 (CH Ar.), 2857 (OCH_3_ str.), 1720 (C=O str.), 1660 (C=N str.), 1640 (CH=C str.), 1594 (C=C str.), 1246 (C–O str.), 1372, 1156 (SO_2_ str.), 1090 (C–N str.), 1039 (S–N str.), 793 (CH Ar. bend); ^1^H NMR (400 MHz, DMSO-*d*_6_) *δ* 10.91 (s, 1H, OH), 10.57 (s, 1H, NH), 7.96 (s, 1H, benzylidenyl–CH̲), 6.53 (s, 1H, isoxazolylH̲-4), 7.37 (s, 1H, phenylH̲-2), 7.08 (d, 1H, phenylH̲-5), 6.06 (d, 1H, phenylH̲-6), 7.73 (d, 1H, sulfamoyl phenylH̲-2), 7.75 (d, 1H, sulfamoyl phenylH̲-6), 7.97 (d, 1H, sulfamoyl phenylH̲-3), 8.0 (d, 1H, sulfamoyl phenylH̲-5), 7.55 (d, 1H, imidazolidinyl phenylH̲-2), 7.57 (d, 1H, imidazolidinyl phenylH̲-6), 7.23 (m, 1H, imidazolidinyl phenylH̲-3), 7.19 (m, 1H, imidazolidinyl phenylH̲-5), 7.47 (m, 1H, imidazolidinyl phenylH̲-4), 4.15 (m, 2H, OCH̲_2_CH_3_), 3.33 (s, 3H, OCH_2_CH̲_3_), 2.24 (s, 3H, isoxazolyl CH̲_3_); ^13^C NMR (400 MHz, DMSO-*d*_6_) *δ* 170.42, 168.90, 166.60, 165.98, 158.47, 153.82, 150.33, 141.00, 133.58, 133.35, 132.64, 132.58, 129.36, 129.06, 128.92, 128.18, 126.95, 124.57, 123.58, 123.34, 115.35, 113.09, 95.80, 64.27, 52.85, 20.84, 14.84, 12.56; analysis of C_28_H_24_N_4_O_6_S calcd.%: C, 61.80; H, 4.53; N, 10.36; S, 5.96; found%: C, 62.71; H, 5.36; N, 11.01; S, 6.64; ESI-HRMS (*m*/*z*) anal. Calcd. C_28_H_24_N_4_O_6_S [M + H]: 544.61; found: 546.01 (M + 1).

##### 2.2.3.19. (*Z*)-2-Methoxy-4-((1-(4-(*N*-(5-methylisoxazol-3-yl)sulfamoyl)phenyl)-5-oxo-2-phenyl-1,5-dihydro-4*H*-imidazol-4-ylidene)methyl)phenyl acetate (4i)

An equimolar concentration of vanillin–oxazolone derivative (3i) and sulfamethoxazole, along with a few drops of acetic acid were refluxed for 2–3 h at 80 °C to obtain a light yellowish precipitate. Yield: 80–85%; melting point: 104–108 °C; UV-visible (*λ*-max, CH_3_OH): 306 nm; IR (ATR, *γ*, cm^−1^) 3362 (OH/NH str.), 2924 (CH Ar.), 2853 (OCH_3_ str.), 1697 (C=O str.), 1609 (C=N str.), 1593 (CH=C str.), 1506 (C=C str.), 1250 (C–O str.), 1373, 1157 (SO_2_ str.), 1091 (C–N str.), 1029 (S–N str.), 789 (CH Ar. bend); ^1^H NMR (400 MHz, DMSO-*d*_6_) *δ* 10.33 (s, 1H, OH), 9.97 (s, 1H, NH), 7.90 (s, 1H, benzylidenyl CH̲), 6.47 (s, 1H, isoxazolylH̲-4), 7.31 (s, 1H, phenylH̲-6), 7.89 (d, 1H, phenylH̲-2), 7.56 (d, 1H, phenylH̲-3), 7.99 (d, 1H, sulfamoyl phenylH̲-2), 8.03 (d, 1H, sulfamoyl phenylH̲-6), 7.87 (d, 1H, sulfamoyl phenylH̲-3), 7.90 (d, 1H, sulfamoyl phenylH̲-5), 7.49 (d, 1H, imidazolidinyl phenylH̲-2), 7.44 (d, 1H, imidazolidinyl phenylH̲-6), 7.42 (m, 1H, imidazolidinyl phenylH̲-3), 7.37 (m, 1H, imidazolidinyl phenylH̲-5), 7.51 (m, 1H, imidazolidinyl phenylH̲-4), 3.81 (s, 3H, OCH̲_3_), 2.20 (s, 3H, isoxazolyl CH̲_3_), 2.24 (s, 3H, COCH̲_3_); ^13^C NMR (400 MHz, DMSO-*d*_6_) *δ* 170.42, 166.42, 166.28, 158.49, 158.48, 153.83, 153.82, 149.13, 149.08, 147.82, 147.81, 133.87, 129.69, 129.37, 129.06, 128.12, 125.19, 124.59, 124.05, 123.67, 113.09, 95.95, 55.71, 14.73, 12.57; analysis of C_28_H_22_N_4_O_7_S calcd.%: C, 60.30; H, 4.01; N, 10.41; S, 5.79; found%: C, 61.26; H, 5.00; N, 11.13; S, 6.41; ESI-HRMS (*m*/*z*) anal. Calcd. C_28_H_22_N_4_O_7_S [M + H]: 558.62; found: 559.30 (M + 1).

##### 2.2.3.20. (*Z*)-4-(4-(2-Hydroxy-3-methoxybenzylidene)-5-oxo-2-phenyl-4,5-dihydro-1*H*-imidazol-1-yl)-*N*-(5-methylisoxazol-3-yl)benzenesulfonamide (4j)

An equimolar concentration of vanillin–oxazolone derivative (3j) and sulfamethoxazole, along with a few drops of acetic acid were refluxed for 2–3 h at 80 °C to obtain a light yellowish precipitate. Yield: 80–85%; melting point: 104–108 °C; UV-visible (*λ*-max, CH_3_OH): 306 nm; IR (ATR, *γ*, cm^−1^) 3361 (OH/NH str.), 2926 (CH Ar.), 2850 (OCH_3_ str.), 1707 (C=O str.), 1660 (C=N str.), 1595 (CH=C str.), 1577 (C=C str.), 1261 (C–O str.), 1364, 1154 (SO_2_ str.), 1113 (C–N str.), 1092 (S–N str.), 766 (CH Ar. bend); ^1^H NMR (400 MHz, DMSO-*d*_6_) *δ* 9.66 (s, 1H, NH), 7.71 (s, 1H, benzylidenyl CH̲), 6.52 (s, 1H, isoxazolylH̲-4), 7.20 (d, 1H, phenylH̲-4), 7.43 (m, 1H, phenylH̲-5), 7.48 (d, 1H, phenylH̲-6), 7.94 (d, 1H, sulfamoyl phenylH̲-2), 7.92 (d, 1H, sulfamoyl phenylH̲-6), 7.60 (d, 1H, sulfamoyl phenylH̲-3), 7.58 (d, 1H, sulfamoyl phenylH̲-5), 7.52 (d, 1H, imidazolidinyl phenylH̲-2), 7.50 (d, 1H, imidazolidinyl phenylH̲-6), 7.41 (m, 1H, imidazolidinyl phenylH̲-3), 7.40 (m, 1H, imidazolidinyl phenylH̲-5), 7.32 (m, 1H, imidazolidinyl phenylH̲-4), 3.88 (s, 3H, OCH̲_3_), 2.24 (s, 3H, oxazolyl CH̲_3_); ^13^C NMR (400 MHz, DMSO-*d*_6_) *δ* 170.41, 166.57, 166.45, 158.48, 158.09, 153.82, 146.87, 133.96, 129.35, 129.20, 128.18, 127.53, 125.61, 124.90, 124.57, 120.48, 119.91, 113.17, 113.08, 95.80, 56.56, 12.61; analysis of C_27_H_22_N_4_O_6_S calcd.%: C, 61.29; H, 4.23; N, 10.58; S, 6.10; found%: C, 62.10; H, 5.01; N, 11.13; S, 7.02; ESI-HRMS (*m*/*z*) anal. Calcd. C_27_H_22_N_4_O_6_S [M + H]: 530.60; found: 532.00 (M + 1).

#### 2.2.4. Molecular docking study

The crystal structures of C14-α demethylase (CYP51) of prominent fungal pathogen *C. albicans* (PDB: **5V5Z**) and penicillin-binding protein-2a from methicillin resistant *Staphylococcus aureus* (MRSA) (PDB: **1VQQ**) were selected as a fungal target and β-lactam resistance for the bacterial target, respectively. Both protein structures were retrieved from RCSB Protein Data Bank (PDB). Further, the newly designed oxazolone (3a–3j) and imidazolone (4a–4j) candidate ligands were drawn and checked for proper bonding interactions using ChemDraw Ultra tool 19.0, which were further optimized using Avogadro 1.1.1 and ACD Labs freeware 2015.^21^ Second, all the ligands and proteins were saved in the (.) pdb format and minimized. The ligands were examined with two distinct antimicrobial enzyme targets using the blind docking approach removing all water molecules and ligands present in the protein structure, which allows us to explore the whole protein surface area besides the native ligand active site. Docking was performed using Autodock 4.2, a software developed by Scripps Research Institute which facilitates precise and accurate docking of binding sites and conformers.^22^ The docking protocol was followed by adding Kollman charges and polar hydrogens merging into the protein structure. During grid box preparation, the spacing was set as 0.375 Å, the coordinates of the central grid points of the maps were set at −45.709, −15.009, and 25.355 for the *x*, *y* and *z* coordinates representing 40 points, which covered the whole structure area for docking. The genetic algorithm (GA) runs were set to 100 ns. for all compounds with a population size of 150 and default parameters were used for the other settings. Lastly, a Lamarkian genetic algorithm was used for computation of the docking studies. All interactions for post-docking operations were deciphered using Pymol and BIOVIA Discovery Studio Visualizer version 4.5 (BIOVIA DSV) to visualize and confirm residue interactions of the docked congeners.^23^

#### 2.2.5. Physicochemical evaluation

The predictive study of physicochemical and pharmacokinetics properties of newly derived compounds (3a–3j and 4a–4j) was done using pre-ADMET, which is a web based online platform. The parameters include the hydrogen donor count (HB), hydrogen acceptor count (HA), total polar surface area (tPSA), octanol/water coefficient (clog *P*), and molecular weight (MW). Similarly, pharmacokinetic parameters like the blood–brain barrier (BBB), Caco-2 permeability (Caco-2 cells used as a model for the oral medication retention technique), human digestive retention (amount of bioavailability and ingestion), skin penetrability and toxicity (LD_50_) were also examined in this software.^24^

#### 2.2.6. Antimicrobial activity

The newly synthesized compounds 3a–3j and 4a–4j were examined in a sterilized environment against six MDR strains to determine their true potential. For this examination, two fungal strains (dermatopathogenic) *viz. Candida tropicalis* (MCC 1559) and *Trichophyton rubrum* (MCC 1598), two Gram-negative bacterial strains (*Klebsiella pneumoniae* (MTCC 1928) and *Escherichia coli* (NCTC 10418)) and two Gram-positive bacterial strains (*Streptococcus pyogenes* (MTCC 1938) and *Staphylococcus aureus* (NCTC 6571)) have been selected. Then, the compounds were evaluated for their prominent antimicrobial properties. Furthermore, a comparative study was done between the new compounds and two marketed drugs (gentamicin and ketoconazole) for their antibacterial and antifungal potency, respectively to strengthen the literature.

##### 2.2.6.1. Agar well diffusion method

The antibacterial and antifungal evaluation of novel compounds was carried out using an agar well diffusion method. Before starting the process, the strains were cultured using Muller–Hinton broth (MHB) and stored overnight in normal room temperature. Then, the culture was spread over sterilized Muller–Hinton agar (MHA) plates. 80 μL of test sample dissolved in DMSO at a concentration of 100 μg mL^−1^ was loaded into each aseptic tube. Both Gram-positive and Gram-negative strains were taken as controls and gentamicin were used as the standard ligand. Similarly, fungal strains were taken as controls and ketoconazole was used as the standard ligand. All the isolated plates were incubated for 24 hours at room temperature (37 °C ± 2 °C). Following incubation, the zone of inhibition (ZOI) was measured in using a millimeter (mm) scale.^25^

##### 2.2.6.2. Minimum inhibitory concentration (MIC)

The minimum inhibitory concentration (MIC) was examined using a 96-well microplate as the concentration that can inhibit fungal and bacterial growth. First, 100 μL of Sabouraud dextrose broth (SDB) was poured for fungus plates and Muller–Hinton broth (MHB) for bacterial plates. The separate serial dilution wells (No. 1–11) were filled with ten aliquots of 1 mg mL^−1^ of CO–AgNPs prepared in sterilized distilled water with the last well prepared as the control. Both the fungal and bacterial cultures were added as 20 μL aliquots, followed by the addition of 5 μL of 5% triphenyl tetrazolium chloride (TTC) in every well. The cultures were incubated at 37 °C for 48 h and 18 h using a BOD incubator for optimal fungal and bacterial growth, respectively.^23^

#### 2.2.7. HOMO–LUMO analysis

The structural parameters derived from the optimized geometries of the synthesized compounds offer critical insight into molecular interactions, serving as an essential preliminary step for subsequent molecular docking analyses. Frontier molecular orbital (FMO) analysis is widely used to explain the electronic properties of organic compounds using quantum chemical calculations. The highest occupied molecular orbital (HOMO)-lowest unoccupied molecular orbital (LUMO) energies and global reactivity parameters of compounds were calculated using DFT at the B3LYP/3-21G level of theory. The Gaussian 09 software package was used in the optimization process and the molecular structures were visualized using GaussView software.^24^

## 3. Results and discussion

### 3.1. Chemistry

**Scheme 1:**
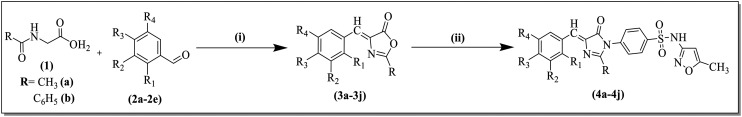
Synthesis of vanillyl–imidazolidinyl–sulfamethoxazole derivatives. a/f = H (R_1_), OCH_3_ (R_2_), OH (R_3_), H (R_4_); b/g = H (R_1_), OCH_3_ (R_2_), OH (R_3_), 5-NO_2_ (R_4_); c/h = H (R_1_), OCH_2_CH_3_ (R_2_), OH (R_3_), H (R_4_); d/i = H (R_1_), H (R_2_), OCOCH_3_ (R_3_), OCH_3_ (R_4_); e/j = OH (R_1_), OCH_3_ (R_2_), H (R_3_), H (R_4_). Reaction condition: (i) NaOAc, Ac_2_O, stirring, 5–6 h; (ii) SMZ, EtOH, CH_3_COOH, reflux, 2–3 h.

Two series of compounds were synthesized using the Erlenmeyer–Plöchl reaction principle: oxazolones (3a–3j) and imidazolones with a sulfonamide moiety (4a–4j). The condensation of acetyl/benzoyl glycine with substituted vanillin (2a–2e) gave rise to an intermolecular cyclization of acetylated amino acids with aromatic aldehydes in the presence of sodium acetate and acetic anhydride as solvent afforded oxazolones (3a–3j). Further reaction of these oxazolones with sulfamethoxazole led to ring-opening because of the nucleophilic attack of aromatic amine of SMZ into the first positioned oxygen atom of oxazolones. Subsequent cyclization forms the final imidazolone products (4a–4j) (Scheme 1). The complete reaction process of all derived compounds was checked using TLC with the appropriate solvent system (cyclohexane : ethyl acetate) and the products were recrystallized with hot ethanol. Physical properties like the color, nature, solubility and melting point of all compounds were checked: most compounds were amorphous, while compounds with nitro substitutions were yellowish in color. All extracted compounds were highly soluble in ethanol, methanol, and DMSO, whereas they were sparingly soluble in nonpolar solvents. The melting point of all the synthesized compounds was between 98–104 °C. The structure of compounds was confirmed using various spectral techniques, *viz.* detection of maximum wavelength by UV-visible spectroscopy, detection of functional groups by FT-IR, determination of the environmental status of hydrocarbons in the structure by NMR, analysis of the molecular weight by HRMS, and detection of the percentage of elements like C, H, N, and S using an elemental analyzer.

The compounds were analyzed using different spectral techniques to gain insights into their structural positioning, including the identification of functional groups and other molecules. In the FT-IR spectrum, carbonyl stretching (C=O str.) of oxazolones appears in the range of 1771–1752 cm^−1^, whereas C–O stretching of the oxazolone derivatives is observed between 1286–1262 cm^−1^, confirming the presence of the cyclic ester bond within the ring. Furthermore, C=N stretching is observed in the range of 1692–1649 cm^−1^, and the exocyclic double bond stretching (C=C Ar.) appears between 1606–1595 cm^−1^, which confirms the presence of an oxazolone ring with a substituted vanillylidene. The vanillyl ring attached to the oxazolone can be identified by the presence of –OH and aromatic –CH stretching bands, which appear between 2930–3360 cm^−1^. In the case of imidazolones, the compound's lactone-carbonyl stretching (C=O str.) was shown in the range of 1712–1697 cm^−1^, which was relatively lower than that of the oxazolone compounds. Furthermore, the sulfonamide group of sulfamethoxazole in the imidazolone structures can be confirmed by the sulfonyl SO_2_ stretching frequencies which appear in the range of 1373–1344 cm^−1^ for asymmetric stretching and 1201–1154 cm^−1^ for symmetric stretching. Additionally, the presence of a methoxy group in the vanillyl residue of all the compounds was confirmed by the O-CH_3_ stretching vibrations, which contributes to the frequencies ranging between 2932–2840 cm^−1^. The frequencies of the obtained compounds were discussed through spectral characterization.

Similarly, ^1^H/^13^C NMR data of newly designed and synthesized hydrocarbon skeletons were analyzed to obtain spectral evidence of their structural environments. Among all compounds, two sharp singlet de-shielded and shielded proton signals were observed in a range of *δ* 3.37–4.15 ppm that evidenced the presence of methoxy (OCH_3_) or ethoxy (OCH_2_CH_3_) groups of the vanillylidene structure. Another singlet appeared in the range of δ 1.81–2.26 ppm corresponding to the methyl (CH_3_) group substituted at the second position of oxazolones (3a–3j) and fifth position of the isoxazole ring of imidazolones (4a–4j), respectively. Compounds with an acetate (OCOCH_3_) group in their structure (3d, 3i, 4d and 4i) have shown a de-shielded protonic signal between *δ* 2.08–2.49 ppm and carbon shifting of these groups were observed within *δ* 55–57 ppm. These chemical shifts from higher to lower values were due to the presence of more electronegative atoms attached to the methyl group. Similarly, the allylic linker between vanillylidene and imidazolone rings of compound 4a–4j showed protonic chemical shifting between *δ* 7.70–7.98 ppm. Meanwhile, the methine proton signals of the linker connecting vanillylidene with the oxazolone rings of compound 3a–3j appeared in the range of *δ* 7.97–8.54 ppm. The same linker carbons can be observed in the carbon NMR peaks ranging between *δ* 110–115 ppm. Among all compounds, multiplet signals were observed for the vanillylidene aromatic shielded protons in the range of *δ* 7.09–7.97 ppm, whereas their carbon shifts were shown between *δ* 100–140 ppm. Also, the carbonyl carbon of lactone and lactam has chemical shifts between *δ* 163.68–168.65 ppm. Few extra supportive peaks were observed for the sulfonamide bearing derivatives (4a–4j), which signifies the presence of an amine group along with sulfamoyl carbons and protons. The amine group (–NH) of sulfamethoxazole reflected a chemical shift between *δ* 8.16–10.92 ppm throughout the compounds. The isoxazole ring present in the SMZ structure has shown a singlet as a chemical shift for the fourth position between *δ* 6.47–6.61 ppm along with double doublets for the aromatic ring containing the nitrogen atom of the imidazolone ring shown between *δ* 7.14–7.98 ppm.^21^ The spectra of the given compounds were depicted in the ESI† file (Fig. S1 to S60). All plausible evidence that support the structural integrity of the newly designed compounds was discussed, and the results confirmed the presence of all integral structural elements in the synthesized compounds.

### 3.2. Computational evaluation of synthesized congeners

#### 3.2.1. Molecular docking study

The docking study was done against two different enzymes retrieved from the Protein Data Bank (PDB), including one for the antifungal study (PDB: **5V5Z**, structure of CYP51 from the pathogen, *C. albicans*) and another one for the bacterial study (PDB: **1VQQ**, structure of penicillin-binding protein 2a from the MRSA strain). The results of the docking study of various oxazolone–imidazolone congeners with the standard drugs gentamicin and ketoconazole is depicted in Table 1. By referencing *in vitro* antibacterial and antifungal activity depicted in Table 4, the two anticipated standard antibacterial and antifungal drugs (gentamicin and ketoconazole, respectively) were used for a comparative study. The selection of **5V5Z** was completely based on lanosterol C14 α-demethylase inhibition efficiency, since azoles are the lead antifungal drugs in the market and one of the targets is **5V5Z**. The inhibition of lanosterol biosynthesis has emerged as a primary focus of research due to its transformation into ergosterol facilitated by cytochrome P-450 within the fungal cell wall given that the target can be effectively inhibited *via* active interaction with the co-crystallized ligands. The protein shows multiple co-factors in its binding region *viz.* TYR118, TYR132, LEU376, and HIS377 that are being considered as the active site binding pocket of the protein.^26^ Similarly, **1VQQ** is the penicillin-binding protein which is derived from the methicillin resistant *Staphylococcus aureus* strain.^27^ Some previous studies show that the sulfonamide congeners have good binding profiles and docking results against this specific target.^23,28^ However, certain calculations were not performed in this study, including p*K*_a_ values of the ligands. Instead, a default protonation factor was assumed for all ligands at a neutral pH of 7.4 during ligand preparations using the Autodock tool.

**Table 1:** Docking energy (kcal mol^−1^) and interactions of the newly synthesized oxazolone (3a–3j) and imidazolone (4a–4j) candidates

Oxazolone–imidazolone compounds	**1VQQ** penicillin binding protein 2a from MRSA	Residue interactions	**5V5Z** CYP51 from *C. albicans*	Residue interactions
3a	−6.45	LYS153, ASP323, ASP552	−6.53	THR315, LEV376
3b	−6.28	LYS153, ASP323, GLN325	−7.34	TYR118, LEU376, ARG381
3c	−6.02	LYS153, ASP323, GLN325, ASP552	−6.82	THR315, LEU376
3d	−7.07	LYS153, ASP323, ASP552	−7.59	THR315, LEU376
3e	−5.78	LYS153, ASP323, GLN325	−6.79	THR315, PRO462
3f	−6.81	ASN159, ASP323, ASP552	−8.17	THR315, LEU376
3g	−6.86	LYS153, ASP323	**−9.55**	**TYR118, LEU376, ARG381**
3h	−7.00	ASP323, GLN325, ASP552	−8.30	THR315, LEU376
3i	**−7.30**	**ASN159, GLN325**	−9.20	THR315, LEU376
3j	−7.19	LYS153, ASP323, GLN325	−8.26	THR315, LEU376
4a	−7.63	ASN159, ASP323, ASP552	−8.90	TYR118, ARG381, GLY464
4b	−7.26	LYS153, GLY321, GLN325, ASP552	−7.73	ARG389
4c	−7.93	LYS153, ASN159, GLU161, ASP323	−10.29	TYR118, ARG381, CYS470, GLY472
4d	−7.91	—	−8.55	ARG381, GLN479
4e	−8.47	GLU161, ASP323	−9.31	ARG381, GLN479
4f	−8.39	ASP323, ASP552	−9.84	TYR118, ARG381
4g	**−8.62**	**ASN159, GLN325**	−9.59	THR311, ARG381, GLN479
4h	−8.07	LYS153, ASN159, ASP552	−9.19	TYR118, ARG381
4i	−7.59	LYS153, ASN159, GLU161	−9.41	—
4j	−8.56	ASP323, GLN325, ASP552	**−10.36**	**TYR118, GLY308, ARG381, CYS470**
Gentamicin/ketoconazole	−10.89	LYS153, GLU161, GLY321, ASP323	−9.13	LEU376

**Fig. 3:**
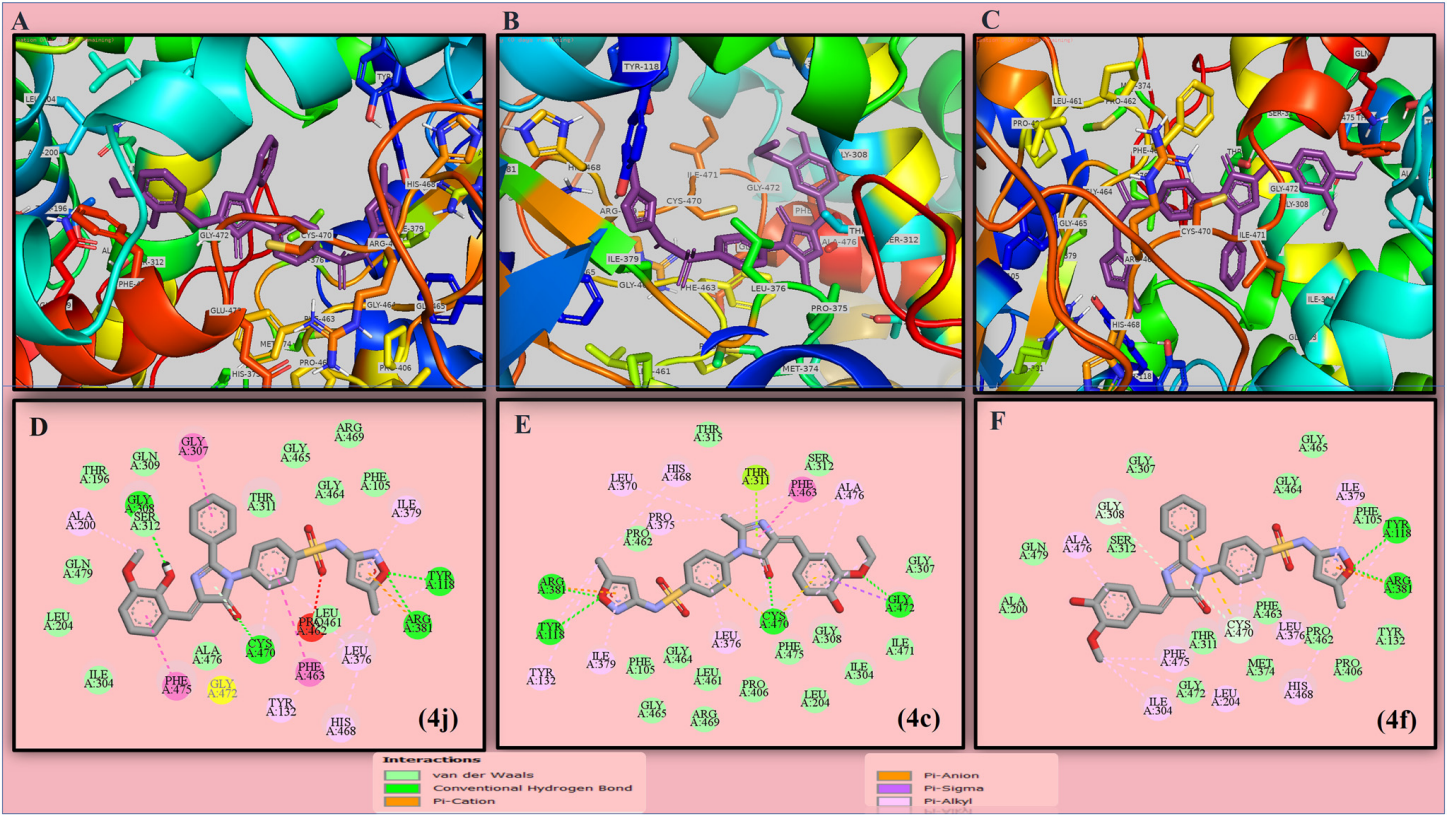
The protein–ligand interactions (A–C) along with their 2D interactions (D–F) of the compounds showing highest docking score against structure of CYP51 from the pathogen *C. albicans* (**5V5Z**).

As per the docking study, results showed a binding energy range between −6 to −12 kcal mol^−1^ in targets and all compounds. Compound 4j has the highest docking score of −10.36 kcal mol^−1^ with the strongest affinity towards the targeted protein. Compounds 4c (−10.29 kcal mol^−1^), 4f (−9.84 kcal mol^−1^) and 4g (−9.59 kcal mol^−1^) show the highest scores against *C. albicans* fungal protein. The docking score of the standard drug ketoconazole is −9.13 kcal mol^−1^ against *C. albicans*, which is similar to those of our lead candidates. The interacting residues and lig-plot of the above-mentioned lead candidates are depicted in Fig. 3.

**Fig. 4:**
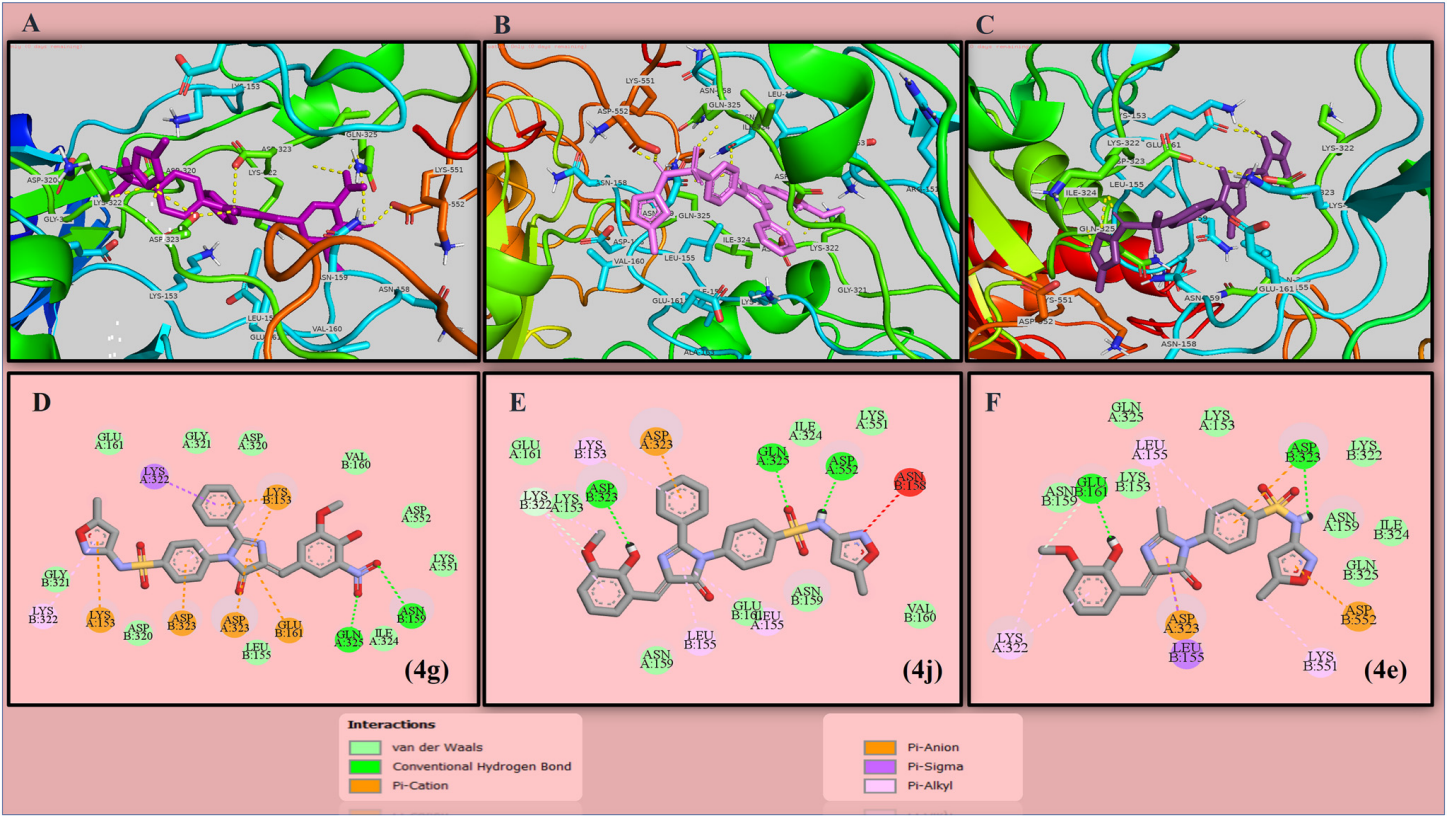
Protein–ligand interactions (A–C) along with their 2D interactions (D–F) of lead candidates against penicillin binding protein 2a from MRSA (**1VQQ**).

In the case of antibacterial findings, compound 4g (−8.62 kcal mol^−1^) has shown the highest binding energy against penicillin-binding protein 2a from MRSA, followed by 4j (−8.56 kcal mol^−1^), 4e (−8.47 kcal mol^−1^) and 4f (−8.39 kcal mol^−1^). The standard drug gentamicin showed a docking result of −10.89 kcal mol^−1^ against this protein. The protein interaction residues and LIG-plot of potent candidates against the bacterial target are depicted in Fig. 4.

4j, 4g and 4f are the lead compounds in this research, showing good affinity towards various protein targets. This research focuses on the antibacterial and antifungal activity of the developed compounds. The protein–ligand interaction study of standard drugs revealed that several hydrogen bonds are present, along with van der Waals pi-alkyl, pi-amide, pi-anion/cation and many other types of bonding interactions. The docking scores of the lead compounds are nearly the same as that of the standard drugs, which signifies that they have good target-specific multipurpose activity and could be treated as potent antimicrobial agents. Among these candidates, compound 4j attains the highest docking score of −10.36 kcal mol^−1^ with four hydrogen bond interactions against a fungal target and the second highest score of −8.56 kcal mol^−1^ with three bonding interactions against the bacterial target PBP 2a of MRSA. Hydrogen bonding interactions were found for fungal targets between the hydroxy group of the vanillyl ring with GLY308, imidazolone ring with CYS470 and two interactions with the isoxazole ring system of the sulfonamide side chain: ARG381 and TYR118. However, in the case of bacterial protein target, ASP323 interacted with the hydroxy group of the vanillin moiety, whereas GLN325 and ASP552 formed hydrogen interactions with SO_2_ and the NH linker of SMZ, respectively. Similarly, compound 4g showed a total of five hydrogen bonding interactions against both targets *viz.* NO_2_ and OH group substituted vanillyl substrate showed bonding interactions with THR311 and GLN479, with the SO_2_ group of the sulfamethoxazole structure binding with ARG381 of the fungal target protein. Also, two prominent interactions were found with the NO_2_ group present in vanillin structure, *i.e.*, ASN159 and GLN325. Compound 4g showed a concomitant activity in the computational analysis as well as the antibacterial assay due to the electron withdrawing effect of the nitro group in the compound. In compound 4f, there are two hydrogen interactions with bacterial and fungal targets. The findings show that the isoxazole ring of the SMZ side chain binds with two amino acids (TYR118 and ARG381) in the fungal target **5V5Z**, whereas the OH group of vanillin and the imidazolone moiety has interactions with ASP552 and ASP323, respectively. Alongside, most of the compounds show interactions with TYR118 and LEU376 in the case of the fungal target, which is a proven active site of the protein. Therefore, we can also state that the docked ligands bound with the targeted protein in their active binding pocket. In the case of standard drugs, gentamicin shows seven conventional hydrogen bonds against the same bacterial target, while ketoconazole shows two hydrogen bond interactions against the same fungal target. From the obtained data, most of the compounds have experienced successful binding interactions by removing the co-crystalized ligand from the protein structure. To claim this, we have conducted a comparative study between co-crystalized ligands attached to protein and removed the ligand protein structure. The docking scores favored the removed ligand structure as a feasible interactive target site, as shown in the ESI† file containing Fig. S65 and S66. The docking scores and interactions of all synthesized candidates and standard drugs against both proteins are described in Table 1.

#### 3.2.2. Physicochemical parameters of the synthesized compounds

The evaluated parameters for validating the physicochemical properties were the primary screening tools used to determine the synthetic compound's activity. After examining a large range of activities, the compounds were evaluated for their physicochemical properties. After examining the various physicochemical parameters, all compounds (3a–3j and 4a–4j) stated here highly satisfied Lipinski's rule of five. All compounds achieved the qualification criteria and showed good results in their respective limits. Previously, all compounds were sketched and screened using ChemDraw, Molinspiration (https://www.molinspiration.com/), and Molsoft (https://molsoft.com/mprop/), which was depicted in Table 2.

**Table 2:** Molecular properties of the newly synthesized oxazolone (3a–3j) and imidazolone (4a–4j) derivatives (RO5)

Compound name	Molecular weight (g mol^−1^)	HA	HB	clog *P*	tPSA
3a	233	5	1	1.7	98
3b	278	7	1	1.62	112
3c	247	5	1	2.1	104
3d	275	6	0	1.9	115
3e	233	5	1	1.7	98
3f	295	5	1	2.7	126
3g	340	7	1	2.6	141
3h	309	5	1	3.1	133
3i	337	6	0	2.9	143
3j	295	5	1	2.7	126
4a	468	9	2	3.96	142
4b	513	10	2	4.39	188
4c	482	9	2	4.35	142
4d	496	10	2	3.88	159
4e	468	9	2	3.96	142
4f	530	9	2	4.99	142
4g	575	10	2	5.42	188
4h	544	9	2	5.38	142
4i	558	10	2	4.9	159
4j	530	9	2	4.99	142

Further, virtual calculation of the compounds adsorption, distribution, metabolism, and elimination included several parameters *viz.*, the blood–brain barrier (BBB) for evaluating the permeability, while Caco-2 permeability predicted the oral drug absorption ability. Another measure for absorption was human intestinal absorption, while skin permeability was measured statistically, which could gather sufficient data for *in silico* analysis of the synthesized imidazolone–oxazolone congeners [https://preadmet.webservice.bmdrc.org/adme/]. Along with the pharmacokinetic parameters, the compounds toxicity and lethal dose LD_50_ were evaluated. The range theoretically varies from 150 to 1500 mg kg^−1^ and the class varied from I to VI, determined through online software ProTox (https://tox.charite.de/protox3/#) depicted in Table 3.

**Table 3:** Pharmacokinetic (pre-ADMET) profiles of oxazolone (3a–3j) and imidazolone (4a–4j) derivatives

Name	BBB	Caco-2 permeability	HIA	Skin permeability	LD_50_ (mg kg^−1^)	Toxicity class
3a	0.6	22.1	93.4	−3.7	978	4
3b	0.17	20.2	55.5	−3.86	1400	4
3c	0.08	25.8	94.2	−3.7	978	4
3d	0.19	28.1	95.4	−3.8	978	4
3e	0.62	4.13	93.4	−3.7	978	4
3f	0.09	24.4	96.4	−3.2	978	4
3g	0.03	21.01	88.7	−3.2	1400	4
3h	0.1	31.8	96.4	−3.09	978	4
3i	0.24	32.7	99.0	−3.1	1400	4
3j	0.12	21.7	96.4	−3.22	978	4
4a	0.043	2.75	95.03	−3.19	3471	5
4b	0.049	0.411	79.02	−3.01	3471	5
4c	0.044	5.34	95.37	−3.05	3471	5
4d	0.053	1.13	93.05	−3.10	3471	5
4e	0.043	2.75	95.03	−3.17	3471	5
4f	0.035	7.93	95.92	−2.62	1000	4
4g	0.062	0.53	91.65	−2.51	600	4
4h	0.032	11.30	95.85	−2.55	1000	4
4i	0.059	3.52	96.53	−2.55	1000	4
4j	0.032	7.93	95.92	−2.61	1000	4

### 3.3. Antimicrobial activity of synthesized congeners

Initially, two series of compounds oxazolones (3a–3j) and imidazolones (4a–4j) were synthesized. To evaluate their optimal antimicrobial potency, a total six multidrug resistance (MDR) strains were procured from clinical samples of SUM Hospital, Bhubaneswar, India. They were further isolated and stored in a suitable environment to obtain better results in terms of inhibition by the new compounds. Among these six samples, four samples were intended for bacterial activity *viz. Staphylococcus aureus*, *Streptococcus pyogenes*, *Klebsiella pneumoniae*, *Escherichia coli* and two other topical fungal strains include *Candida tropicalis* and *Trichophyton rubrum*. To strengthen the experimental results with theoretical claims, two marketed drugs (gentamicin and ketoconazole) were taken as standards for antibacterial and antifungal activity, respectively, along with the synthesized compounds. The results indicated that all candidates showed mild to good zones of inhibition (ZOI) and minimum inhibitory concentration (MIC). Among all the compounds, 4g and 4d showed maximum zones of inhibition of 24 and 22 mm against *S. aureus* and *S. pyogenes*, respectively, and 18 and 19 mm against *K. pneumoniae* and *E. coli*, respectively. Compound 4g has a moderate to good zone of inhibition range of 15–24 mm, whereas compound 4d has 21, 22, 16 and 19 mm zones of inhibition against the bacterial strains *S. aureus*, *S. pyogenes*, *K. pneumonia*, and *E. coli*, respectively. Both compounds showed comparatively better activity than the standard drug gentamicin and moderate inhibition against fungal strains *C. tropicalis* and *T. rubrum*. Basing upon the ZOI results of the candidates, we have carried out MIC with *S. aureus* and *K. pneumoniae* bacterial strains where the compounds have shown good inhibition. Compound 4g has a significant result against both strains with a MIC value of 6.25 μg mL^−1^, whereas compound 4d has values of 12.5 μg mL^−1^ and 50 μg mL^−1^ against MDR *K. pneumoniae* and MDR *S. aureus*, respectively. Compounds 3i, 3j and 4i have moderate inhibition and no significant MIC values against both strains. In the case of fungal results, compounds 3a, 3c, 4h, 4e and 3i have effective inhibition values of 19 and 20 mm against *C. tropicalis* and *T. rubrum*, respectively, which are comparably better than standard values of ketoconazole. All synthesized compounds have a substituted benzylidene moiety in common but a substitution of 5-methyl-isoxazolyl benzenesulfonamide was done on imidazolones. The SARs indicate that compound 4g—which has a *meta* positioned nitro substituted benzylidenyl group attached to an isoxazolyl bearing sulfonamide through an imidazolone moiety—could have significant action against MDR bacterial strains.

The presence of these specific features at the C-4 and N-1 positions of the imidazolone ring is likely responsible for the significant inhibition of resistant strains. Among these synthesized compounds, imidazolones have superior antibacterial activity compared with oxazolones. However, certain oxazolones showed better activity against topical fungal strains than their imidazolone counterparts. The relevant antimicrobial results of all newly synthesized compounds are depicted in Table 4.

**Table 4:** Antimicrobial assessment of oxazolone (3a–3j) and imidazolone (4a–4j) candidates against different MDR pathogens through their zone of inhibition (ZOI) and minimum inhibitory concentration (MIC) against MDR *S. aureus* and *K. pneumoniae*

Compound name	Antimicrobial assessment by ZOI (mm) and MIC (μg mL^−1^)							
	*S. aureus*		*S. pyogenes*	*K. pneumoniae*		*E. coli*	*C. tropicalis*	*T. rubrum*
	ZOI	MIC	ZOI	ZOI	MIC	ZOI	ZOI	ZOI
3a	9	25	11	10	50	8	**19**	16
3b	7	25	12	11	50	10	18	14
3c	9	25	12	10	50	9	**19**	17
3d	8	50	10	9	50	8	18	17
3e	8	50	8	7	50	8	17	16
3f	7	100	7	8	25	9	18	16
3g	8	50	8	6	25	14	13	10
3h	9	50	8	13	25	11	17	18
3i	15	NA	9	14	NA	12	18	**20**
3j	13	NA	10	15	NA	12	13	13
4a	20	25	11	13	25	12	18	17
4b	17	50	13	17	25	17	14	14
4c	R	25	R	15	25	18	18	13
4d	21	50	**22**	16	12.5	**19**	11	10
4e	R	50	15	17	5	17	**19**	14
4f	19	50	18	14	25	15	14	12
4g	**24**	6.25	**22**	**18**	6.25	15	12	12
4h	R	50	13	14	50	14	**19**	18
4i	14	NA	14	15	NA	13	13	12
4j	R	NA	17	14	NA	13	16	14
Gentamycin	24	50	22	21	50	24	—	—
Ketoconazole	—	—	—	—	—	—	16	13

### 3.4. HOMO–LUMO analysis

**Fig. 5:**
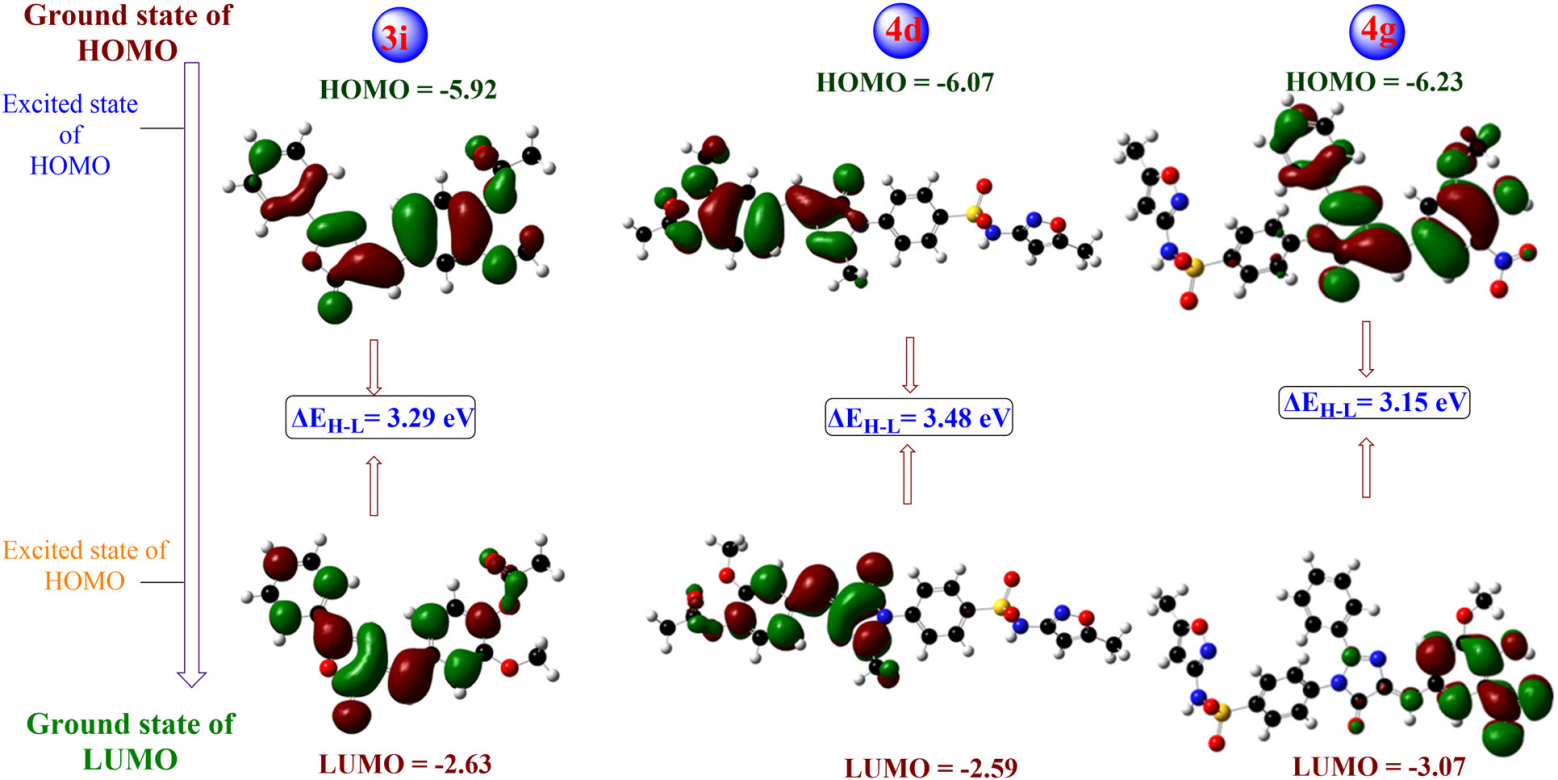
HOMO–LUMO analysis and energy gap of effective congeners.

The HOMO and LUMO both are considered as vital tools in the computational investigations of the physicochemical, pharmacokinetic, and toxicological properties. These orbitals are critical in predicting molecular reactivity, stability, and interactions with biological targets. The compounds with higher *E*_HOMO_ values are typically potent electron donors, with their energy levels closely related to their ionization potential (IP). For instance, compound 4g—which features nitro substituted vanillin—exhibited the lowest IP value of 6.23 eV among the studied compounds 3i, 4d and 4g, underscoring its strong electron-donating capability displaying the highest *E*_HOMO_ of −6.23 eV. Conversely, compounds with lower *E*_LUMO_ values act as effective electron acceptors, where the energy corresponds to electron affinity (*E*_a_). Among the series, compound 4d demonstrated the highest *E*-gap (Δ*E*) value of 3.48 eV, making it a strong electron acceptor with the lowest *E*_LUMO_ of −2.59 eV (Fig. 5). According to the literature, lower energy gap values (Δ*E* = *E*_LUMO_ − *E*_HOMO_) will lead to good inhibition efficiency because the energy required to remove an electron from the last occupied orbital is small. From this study results, compound 4g has the highest chemical reactivity because of its smallest energy gap of 3.15 eV, making it the most prominent candidate among the tested series.^29^

From the obtained energies of the compounds, some other parameters were evaluated as described below and depicted in Table 5.

**Table 5:** Some crucial measurements gained from HOMO–LUMO analysis

Compound	HOMO	LUMO	Energy gap (Δ*E*)	Ionization potential (IP)	Electron affinity (*E*_a_)	Hardness (*η*)	Softness (*S*)
3i	−5.92	−2.63	3.29	5.92	2.63	1.645	0.607
4d	−6.07	−2.59	3.48	6.07	2.59	1.740	0.574
4g	−6.23	−3.07	3.15	6.23	3.07	1.575	0.634

• Ionization potential (IP) = −*E*_HOMO_

• Electron affinity (*E*_a_) = −*E*_LUMO_

• Hardness (*η*) = (½) (*E*_LUMO_ − *E*_HOMO_)

• Softness (*S*) = 1/*η*

## 4. Conclusion

This research work focused on the synthesis and exploration of azlactone (3a–3j) and imidazolone (4a–4j) compounds, which includes computational studies and antimicrobial activity determining the zone of inhibition and MIC, with compounds 4d and 4g showing potent activities in terms of docking results as well as *in vitro* antibacterial activity against MDR strains of *S. aureus*, *K. pneumoniae*, *E. coli*, and *S. pyogenes*, while compound 3i has potency against fungal strains of *C. tropicalis* and *T. rubrum*, which have almost similar results to the standard drugs gentamycin and ketoconazole. The other drug stability factors also showed significant results, which proves that these compounds are leaders in the field. On a final note, we can conclude that the compounds bearing sulfonamide substituted with an imidazole ring have a comparatively better capability to tackle MDR infections and can be carried forward for *in vivo* assessment and eventually moved to clinical uses.

## Author contributions

Preetesh Kumar Panda: writing – original draft, methodology, validation, and software; Kakarla Pakeeraiah: writing – review & editing, software, formal analysis, and validation; Suvadeep Mal: writing – review & editing, formal analysis, and validation; Monalisa Mahapatra: writing – review & editing, formal analysis, and validation; Ajit Kumar Bishoyi: methodology, formal analysis, and validation; Sudhir Kumar Paidesetty: conceptualization, writing – review & editing, supervision, project administration, and funding acquisition.

## Conflicts of interest

There are no conflicts to declare.

## Supplementary Material

MD-016-D5MD00221D-s001

## Data Availability

Data for this article are available in the main text of the manuscript and in the ESI† file.
