# Supplementary material for: Design, synthesis and computational approach of vanillyl–imidazolidinyl–sulfamethoxazole derivatives as potent antimicrobial candidates tackling microbial resistance
Source: RSC Med Chem. 2025 Jun 6;16(8):3799–813. doi: 10.1039/d5md00221d (PMC12183569; doi:10.1039/d5md00221d)
Supplement: MD-016-D5MD00221D-s001 [file MD-016-D5MD00221D-s001.pdf]

Supplementary Information (SI) for RSC Medicinal Chemistry.
This journal is © The Royal Society of Chemistry 2025

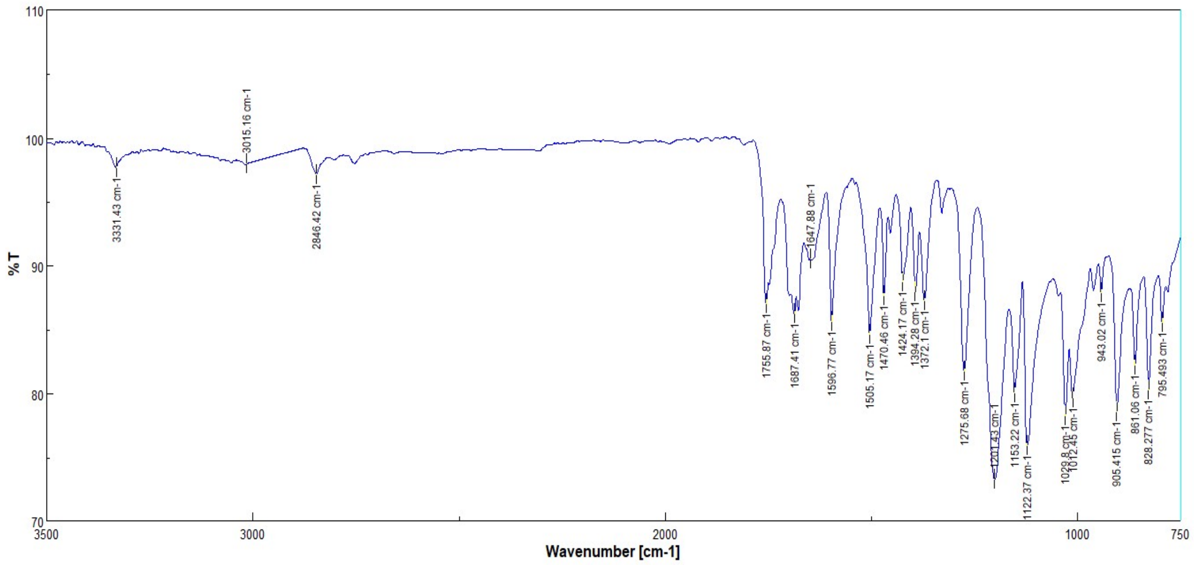

Fig. S1. FT-IR spectra of **3a**

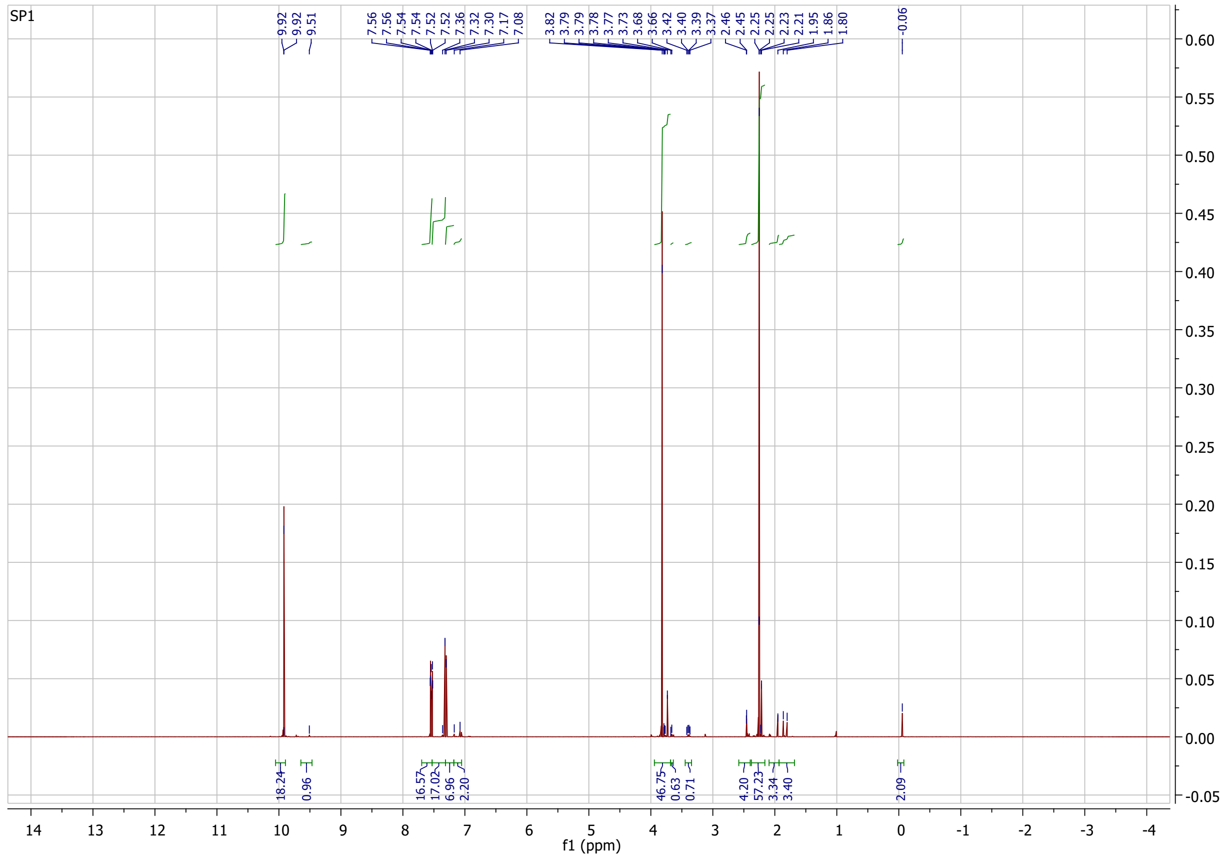

Fig. S2. ^{1}H NMR spectra of **3a**

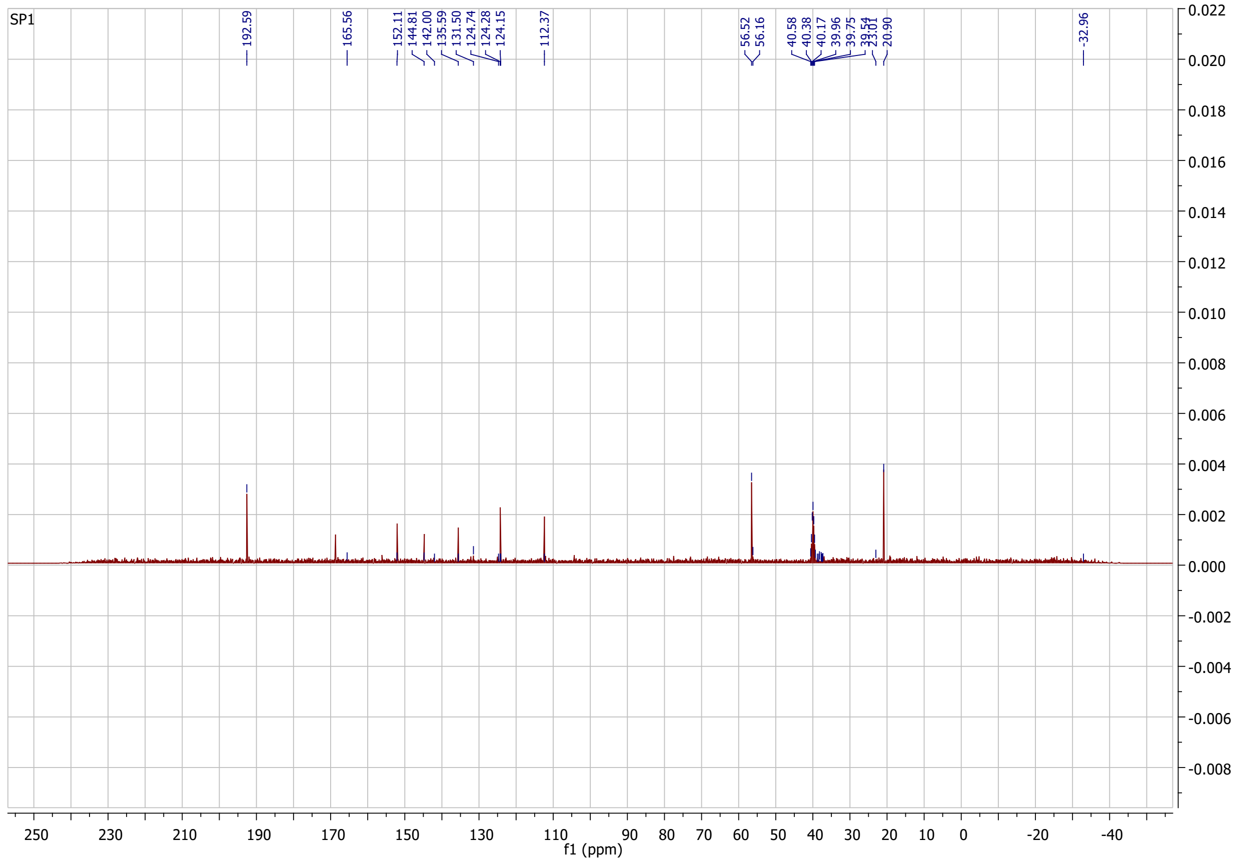

Fig. S3. ^{13}C NMR spectra of **3a**

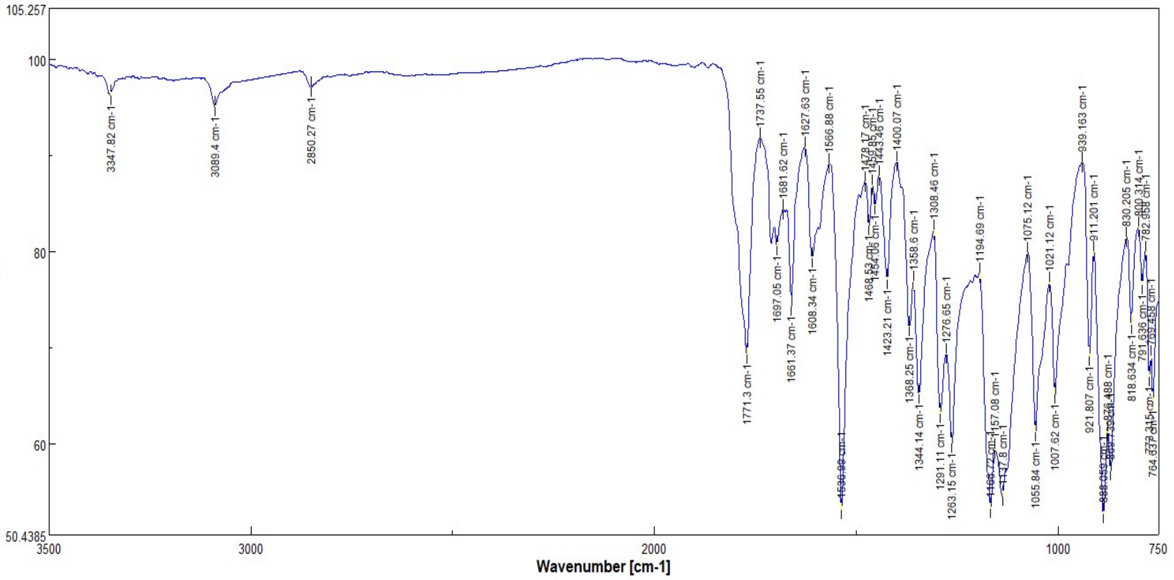

Fig. S4. FT-IR spectra of **3b**

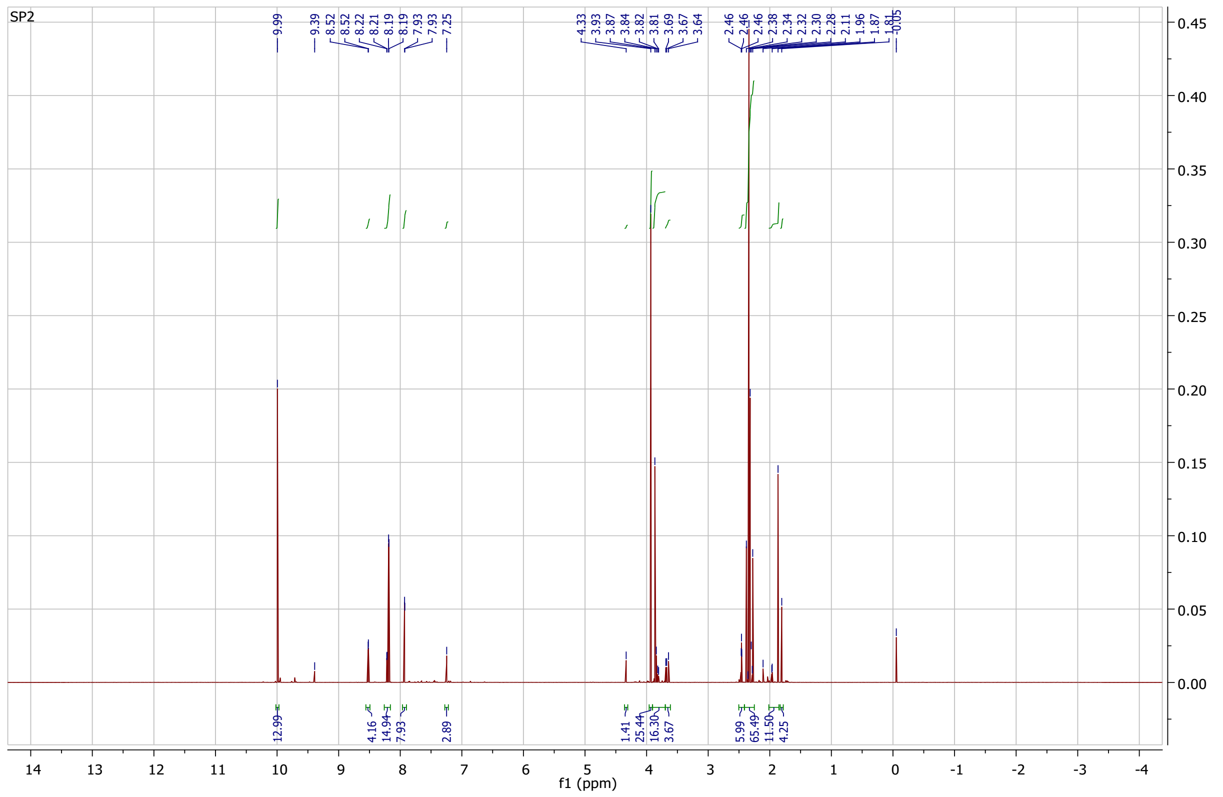

Fig. S5. ^{1}H NMR spectra of **3b**

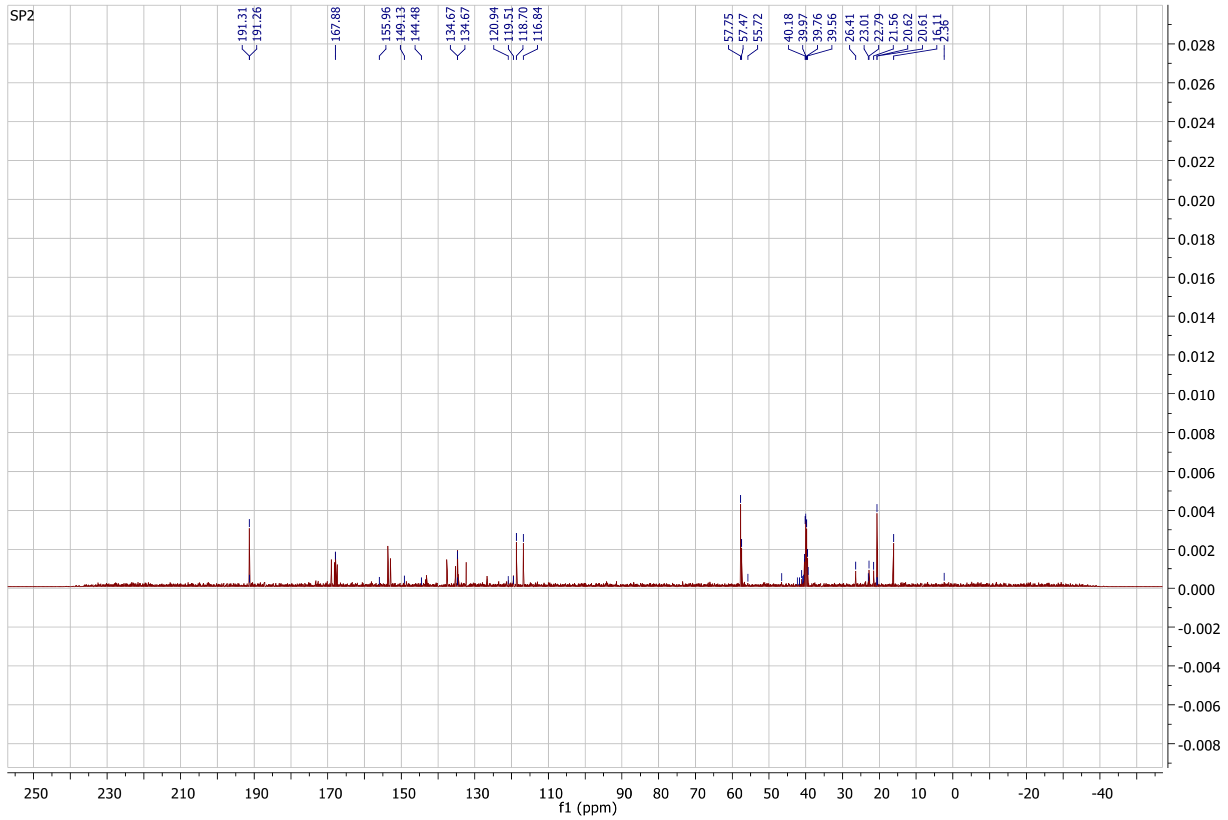

Fig. S6. ^{13}C NMR spectra of **3b**

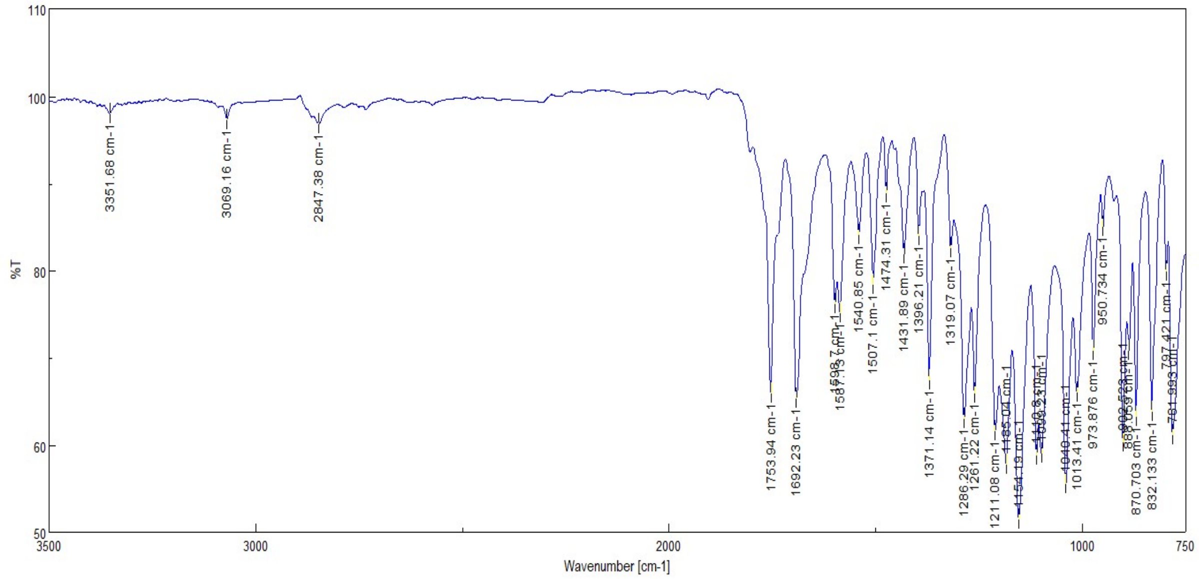

Fig. S7. FT-IR spectra of **3c**

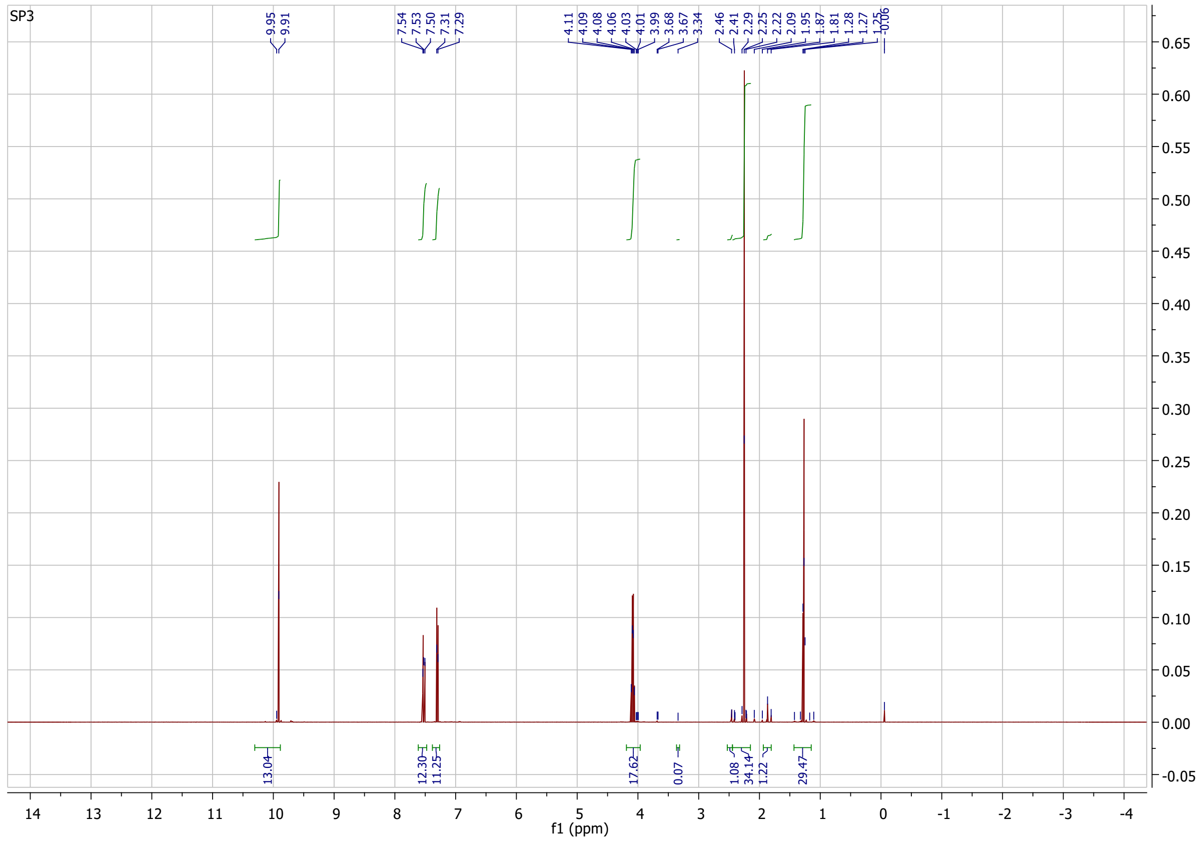

Fig. S8. ^{1}H NMR spectra of **3c**

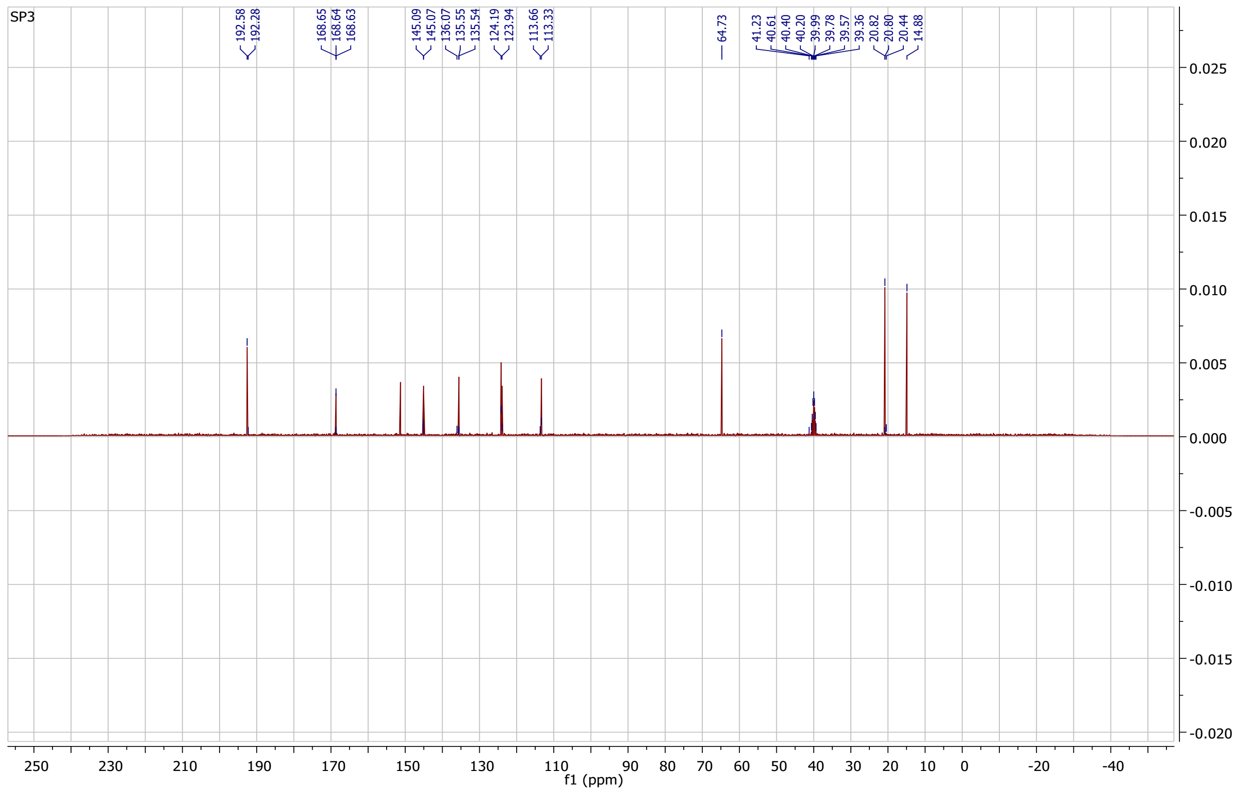

Fig. S9. ^{13}C NMR spectra of **3c**

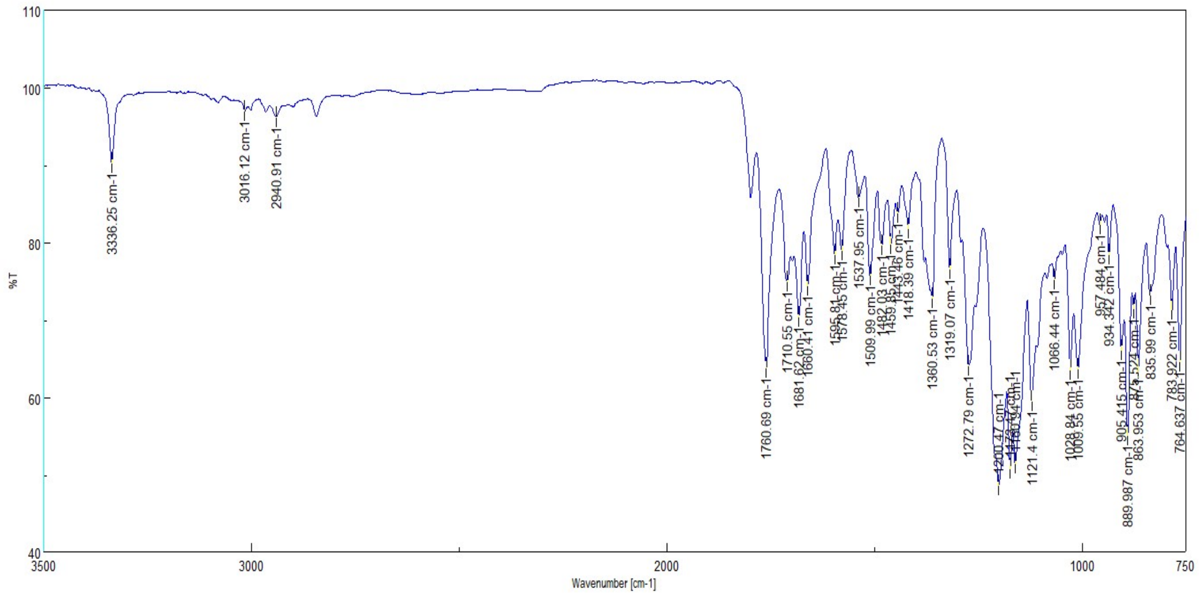

Fig. S10. FT-IR spectra of **3d**

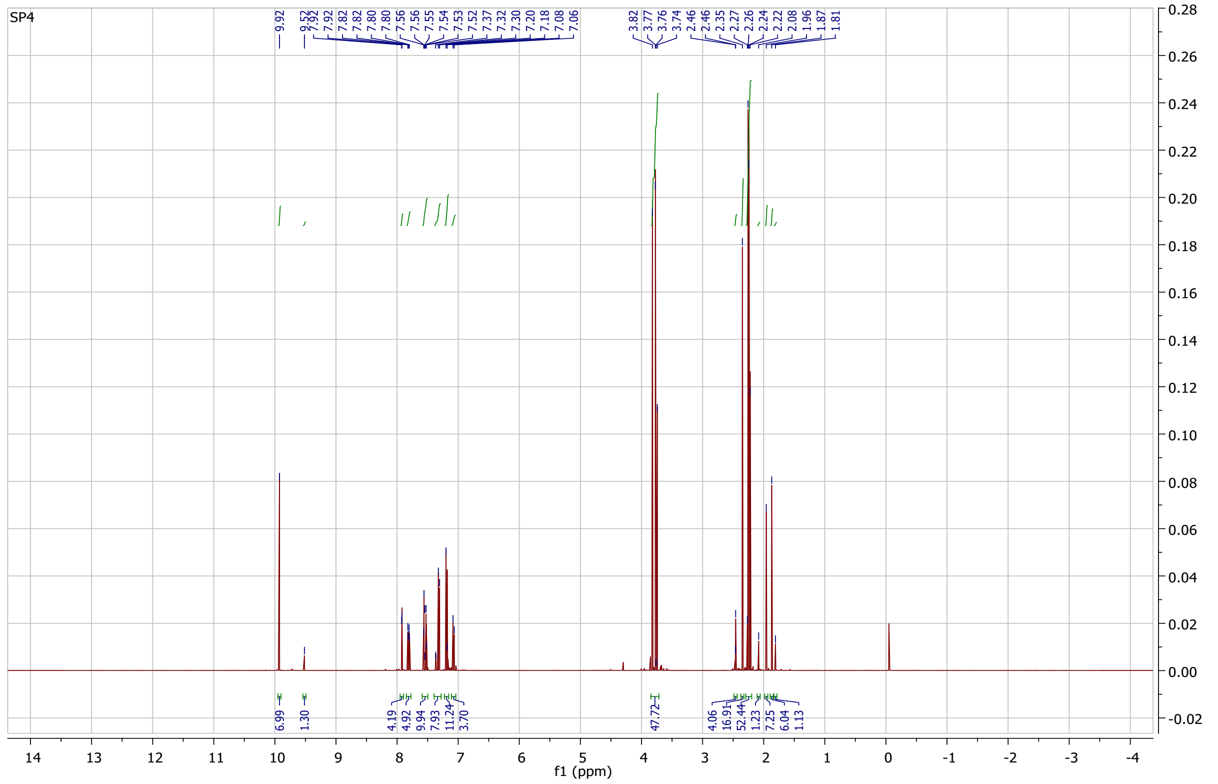

Fig. S11. ^{1}H NMR spectra of **3d**

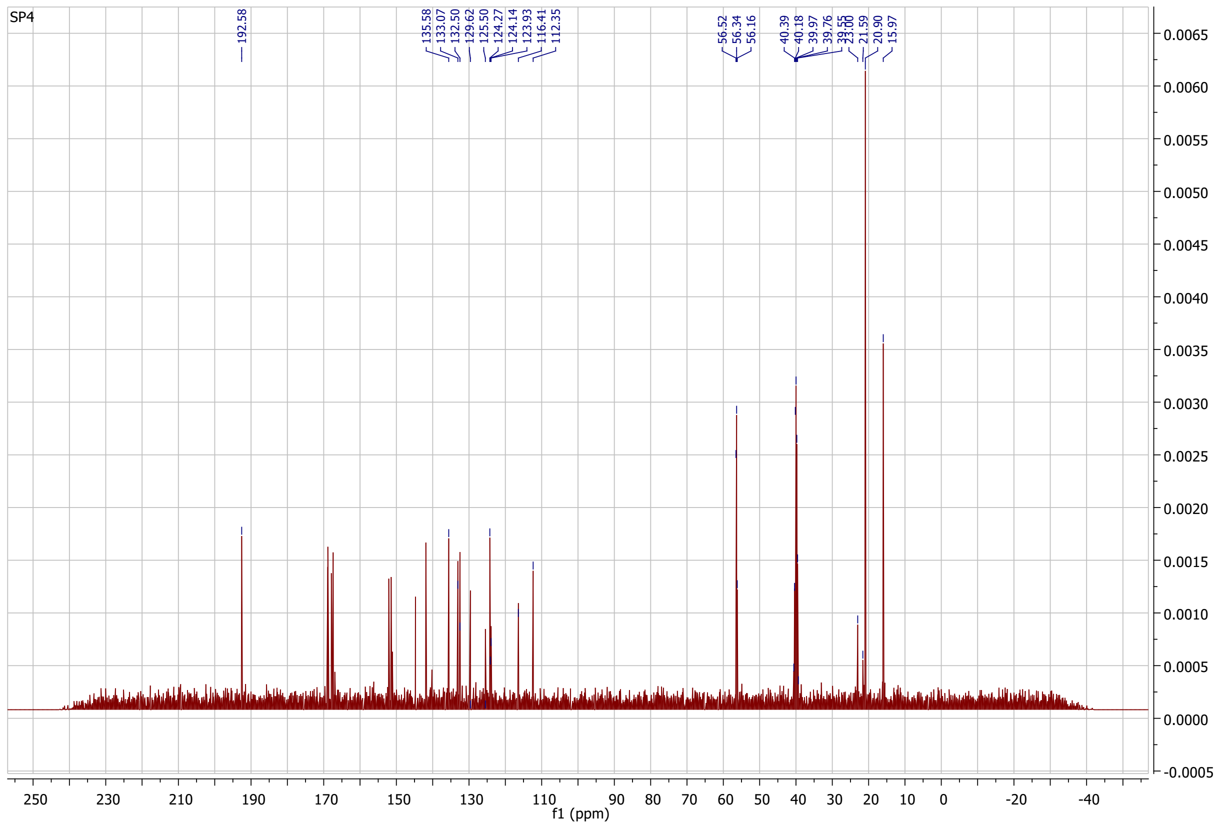

Fig. S12. ^{13}C NMR spectra of **3d**

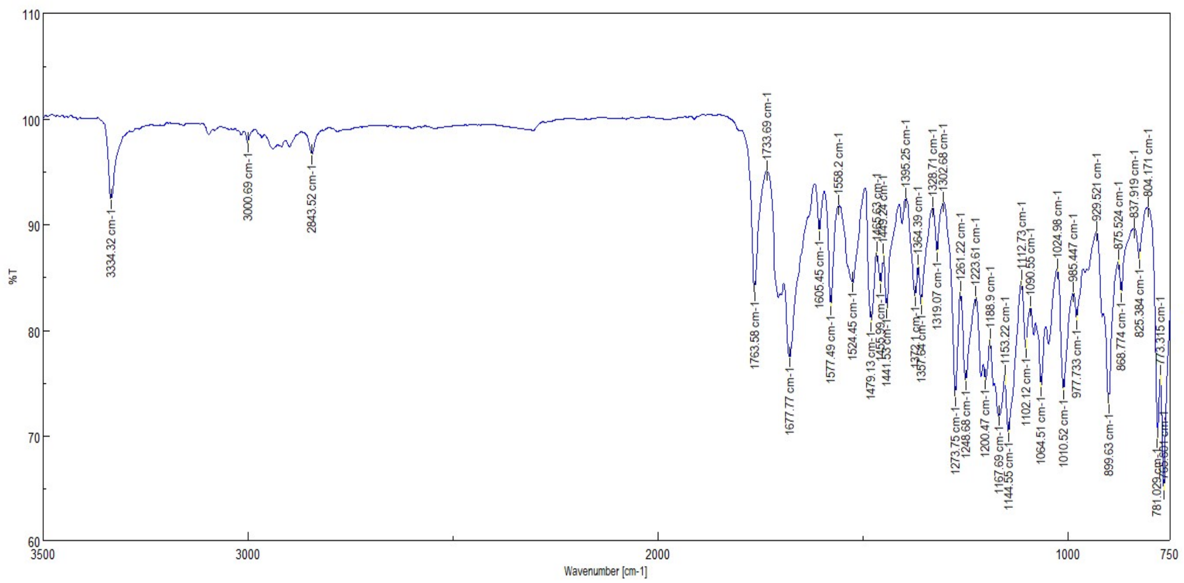

Fig. S13. FT-IR spectra of **3e**

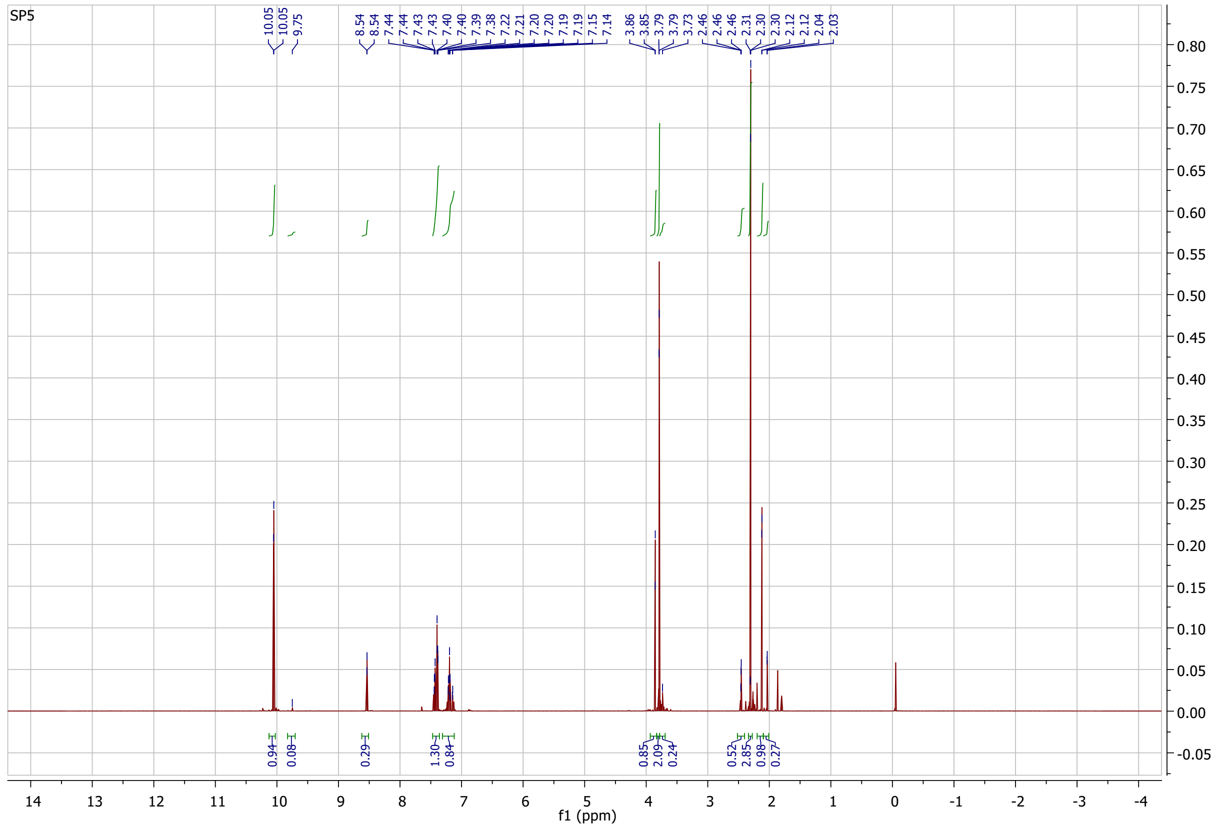

Fig. S14. ^{1}H NMR spectra of **3e**

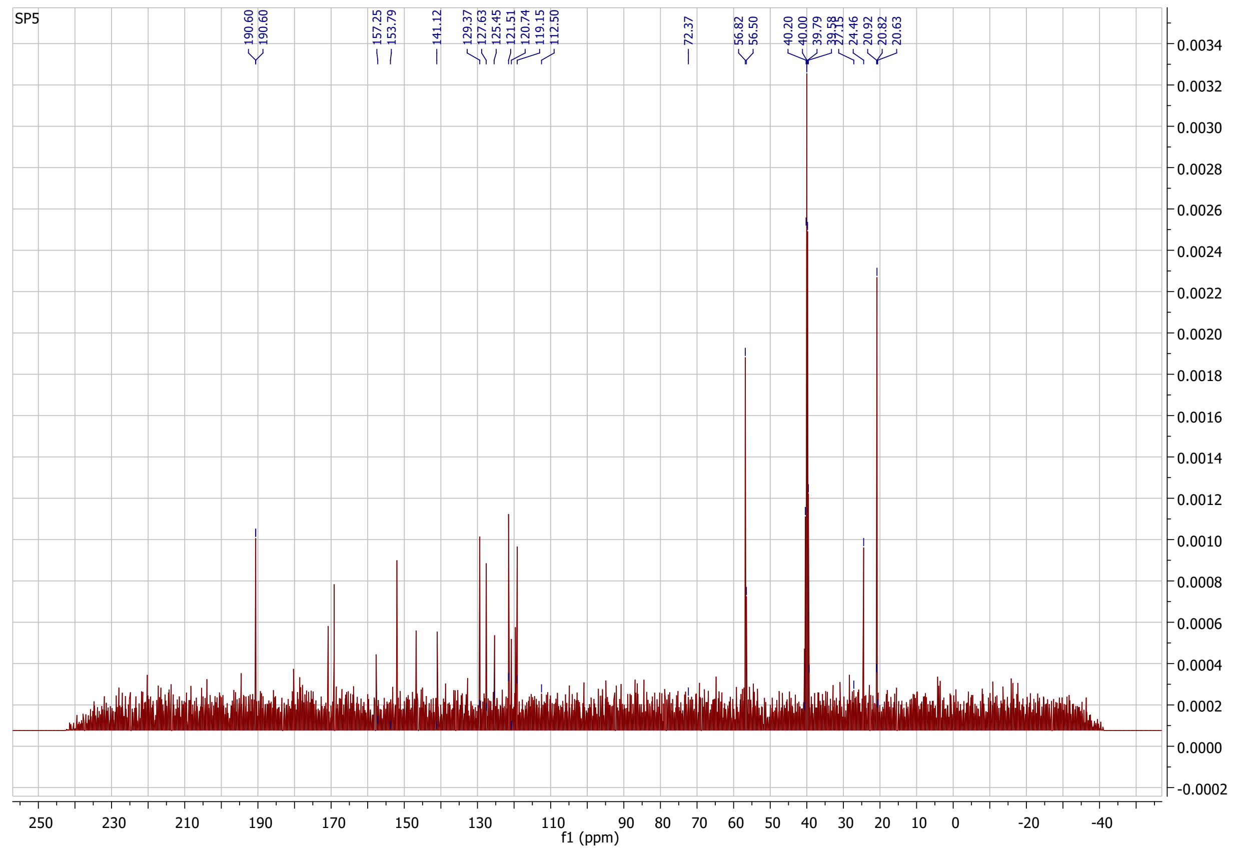

Fig. S15. ^{13}C NMR spectra of **3e**

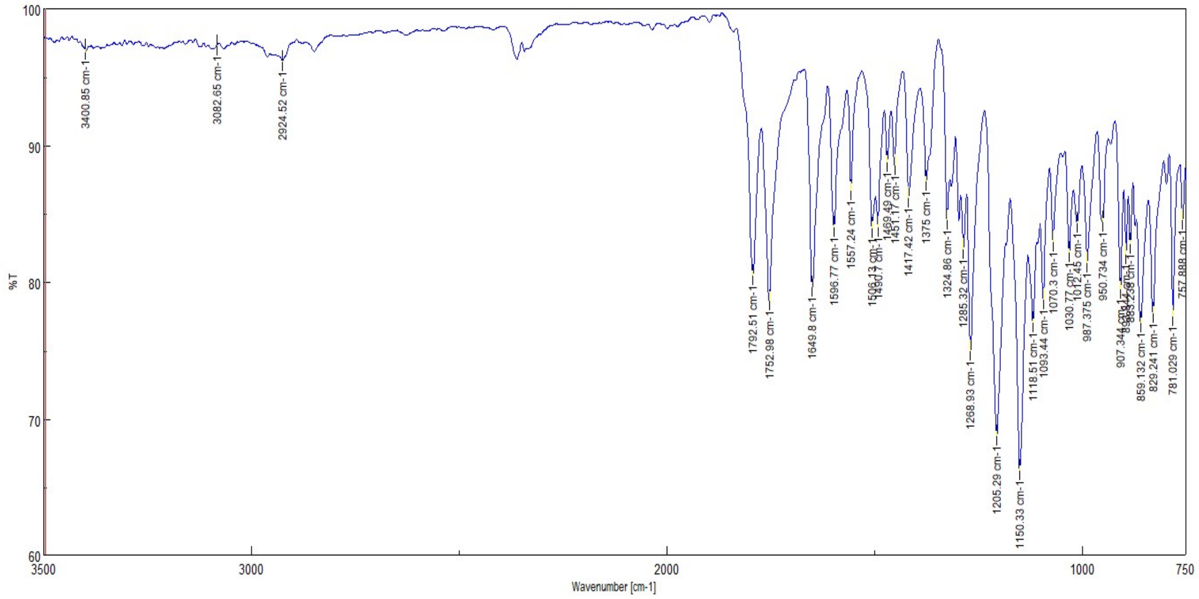

Fig. S16. FT-IR spectra of **3f**

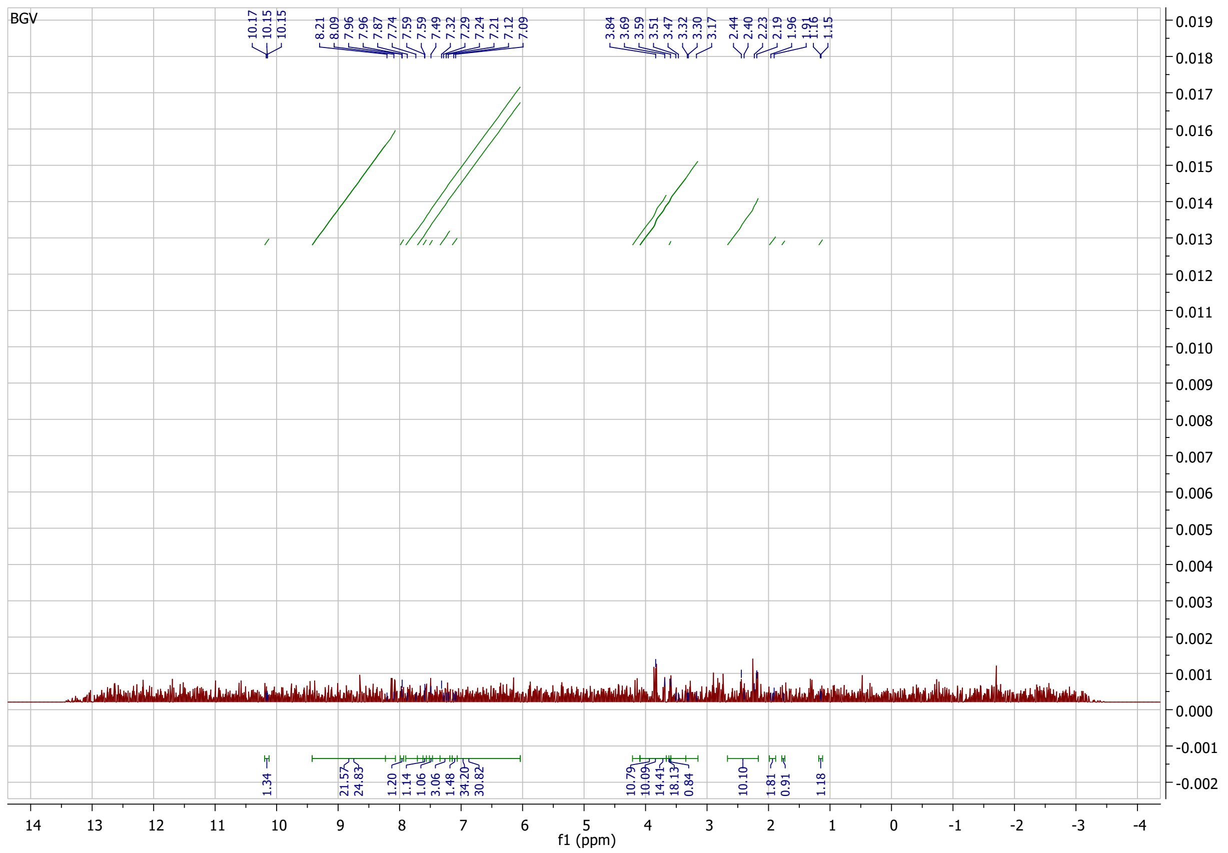

Fig. S17. ^{1}H NMR spectra of **3f**

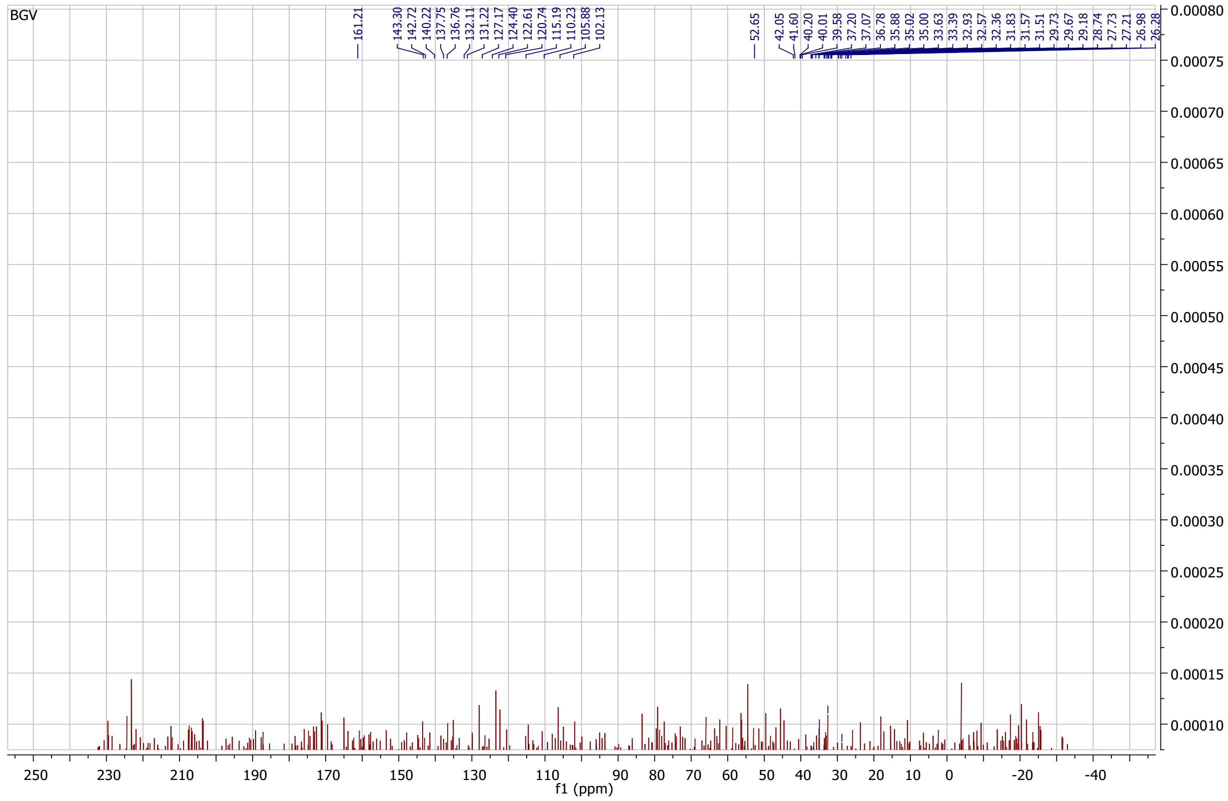

Fig. S18. ^{13}C NMR spectra of **3f**

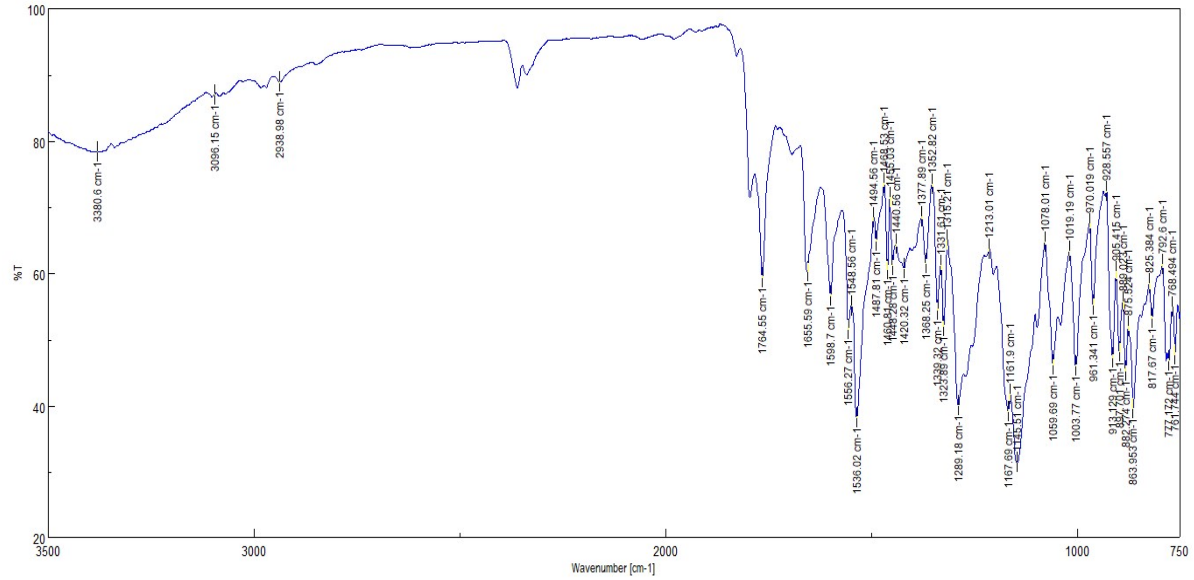

Fig. S19. FT-IR spectra of **3g**

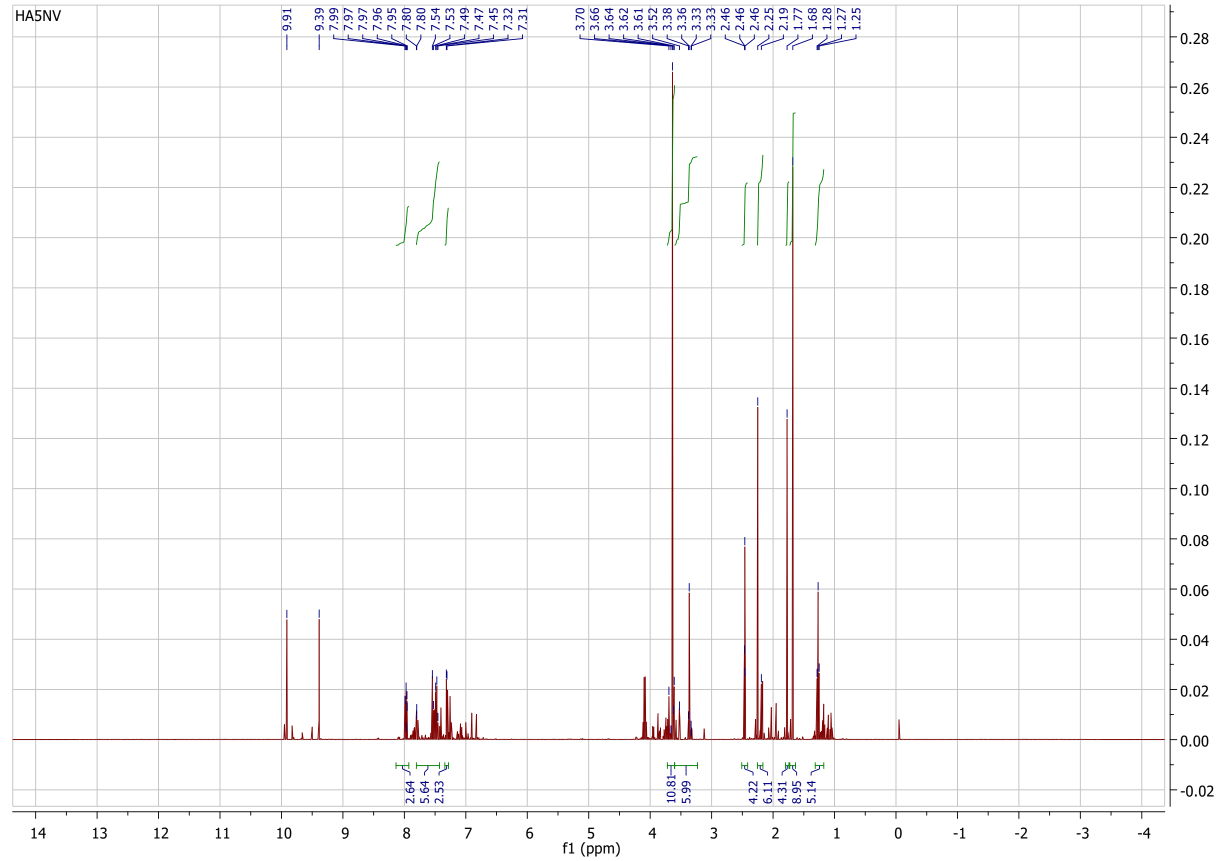

Fig. S20. ^{1}H NMR spectra of **3g**

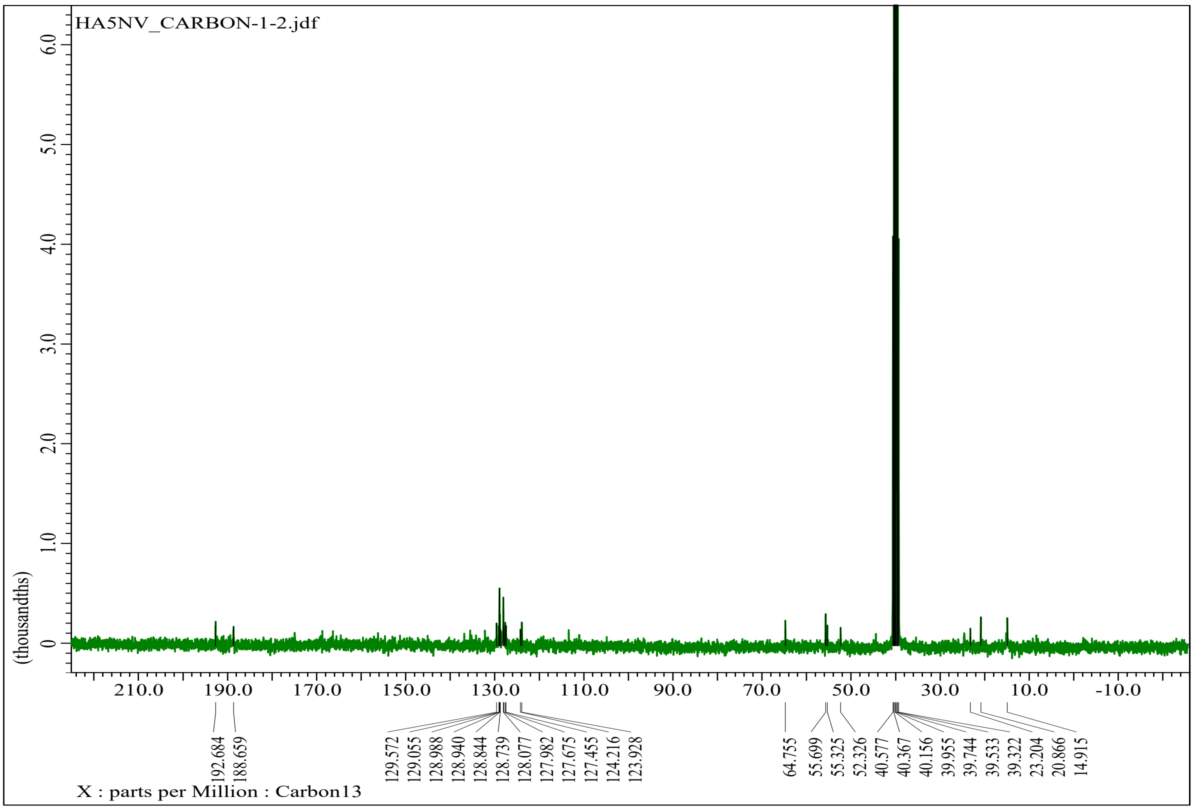

Fig. S21. ^{13}C NMR spectra of **3g**

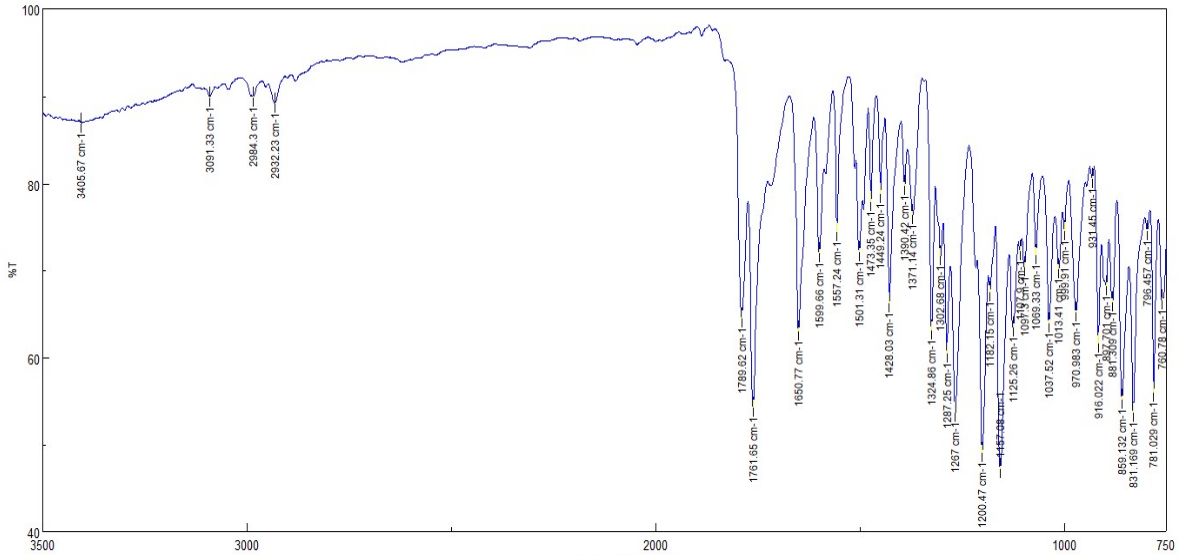

Fig. S22. FT-IR spectra of **3h**

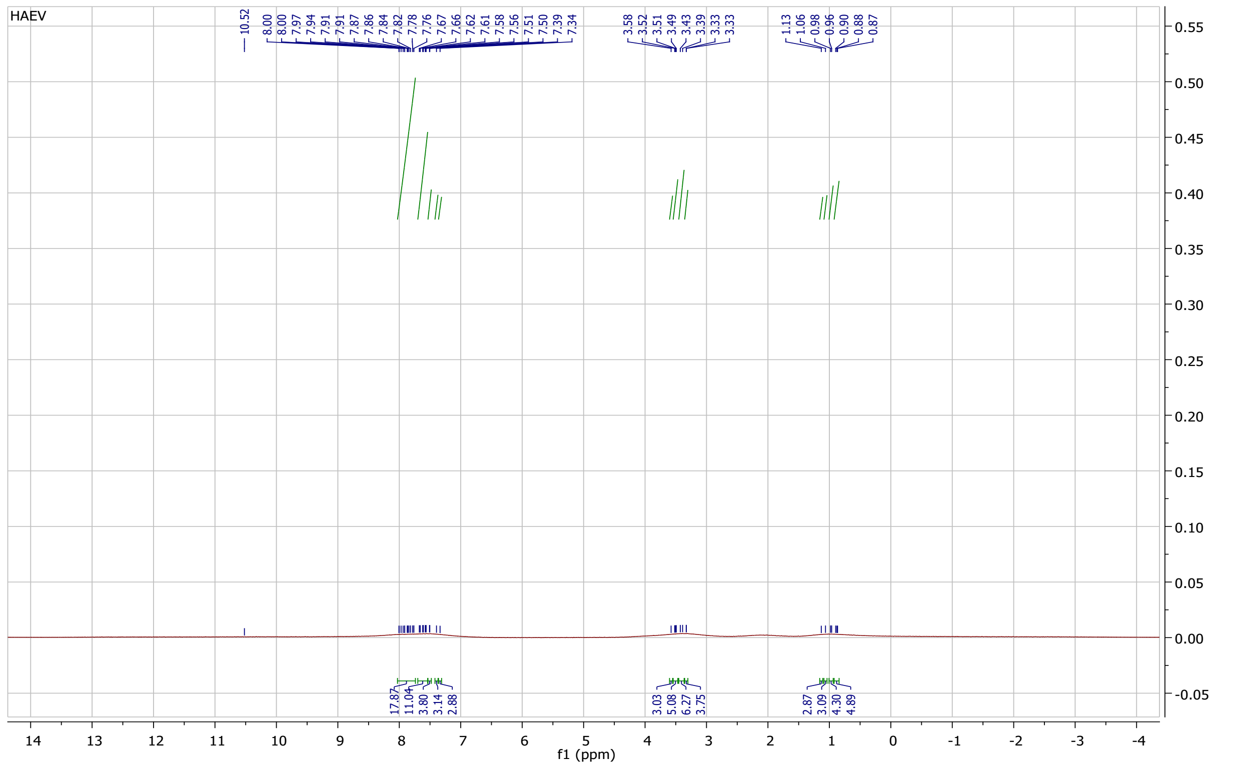

Fig. S23. ^{1}H NMR spectra of **3h**

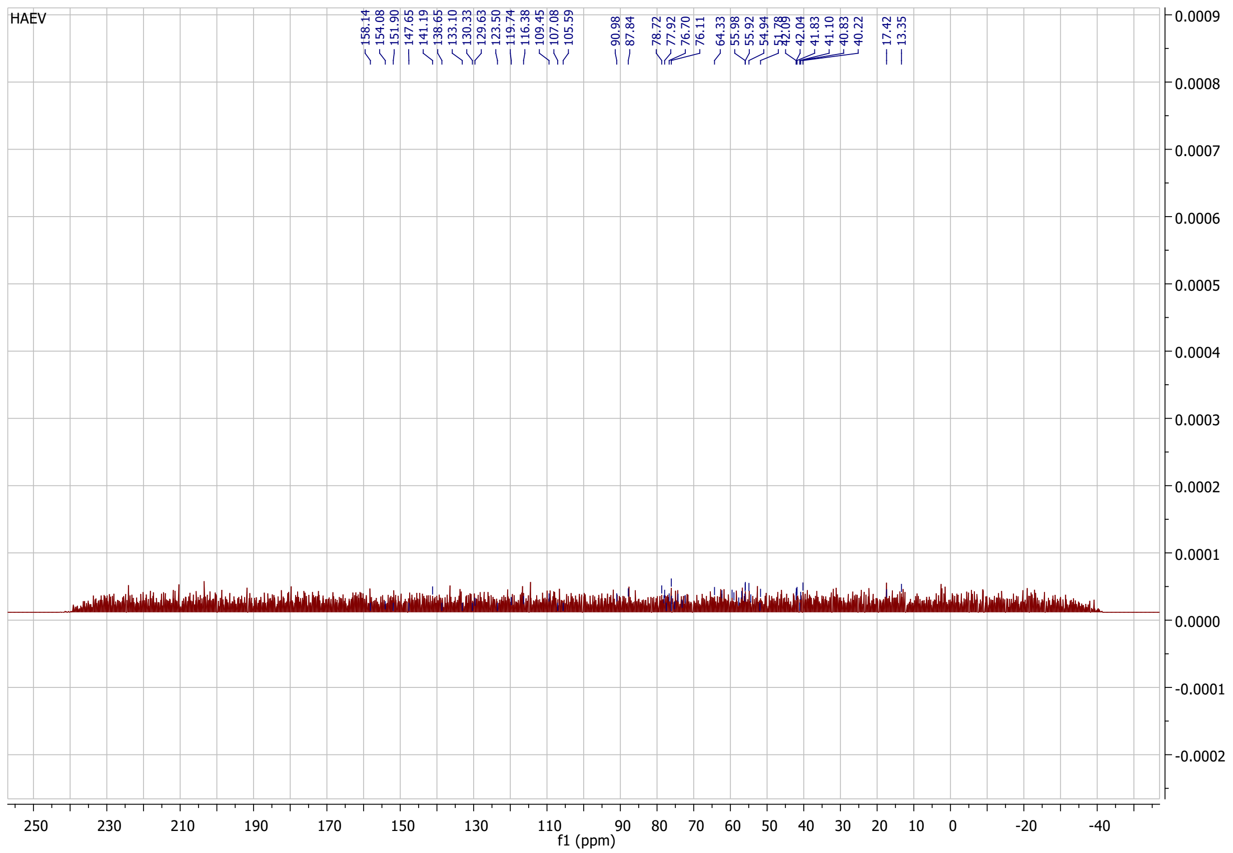

Fig. S24. ^{13}C NMR spectra of **3h**

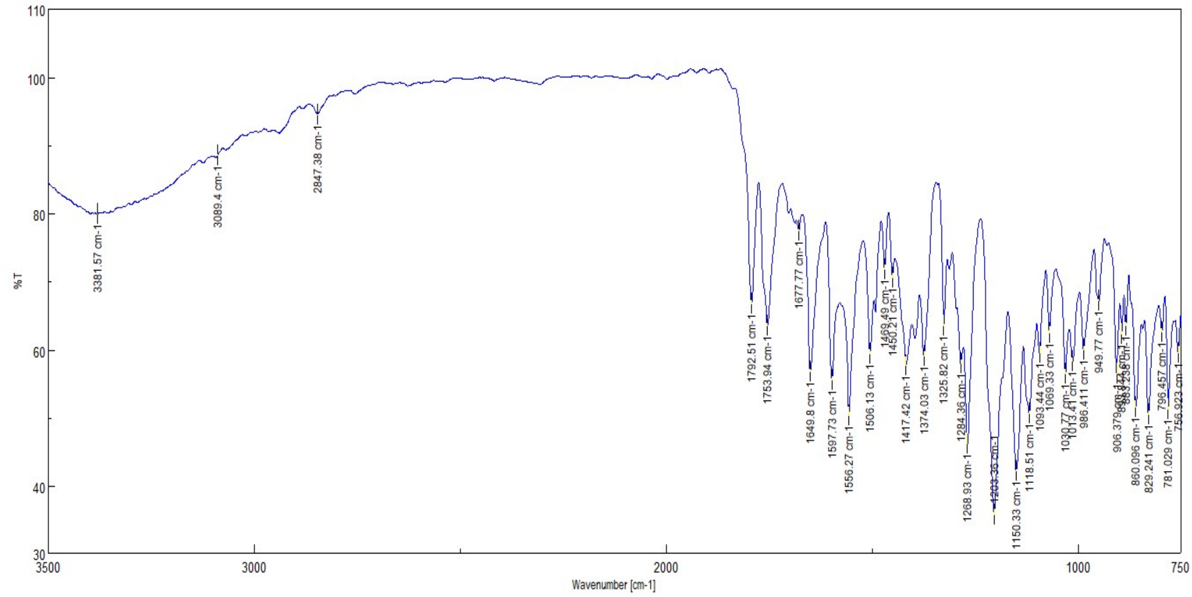

Fig. S25. FT-IR spectra of **3i**

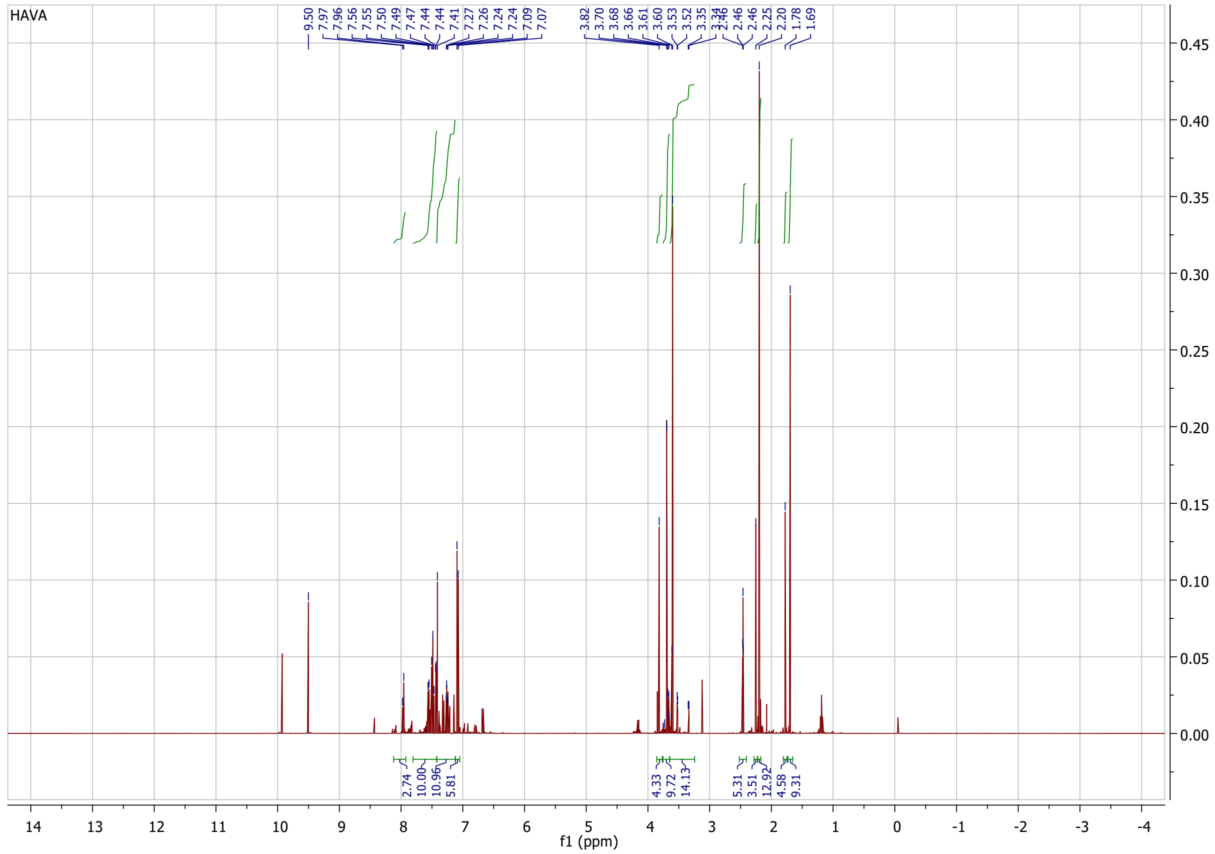

Fig. S26. ^{1}H NMR spectra of **3i**

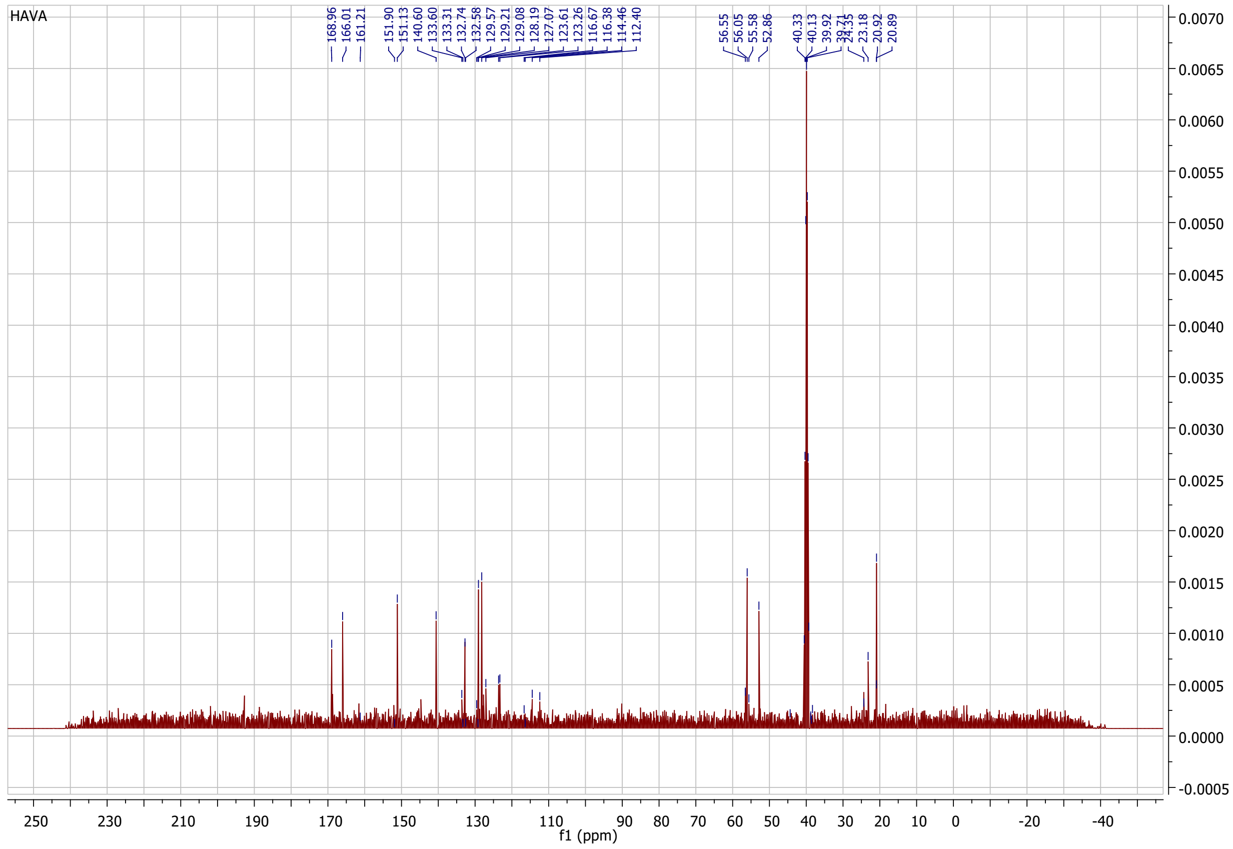

Fig. S27. ^{13}C NMR spectra of **3i**

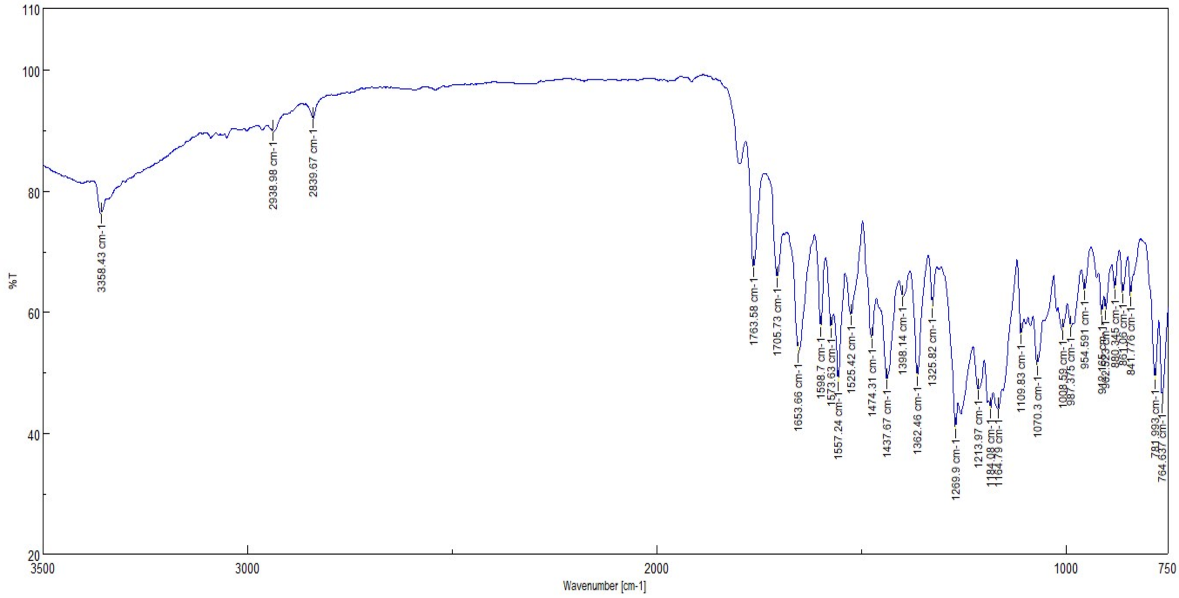

Fig. S28. FT-IR spectra of **3j**

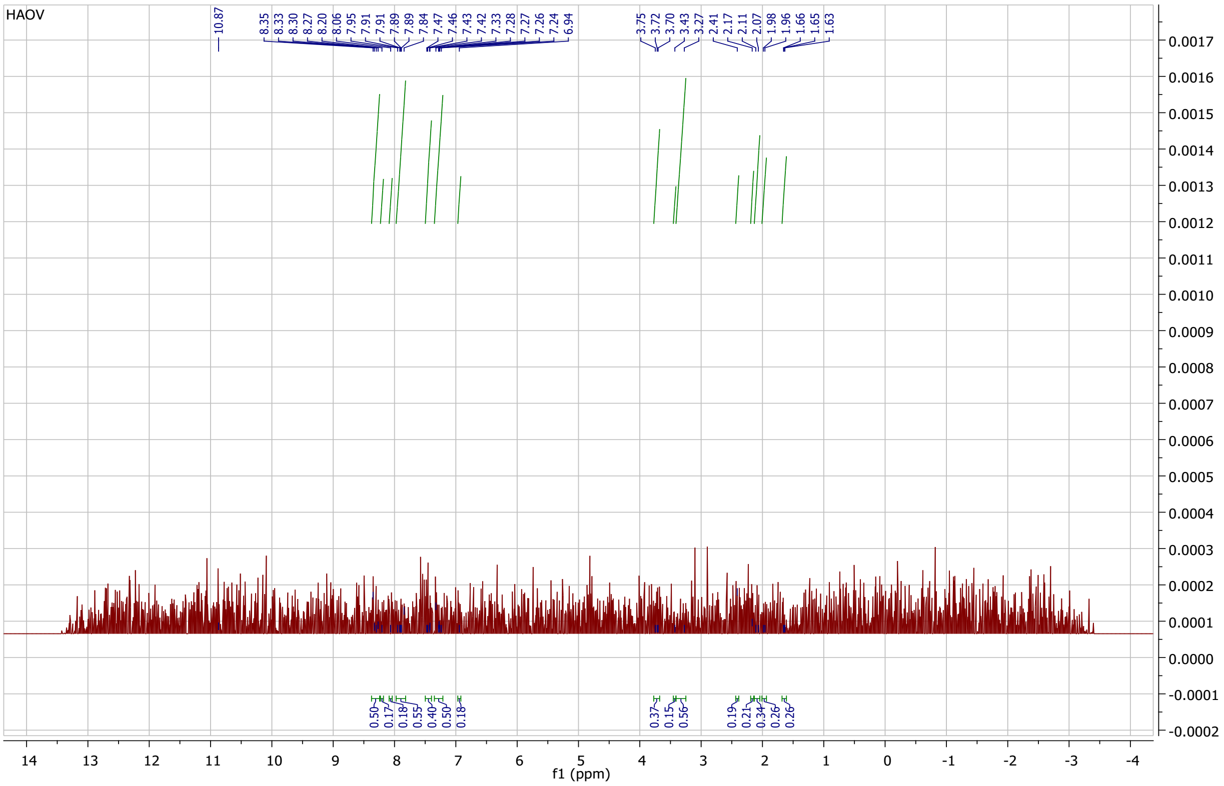

Fig. S29. ^{1}H NMR spectra of **3j**

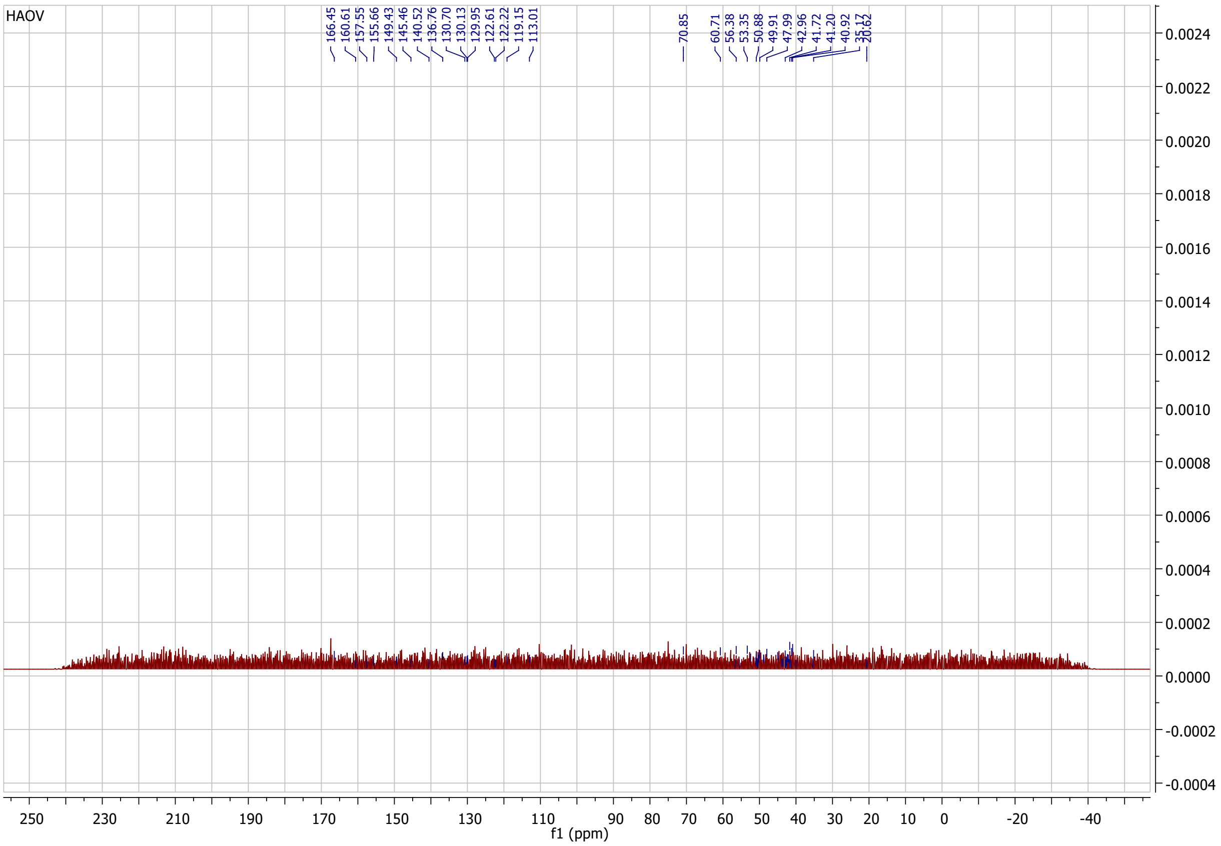

Fig. S30. ^{13}C NMR spectra of **3j**

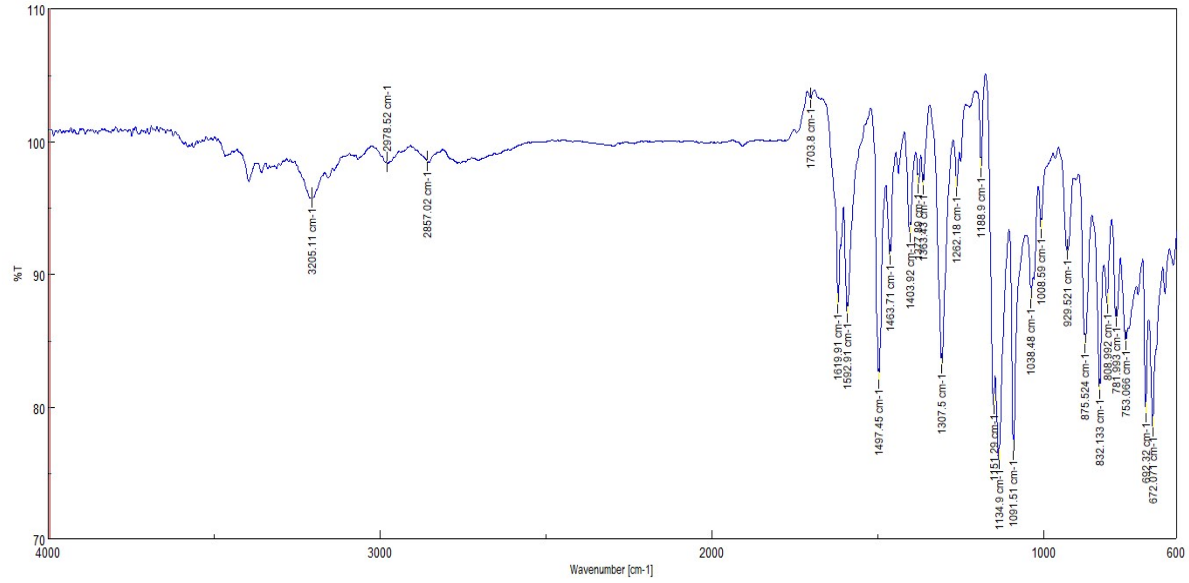

Fig. S31. FT-IR spectra of **4a**

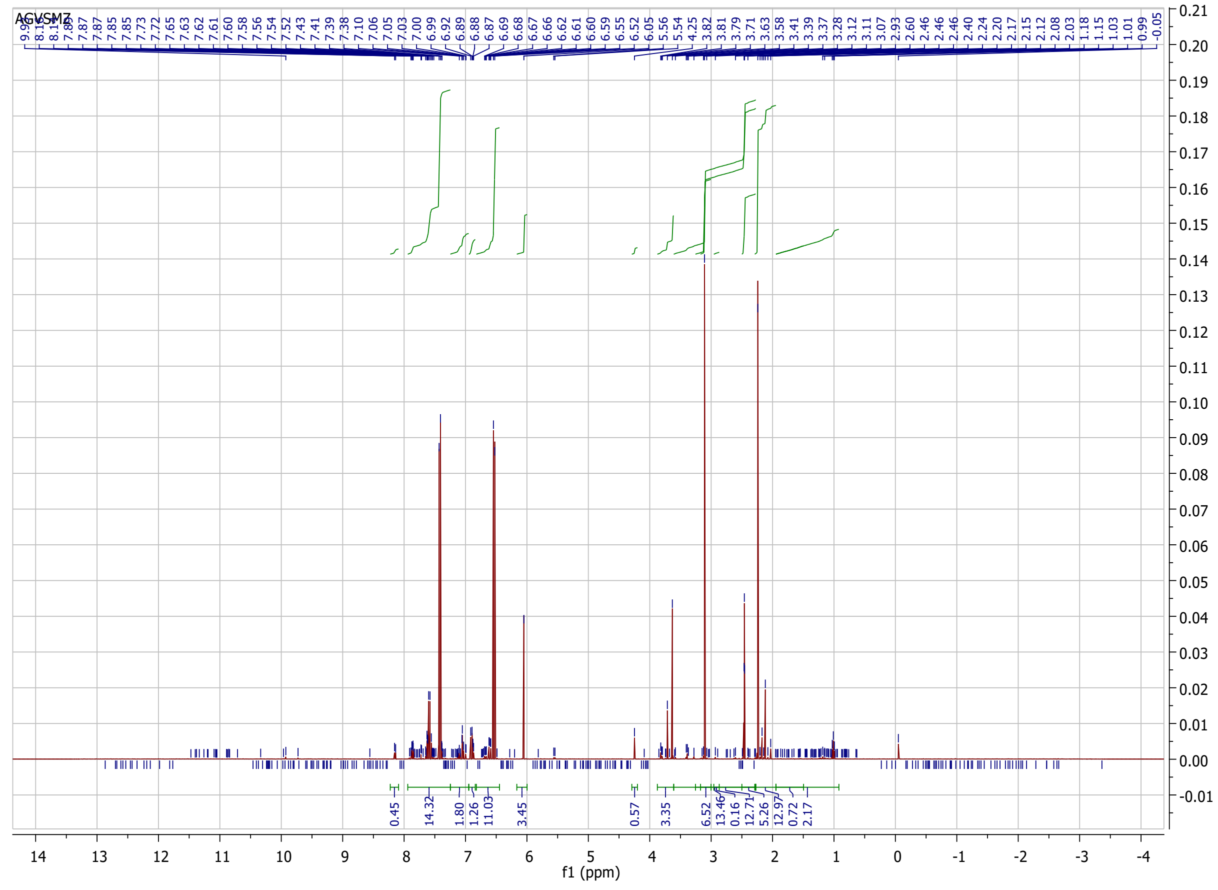

Fig. S32. ^{1}H NMR spectra of **4a**

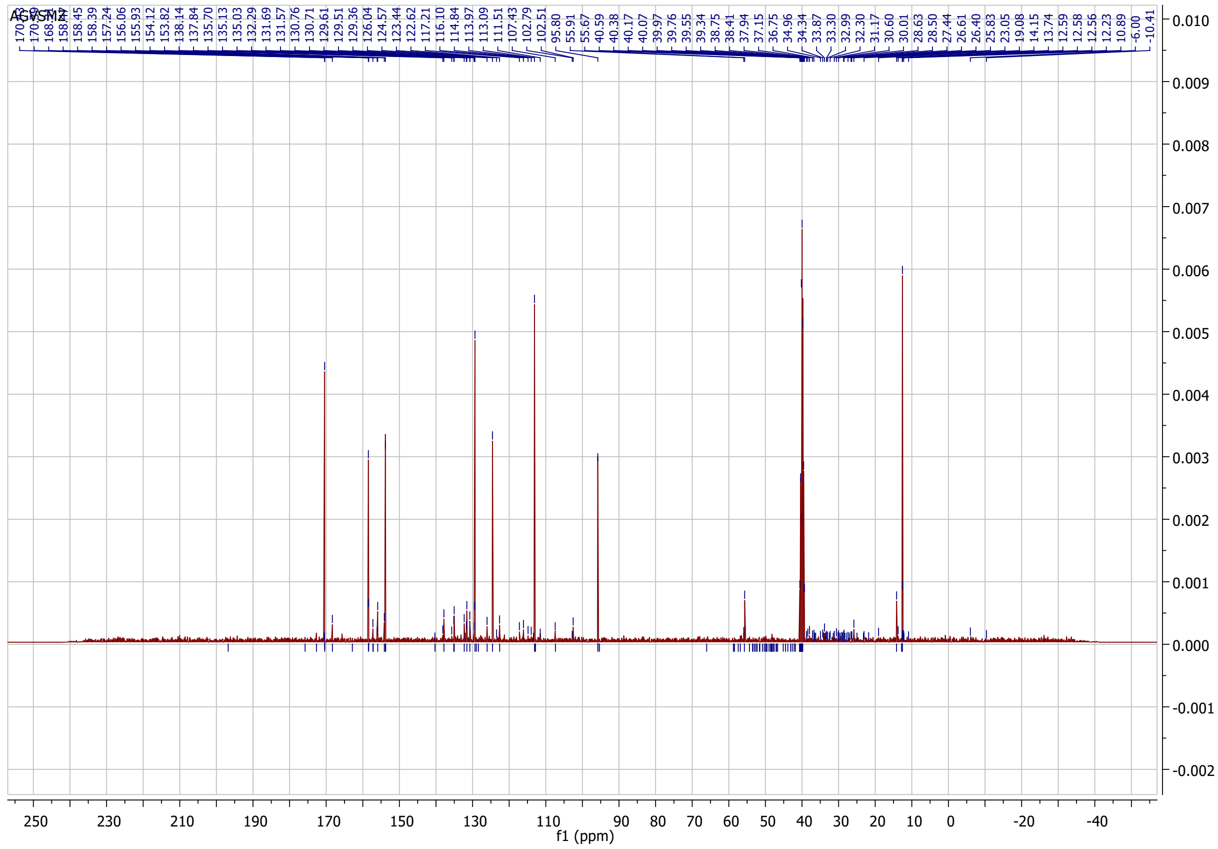

Fig. S33. ^{13}C NMR spectra of **4a**

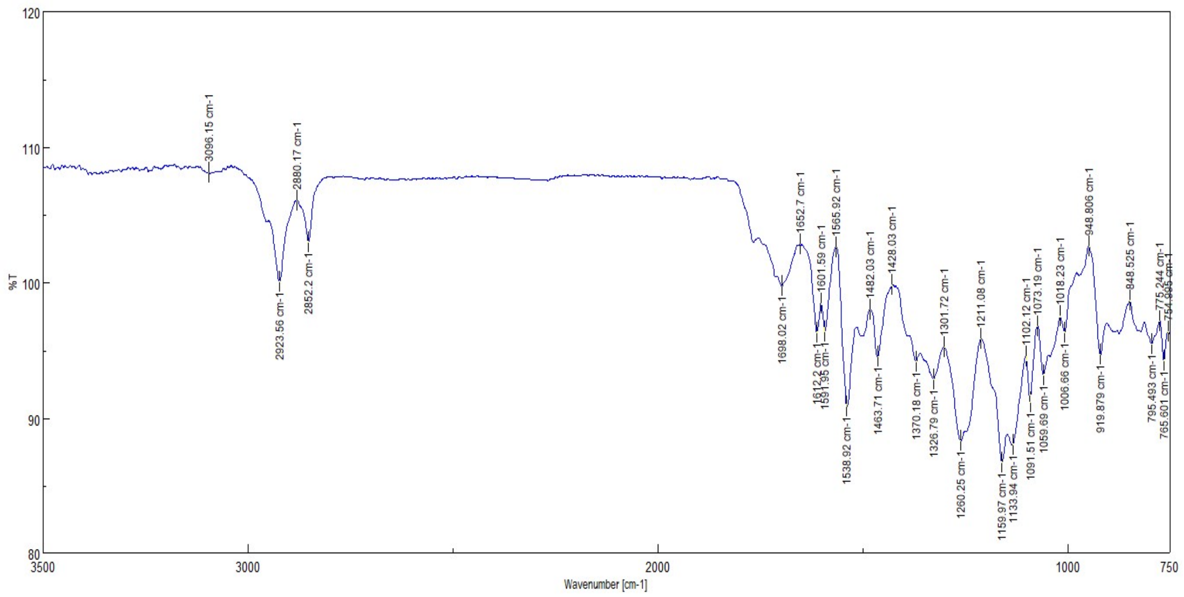

Fig. S34. FT-IR spectra of **4b**

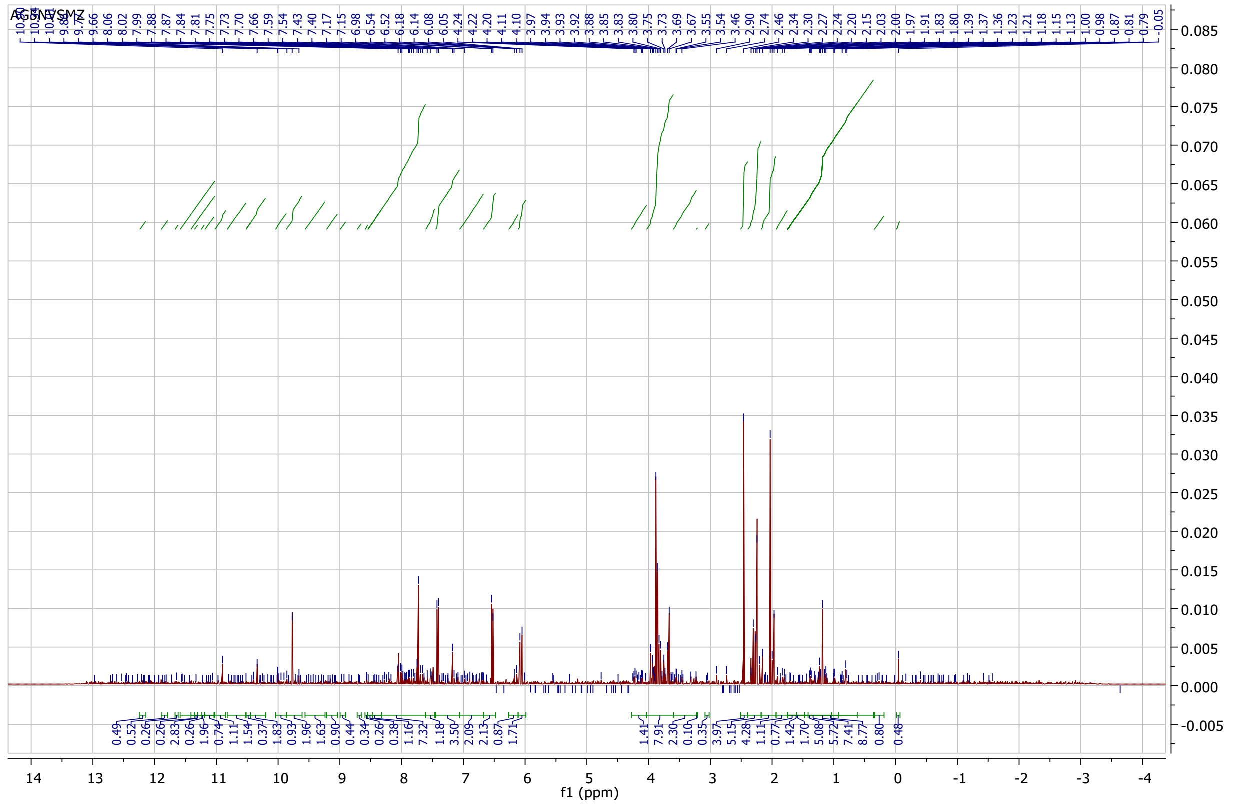

Fig. S35. ^{1}H NMR spectra of **4b**

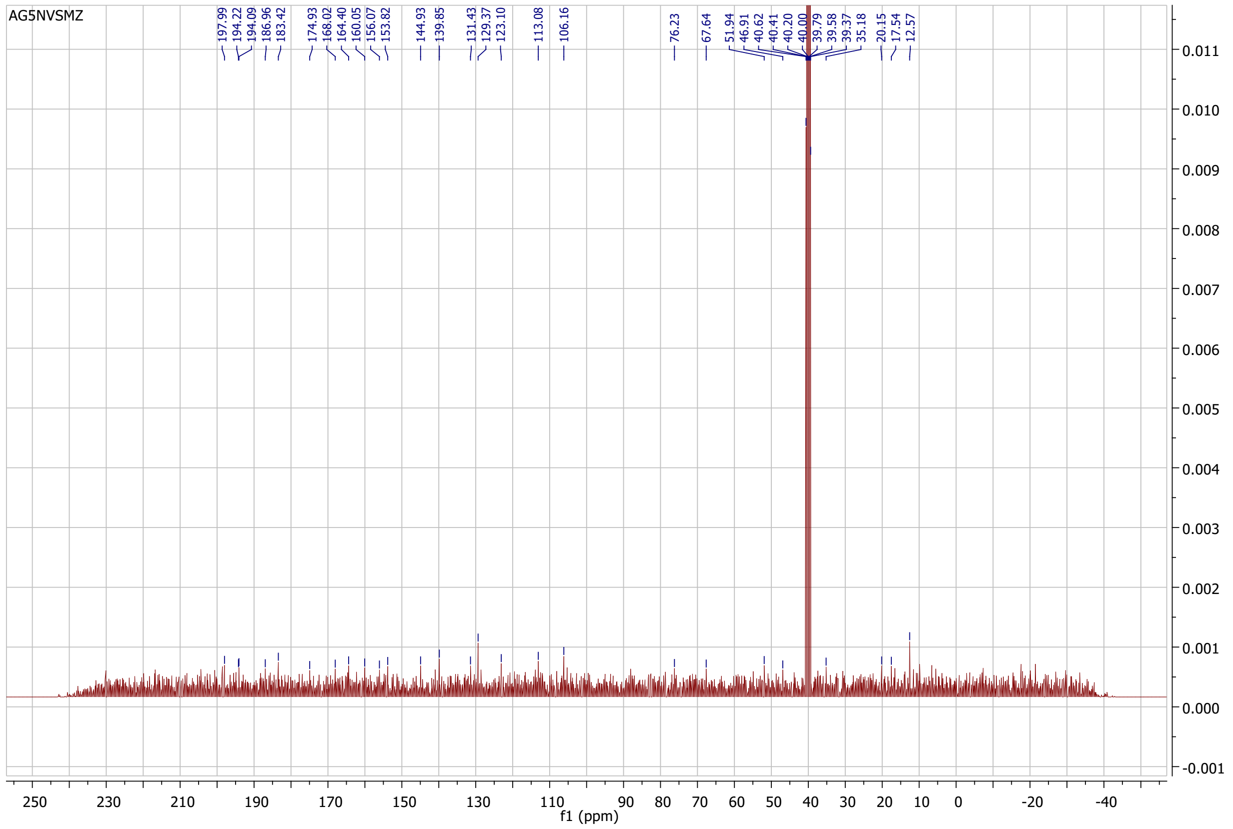

Fig. S36. ^{13}C NMR spectra of **4b**

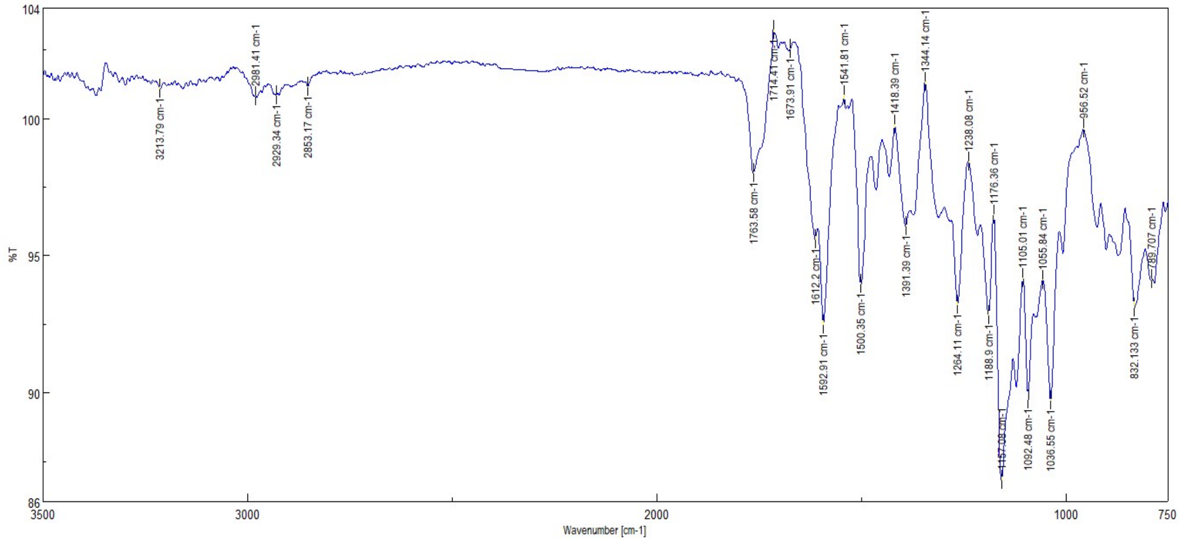

Fig. S37. FT-IR spectra of **4c**

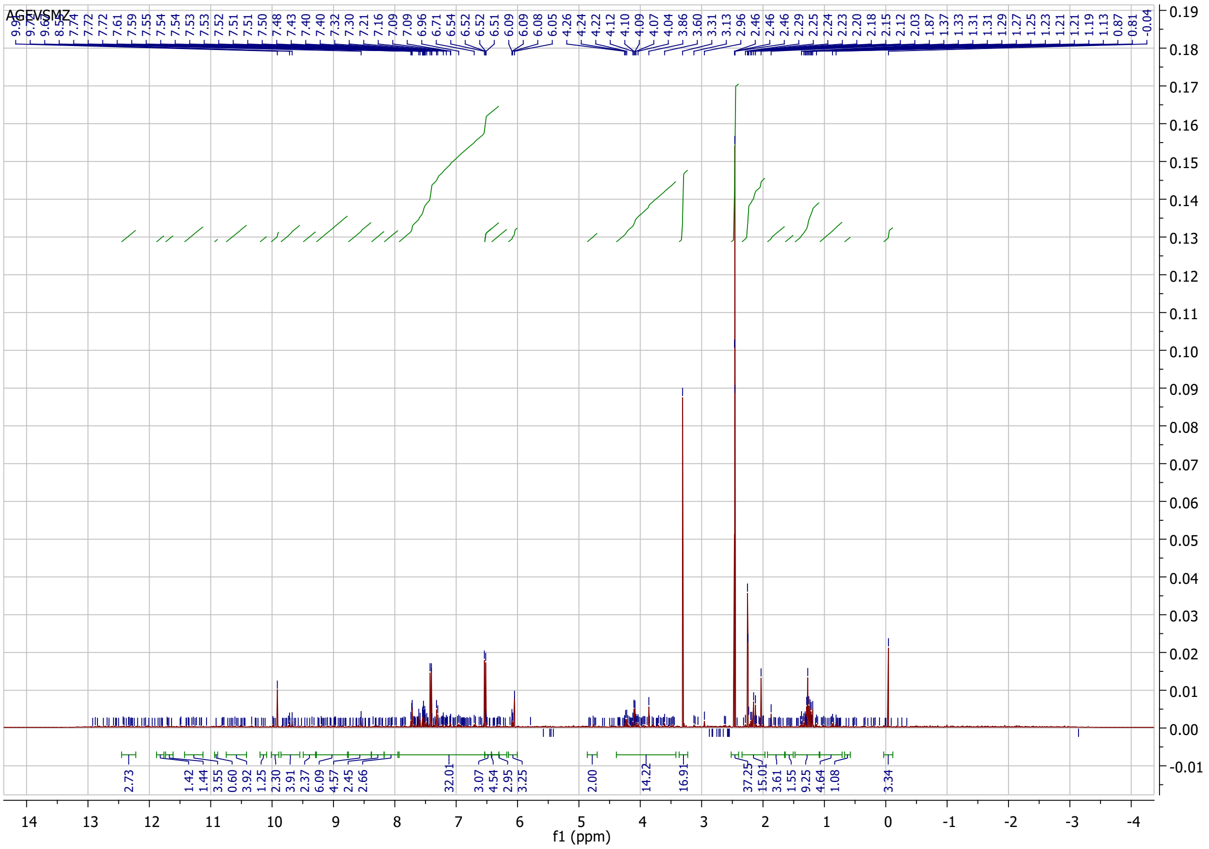

Fig. S38. ^{1}H NMR spectra of **4c**

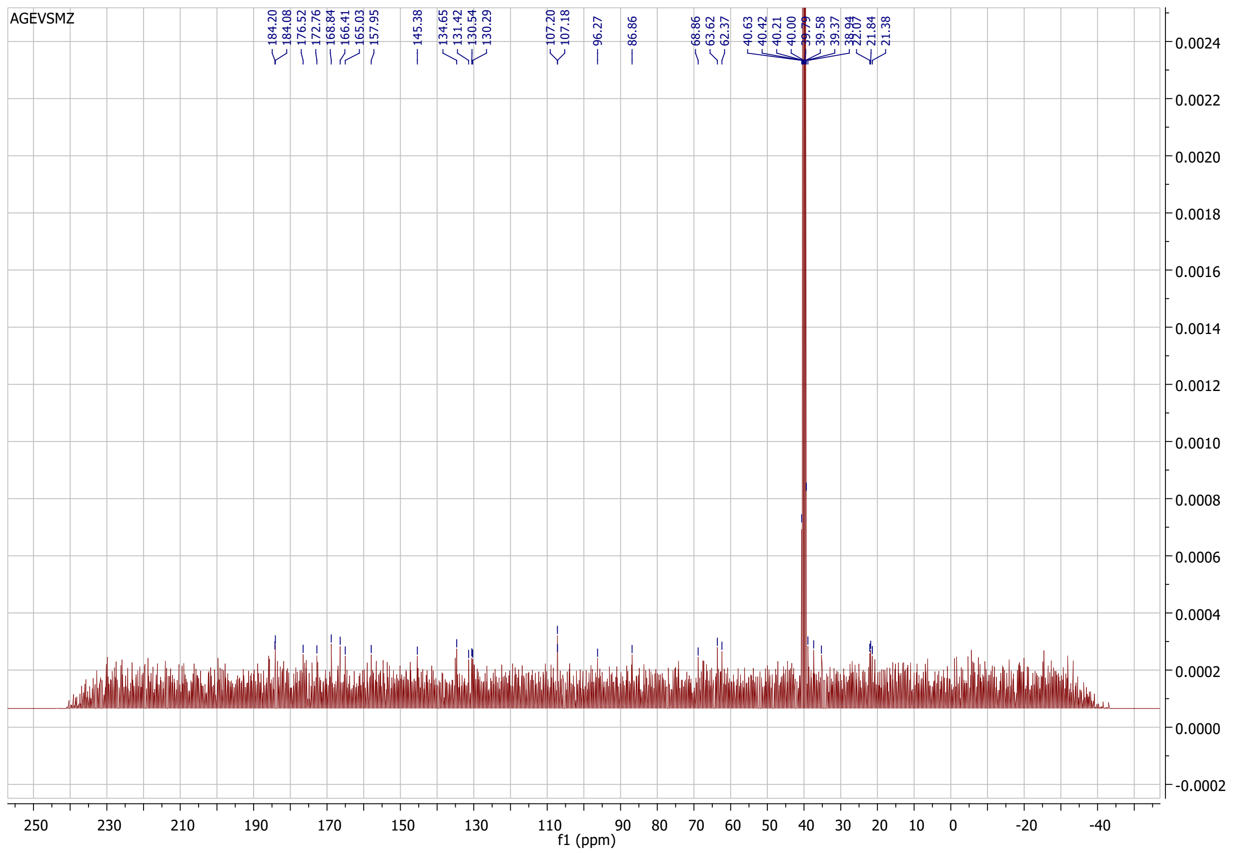

Fig. S39. ^{13}C NMR spectra of **4c**

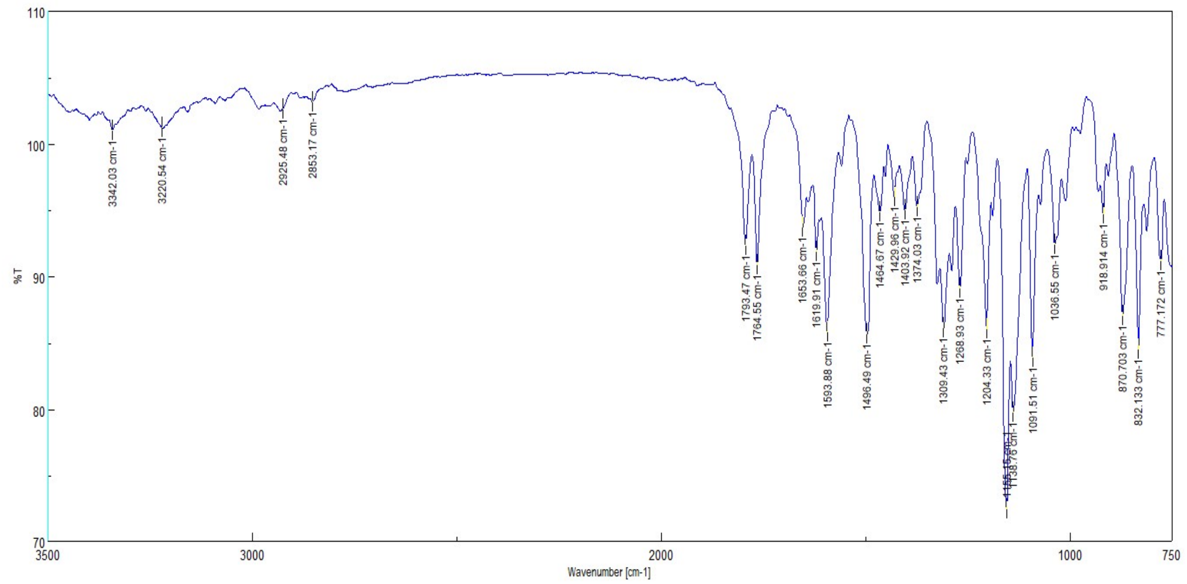

Fig. S40. FT-IR spectra of **4d**

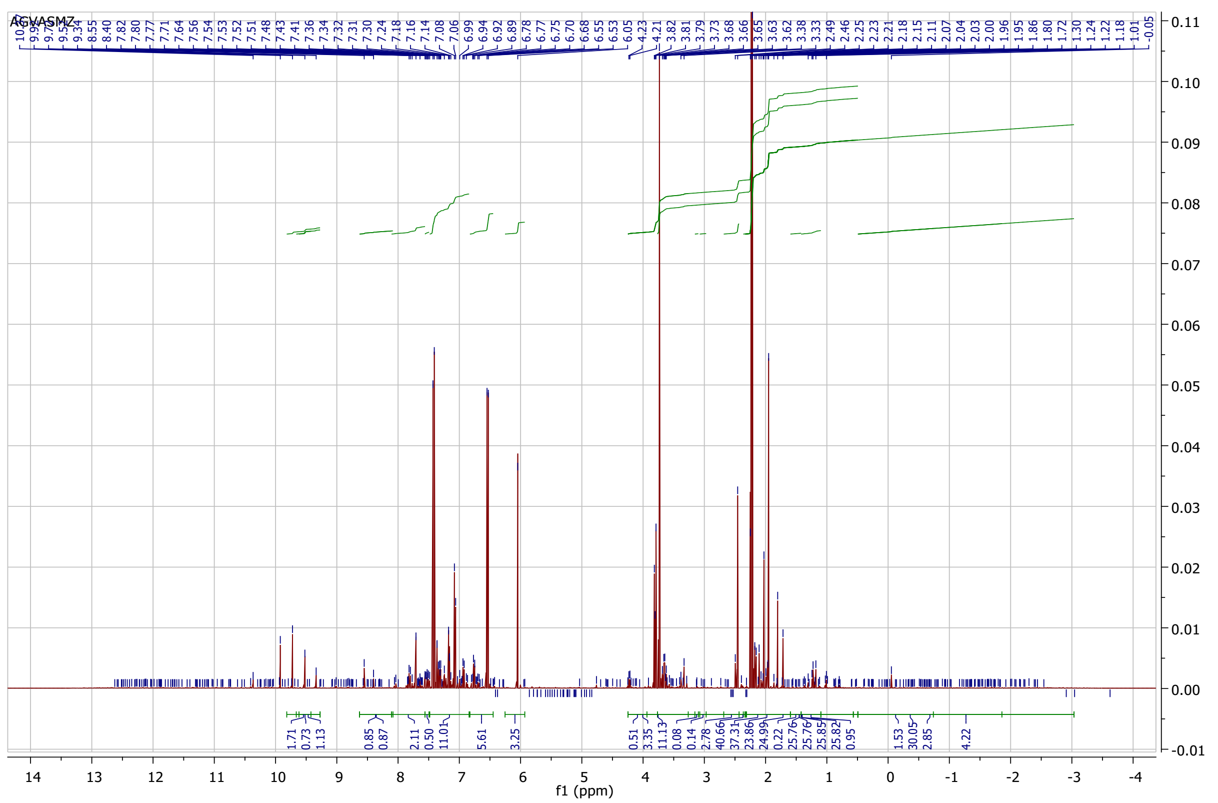

Fig. S41. ^{1}H NMR spectra of **4d**

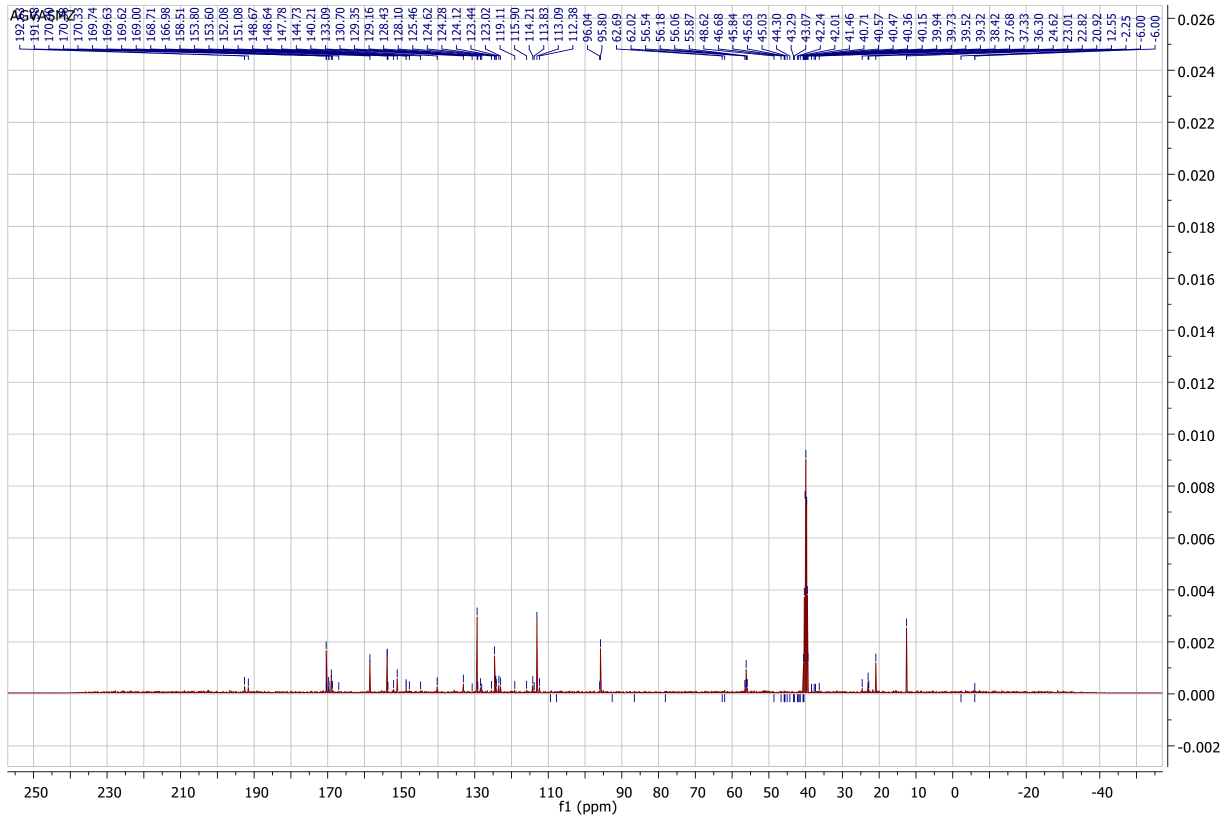

Fig. S42. ^{13}C NMR spectra of **4d**

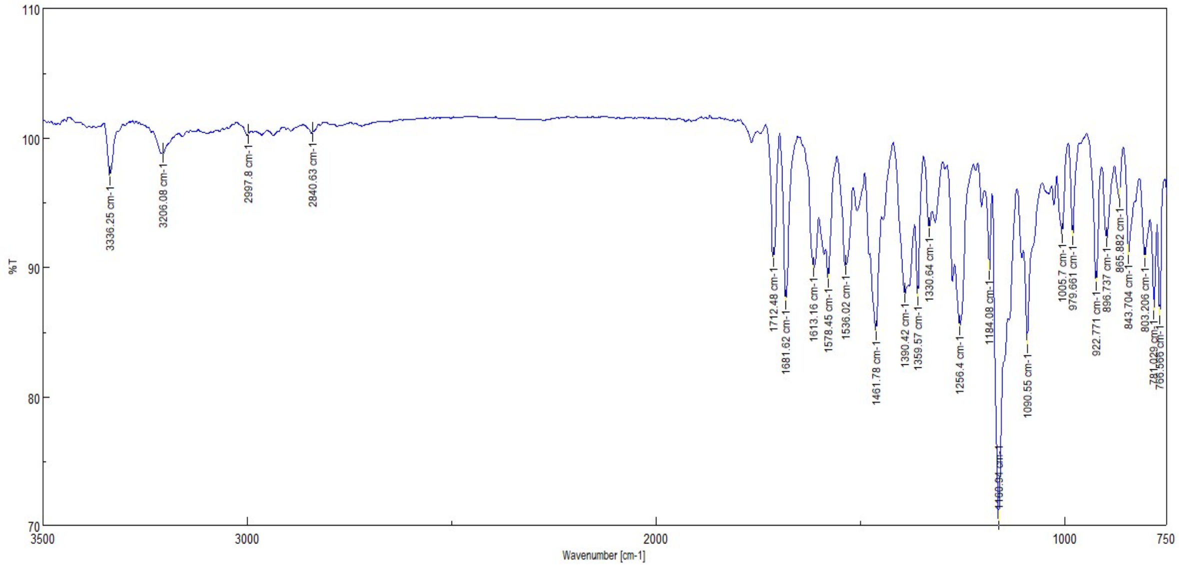

Fig. S43. FT-IR spectra of **4e**

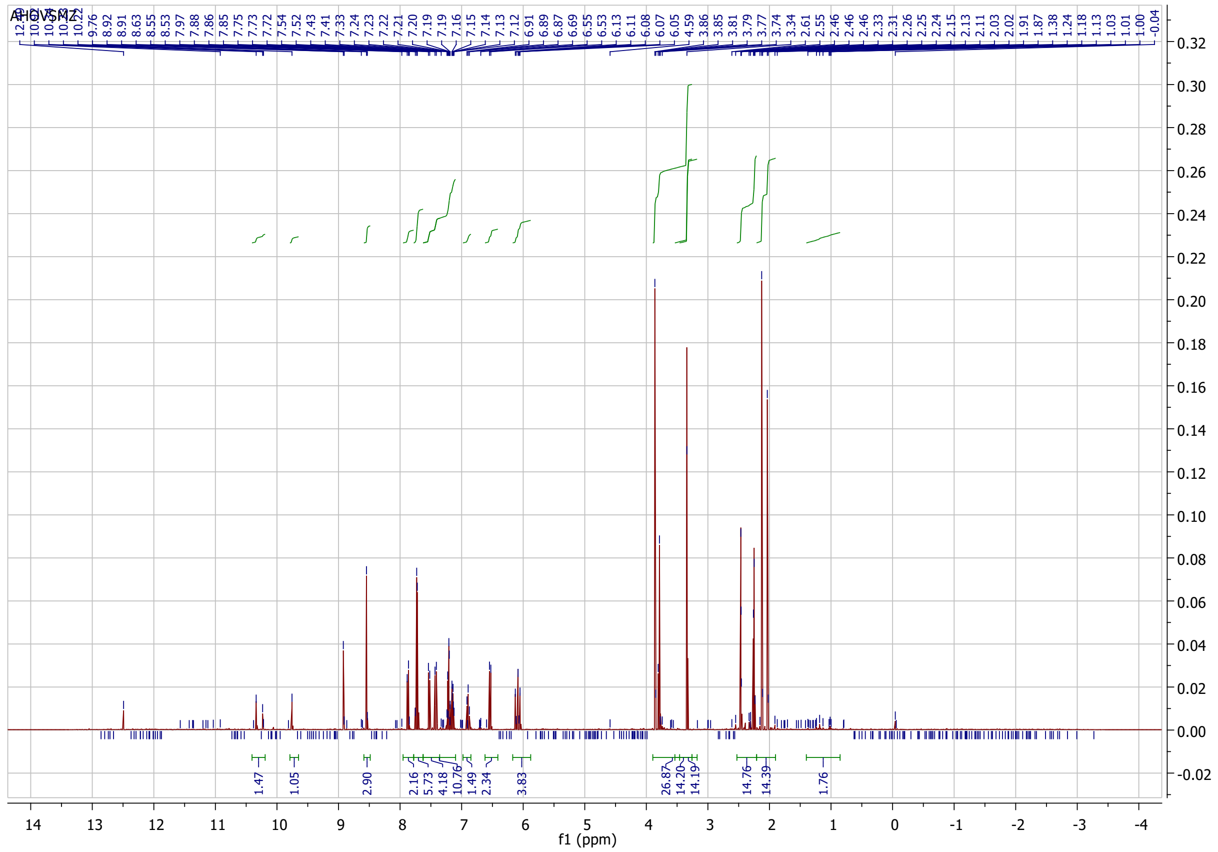

Fig. S44. ^{1}H NMR spectra of **4e**

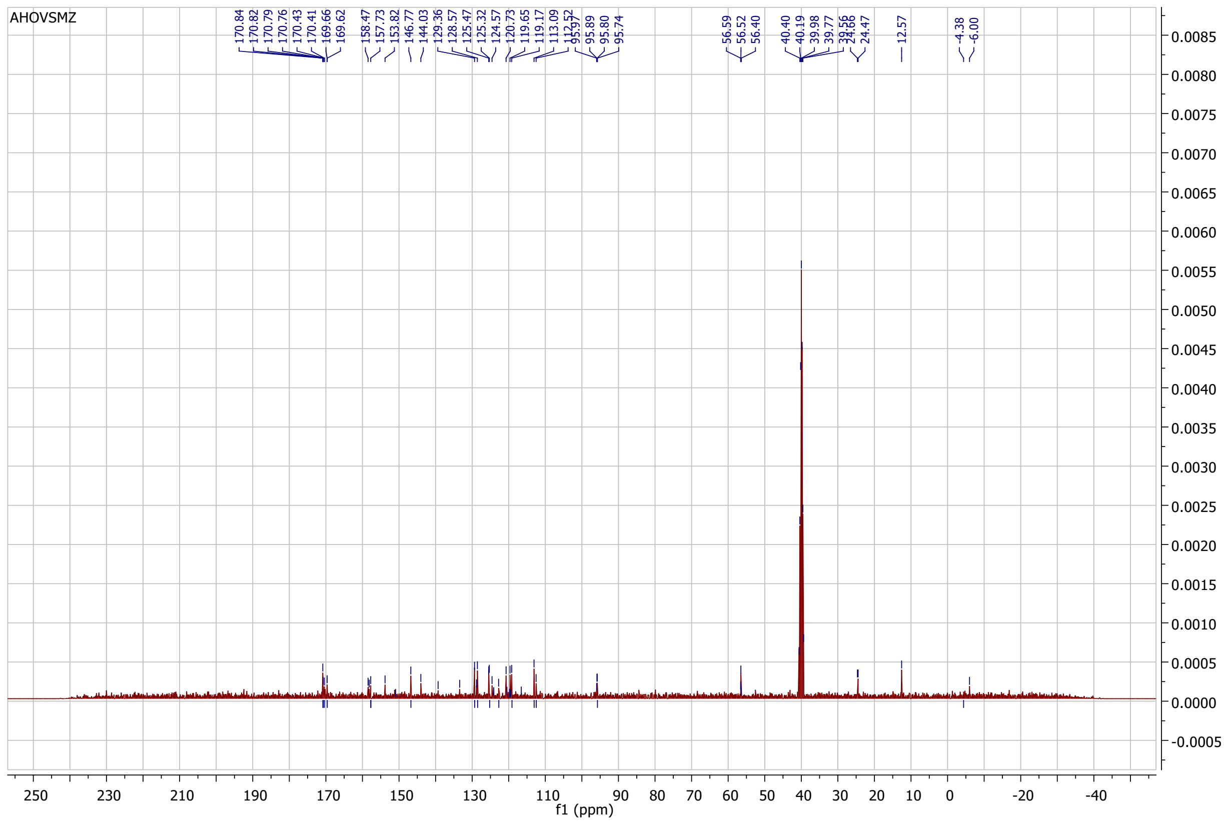

Fig. S45. ^{13}C NMR spectra of **4e**

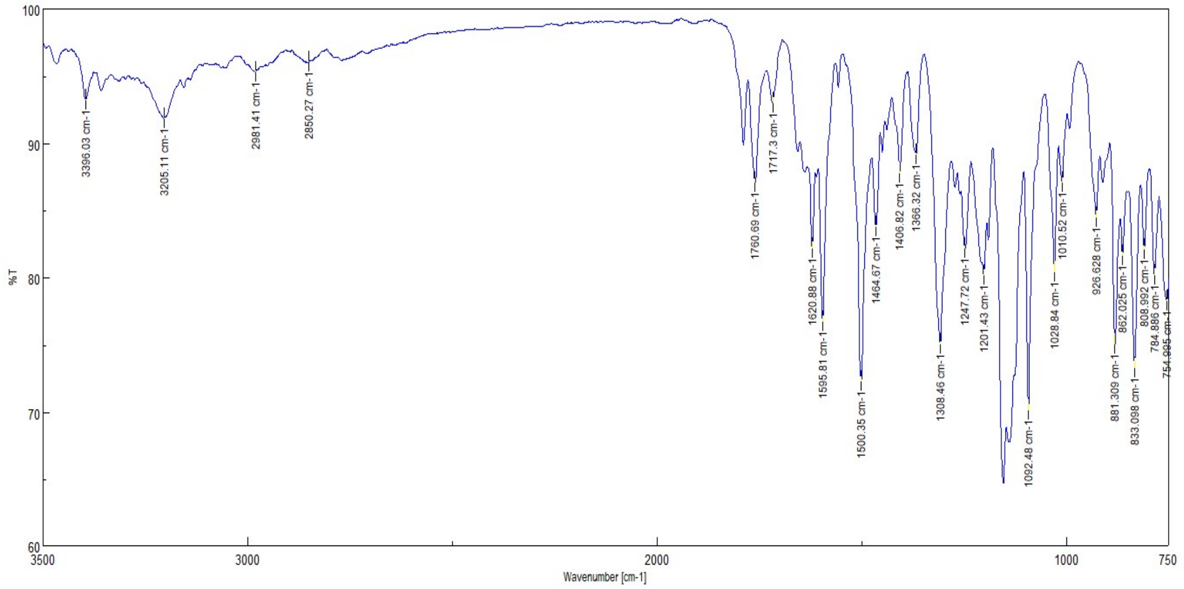

Fig. S46. FT-IR spectra of **4f**

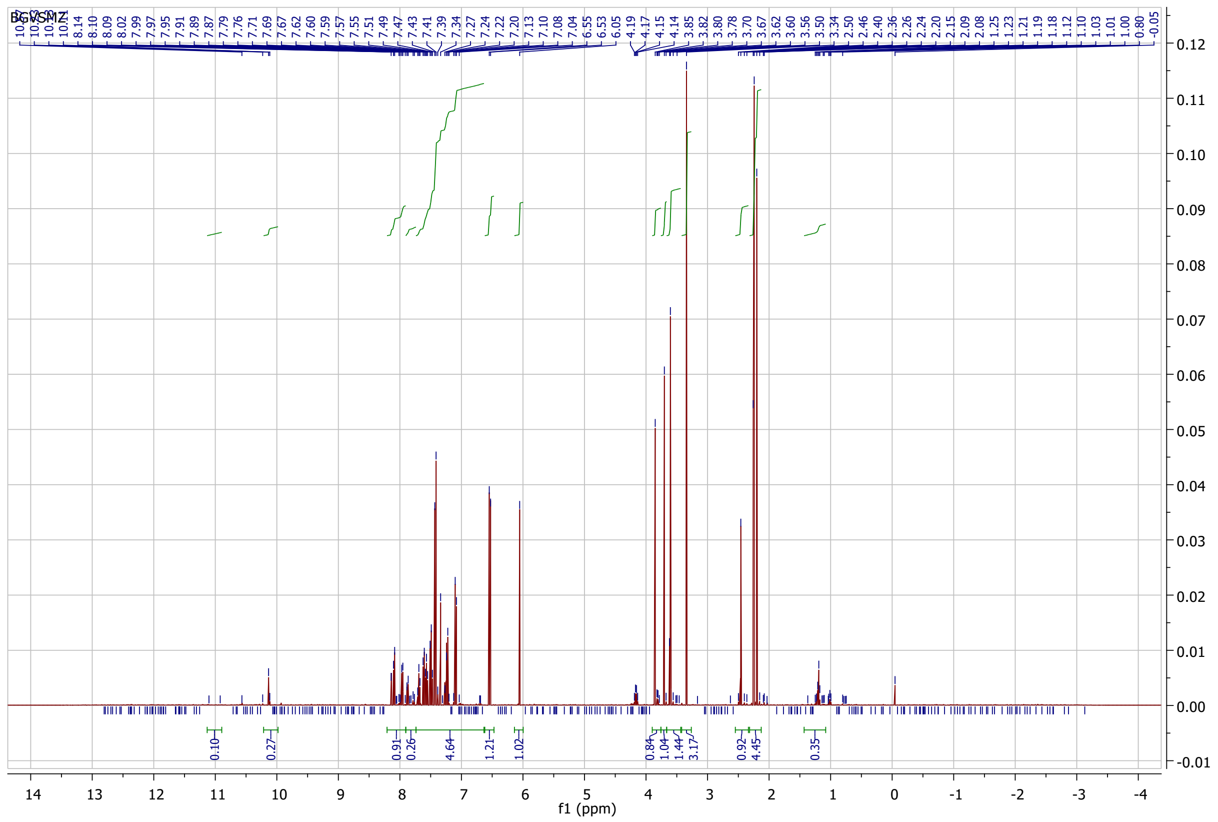

Fig. S47. ^{1}H NMR spectra of **4f**

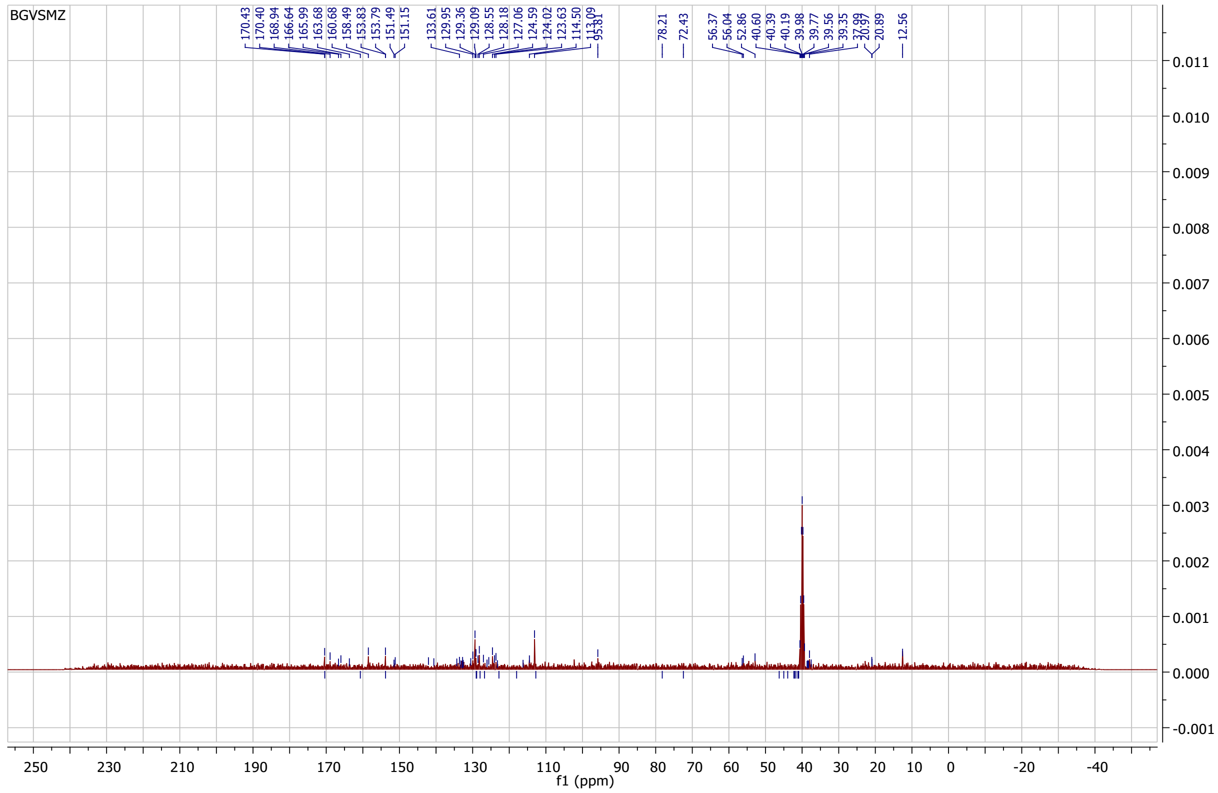

Fig. S48. ^{13}C NMR spectra of **4f**

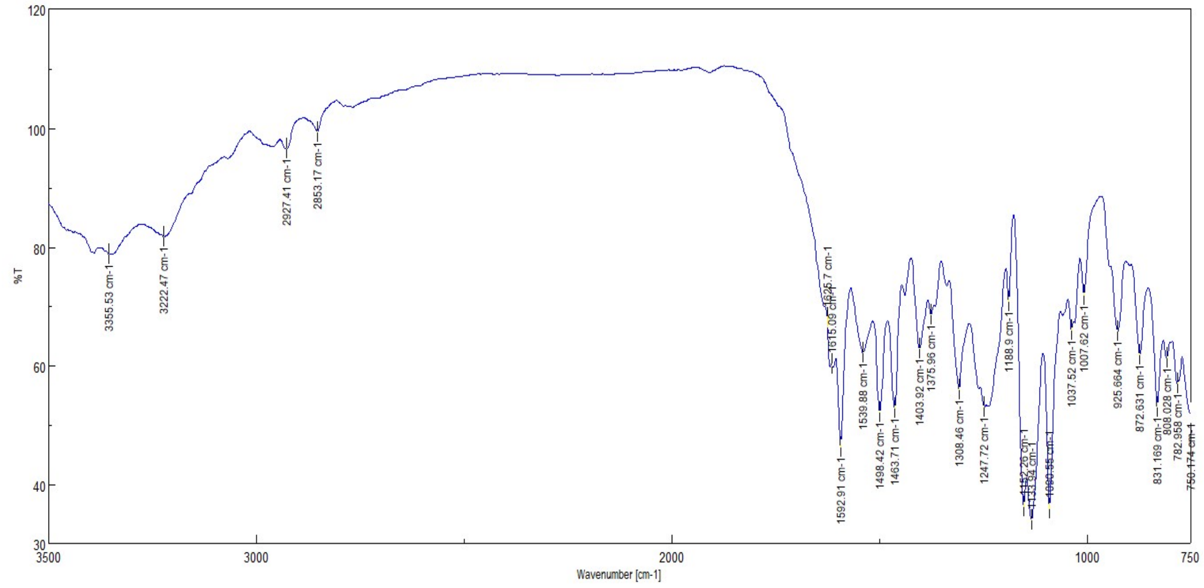

Fig. S49. FT-IR spectra of **4g**

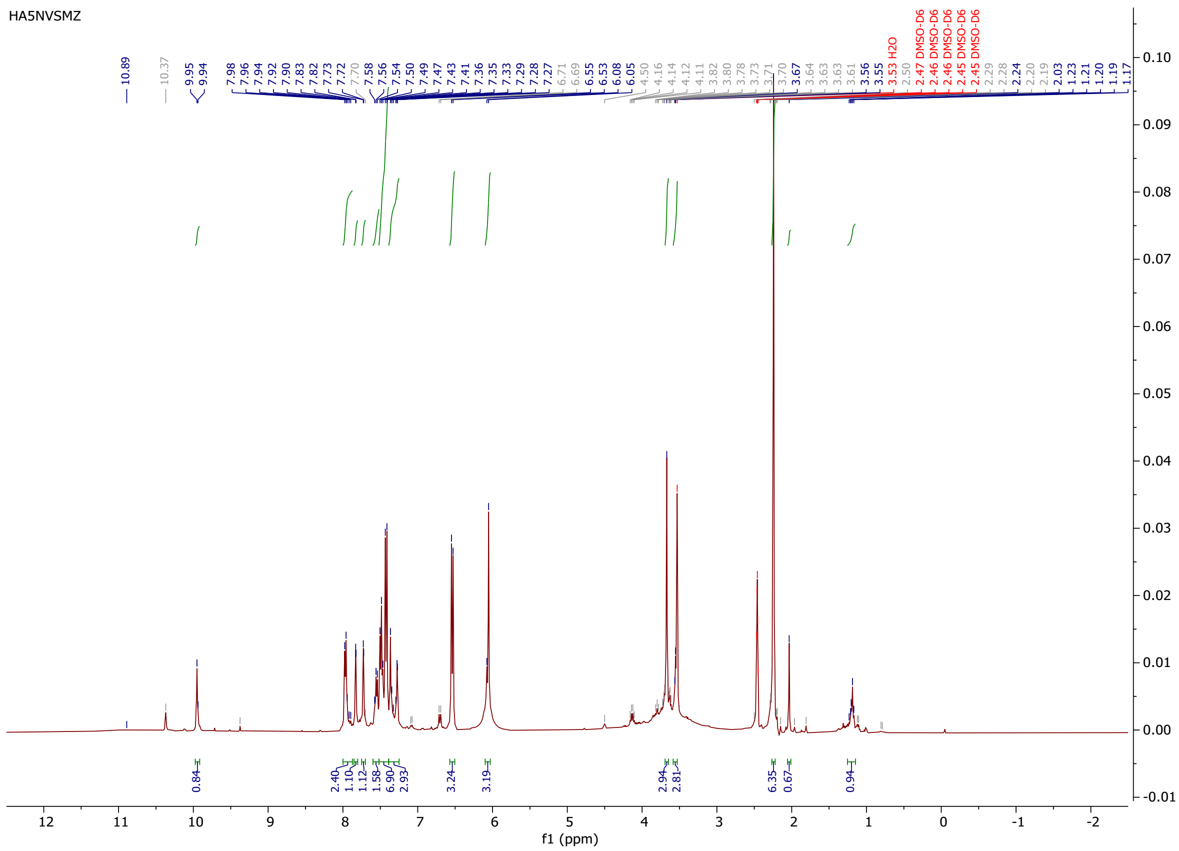

Fig. S50. ^{1}H NMR spectra of **4g**

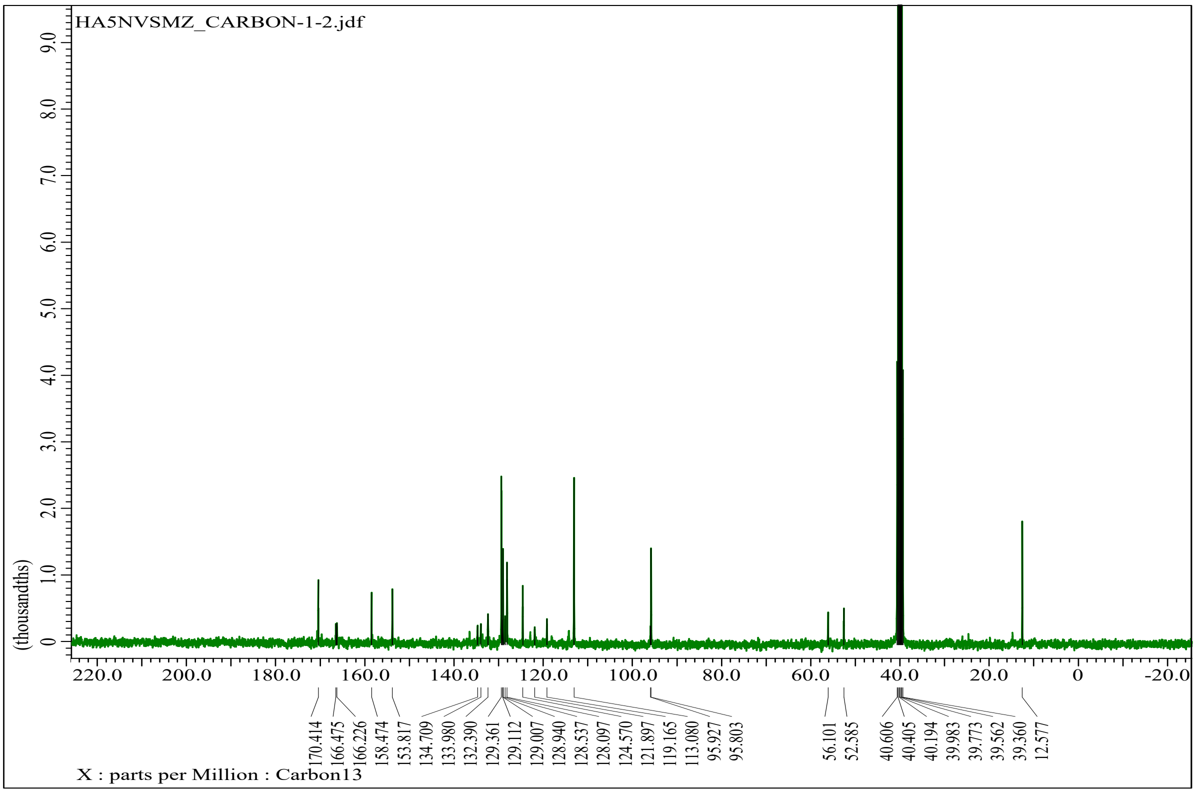

Fig. S51. ^{13}C NMR spectra of **4g**

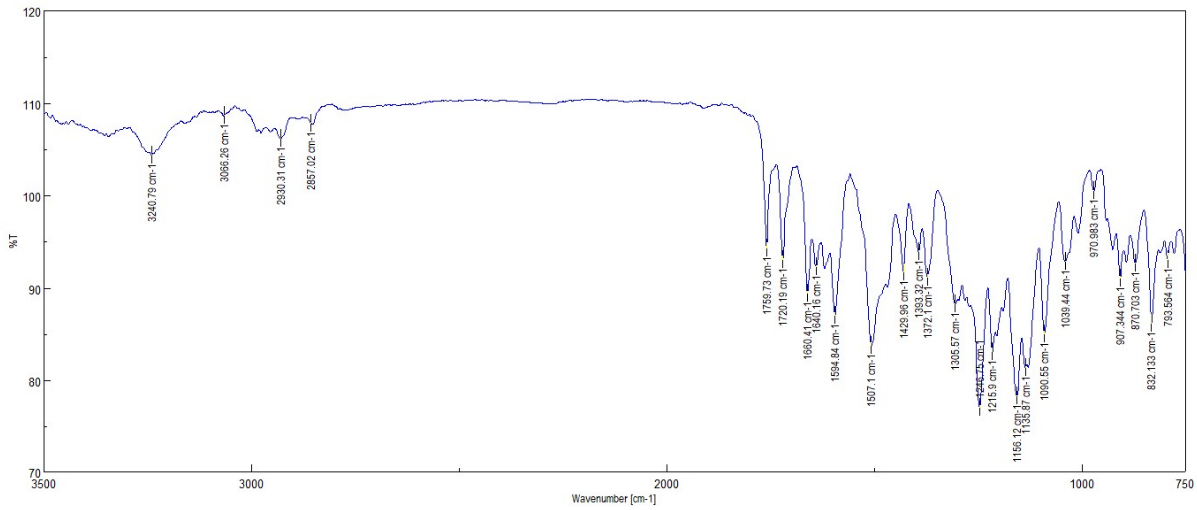

Fig. S52. FT-IR spectra of **4h**

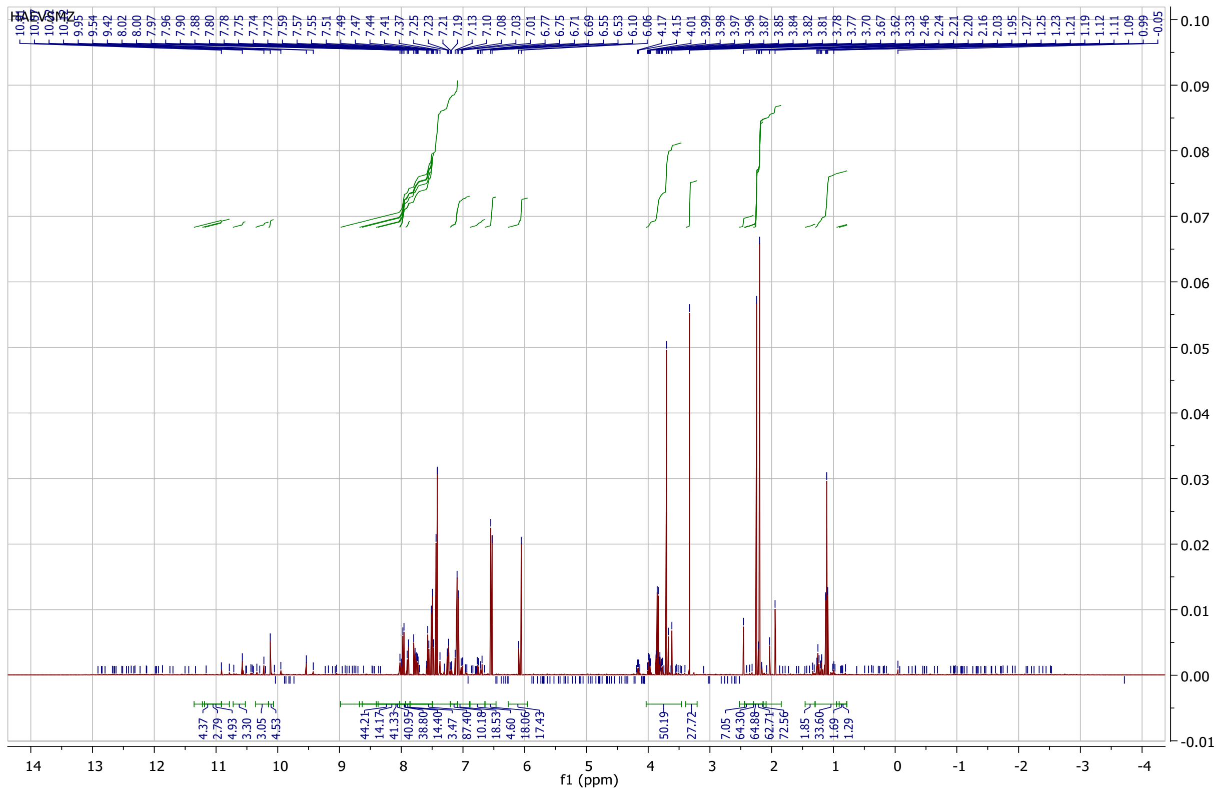

Fig. S53. ^{1}H NMR spectra of **4h**

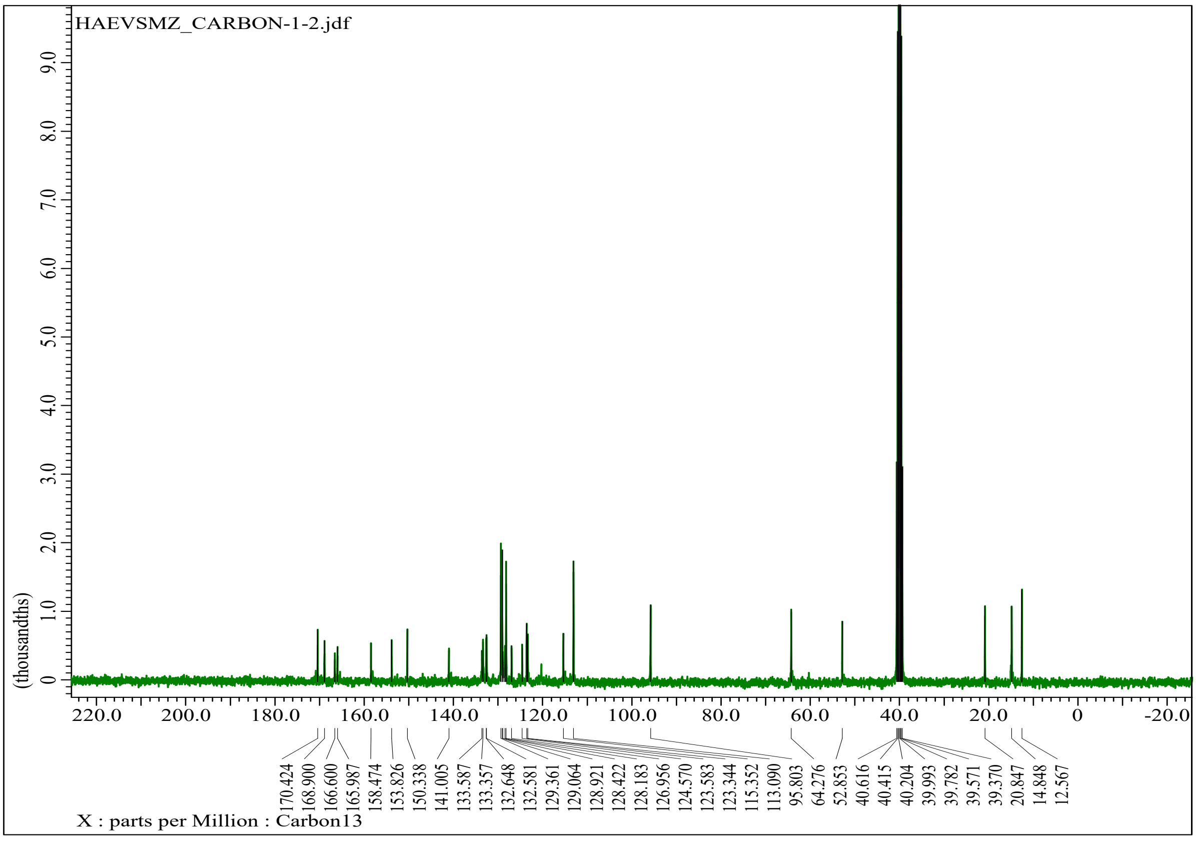

Fig. S54. ^{13}C NMR spectra of **4h**

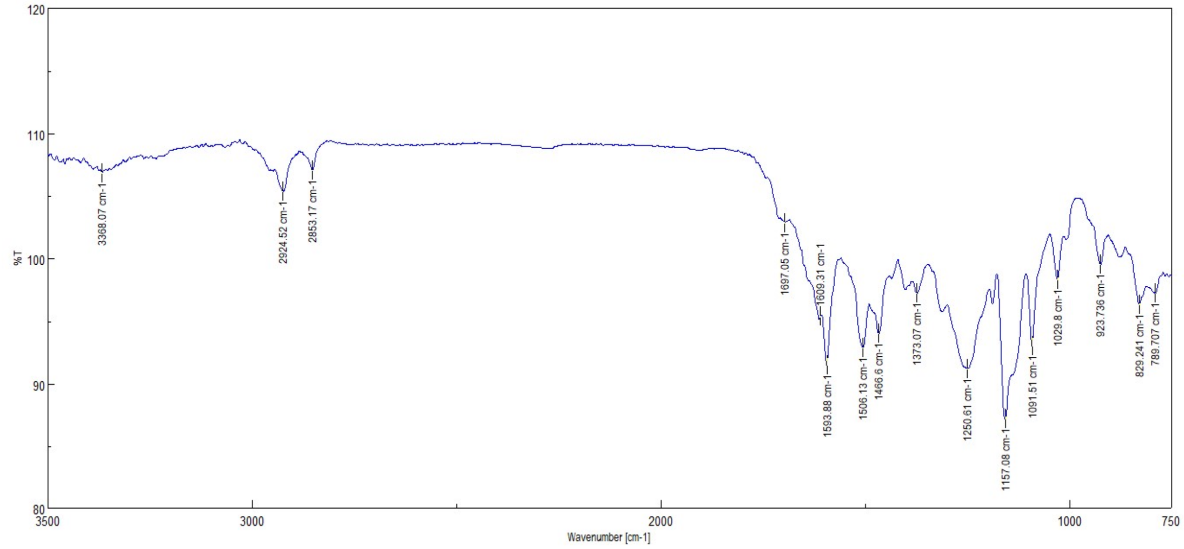

Fig. S55. FT-IR spectra of **4i**

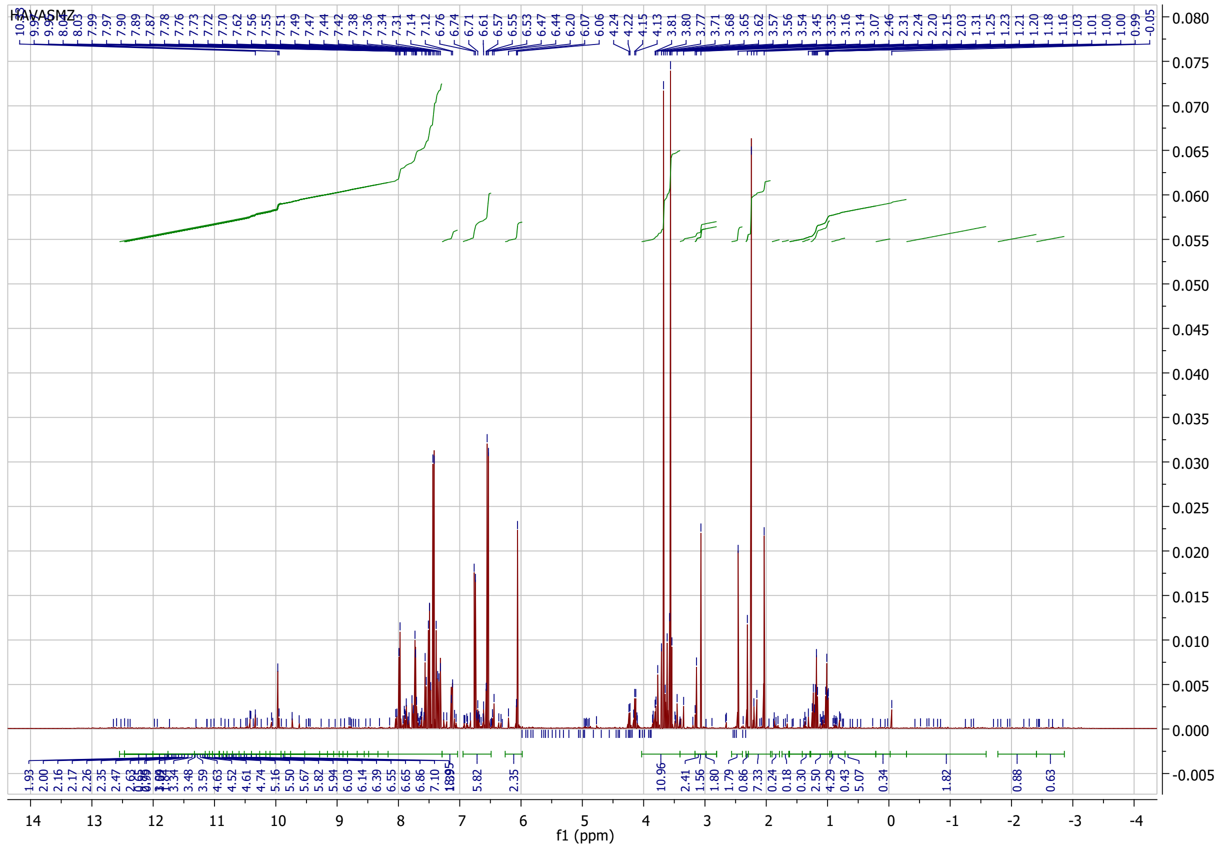

Fig. S56. ^{1}H NMR spectra of **4i**

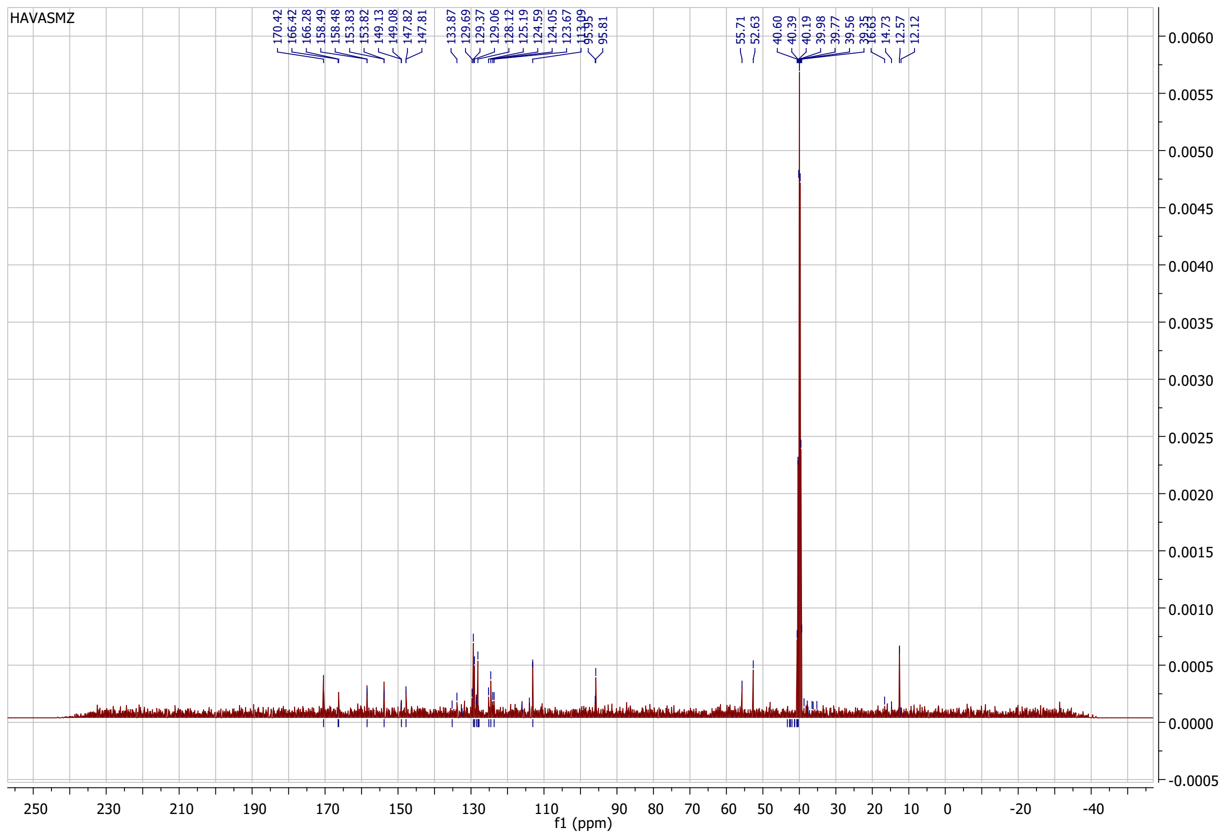

Fig. S57. ^{13}C NMR spectra of **4i**

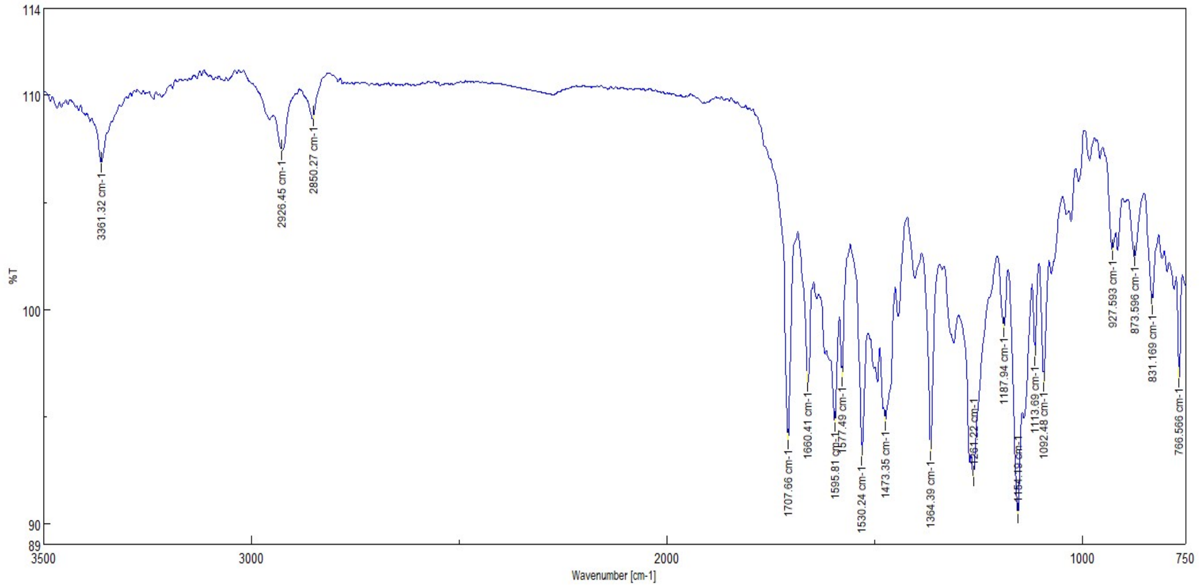

Fig. S58. FT-IR spectra of **4j**

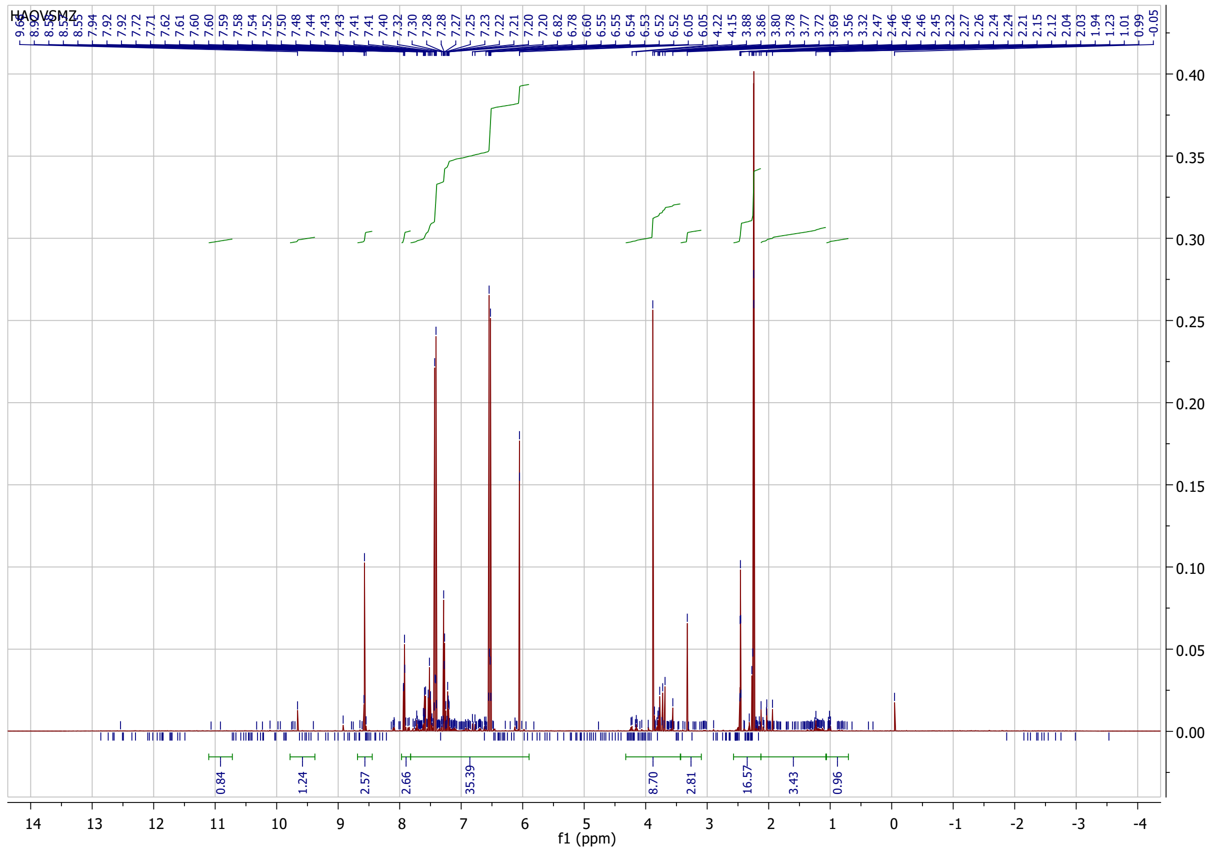

Fig. S59. ^{1}H NMR spectra of **4j**

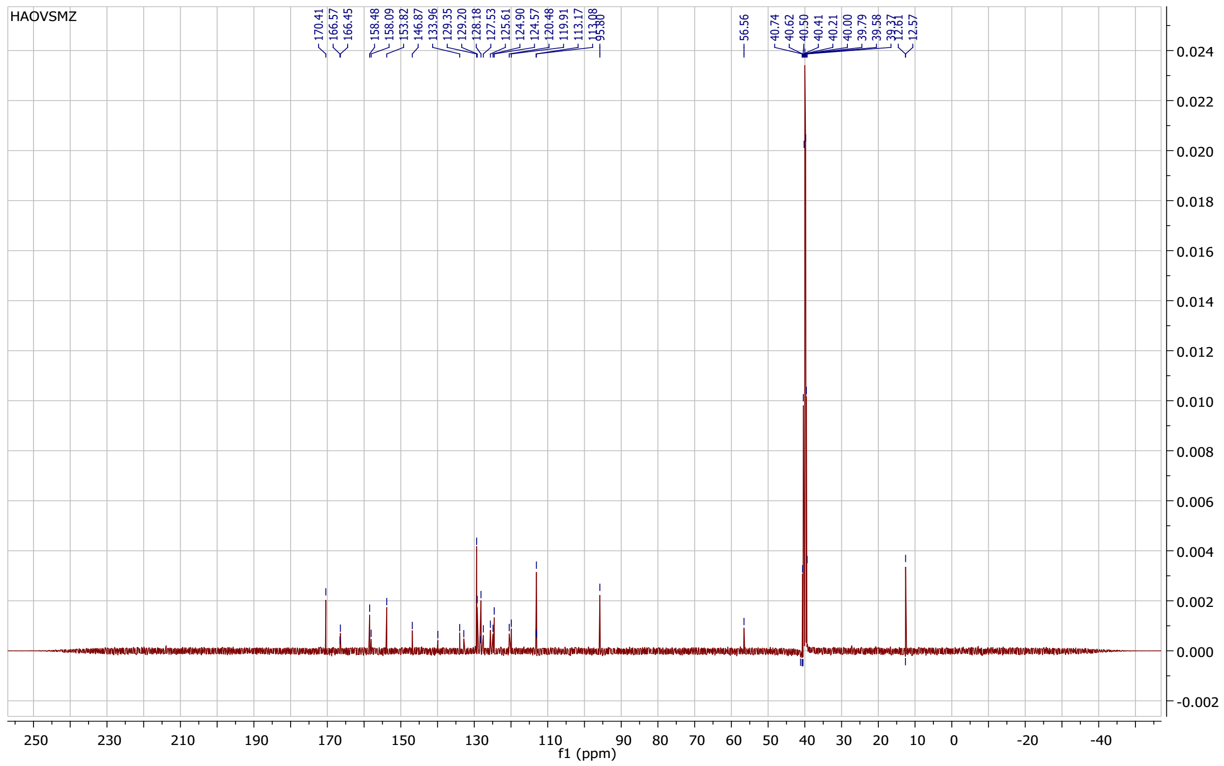

Fig. S60. ^{13}C NMR spectra of **4j**

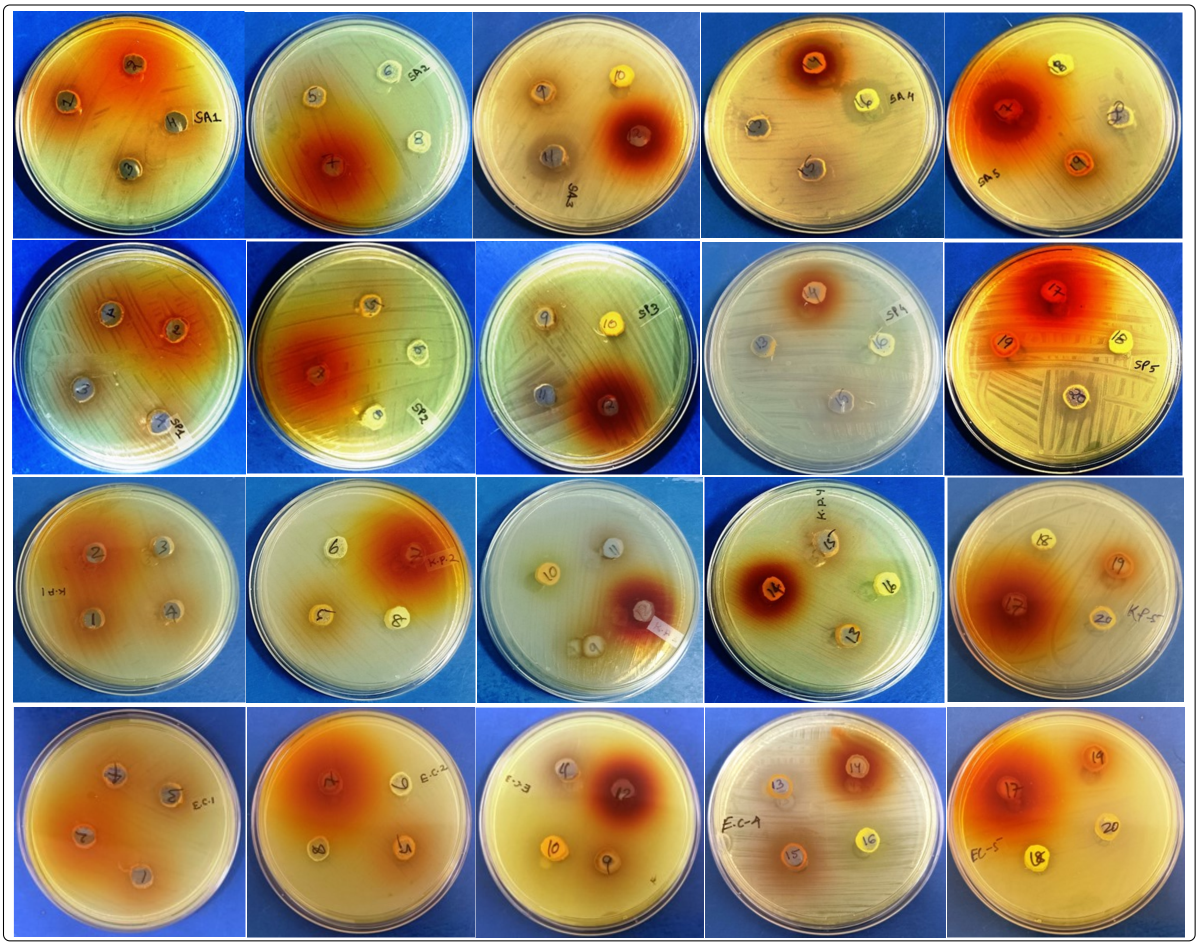

Fig. S61. Zone of inhibition plates of all compounds against four distinct MDR bacterial pathogens viz. **SA**- *S. aureus*, **SP**- *S. pyogenes*, **KP**- *K. pneumoniae*, **EC**- *E. colli*; Numbering 1-10 denotes series **3a-3j** and numbering 11-20 denotes series **4a-4j**.

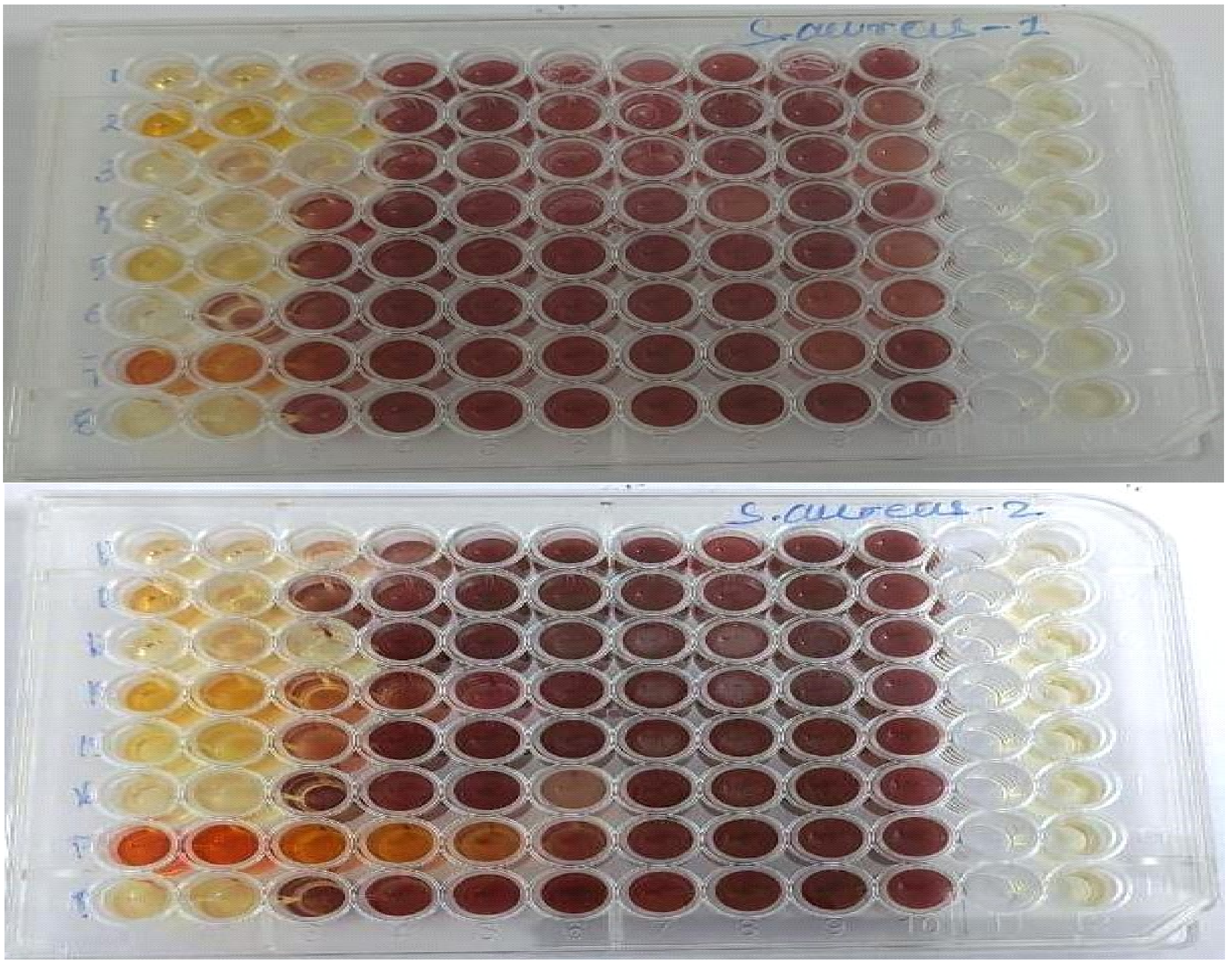

Fig. S62. MIC value of candidates **3a-3h** and **4a-4h** against MDR *S. aureus*.

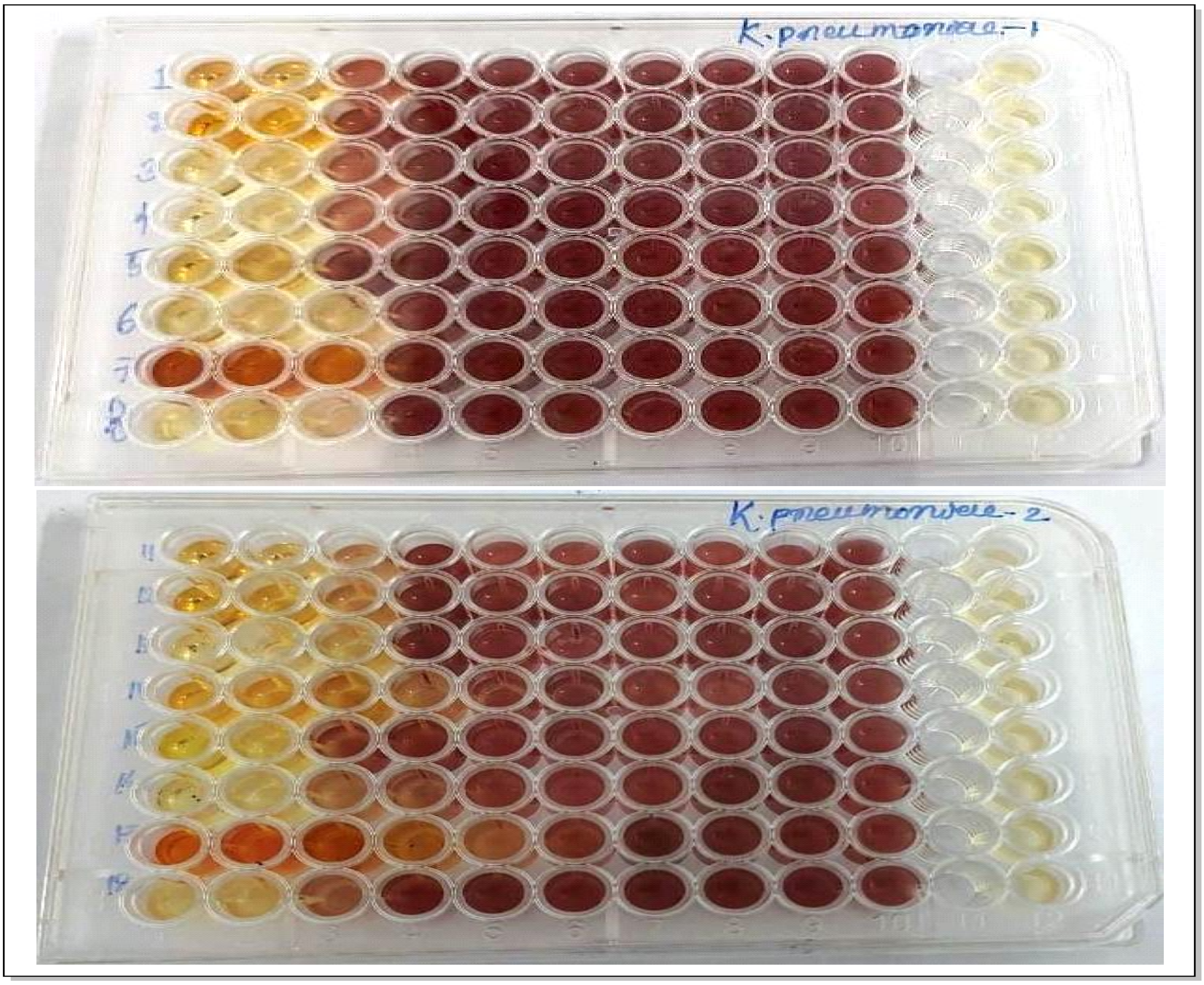

Fig. S63. MIC value of candidates **3a-3h** and **4a-4h** against MDR *K. pneumoniae*.

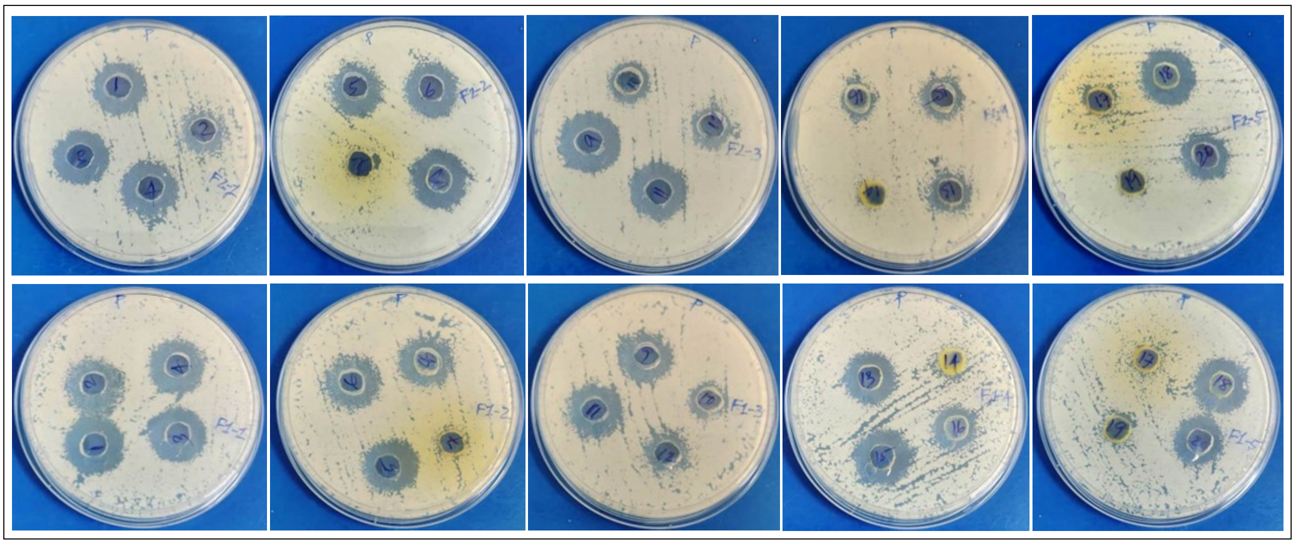

Fig. S64. Zone of inhibition plates of all compounds against two MDR fungal strains viz. **F1-** *C. tropicalis* and **F2-** *T. rubrum*; Numbering 1-10 denotes series **3a-3j** and numbering 11-20 denotes series **4a-4j**.

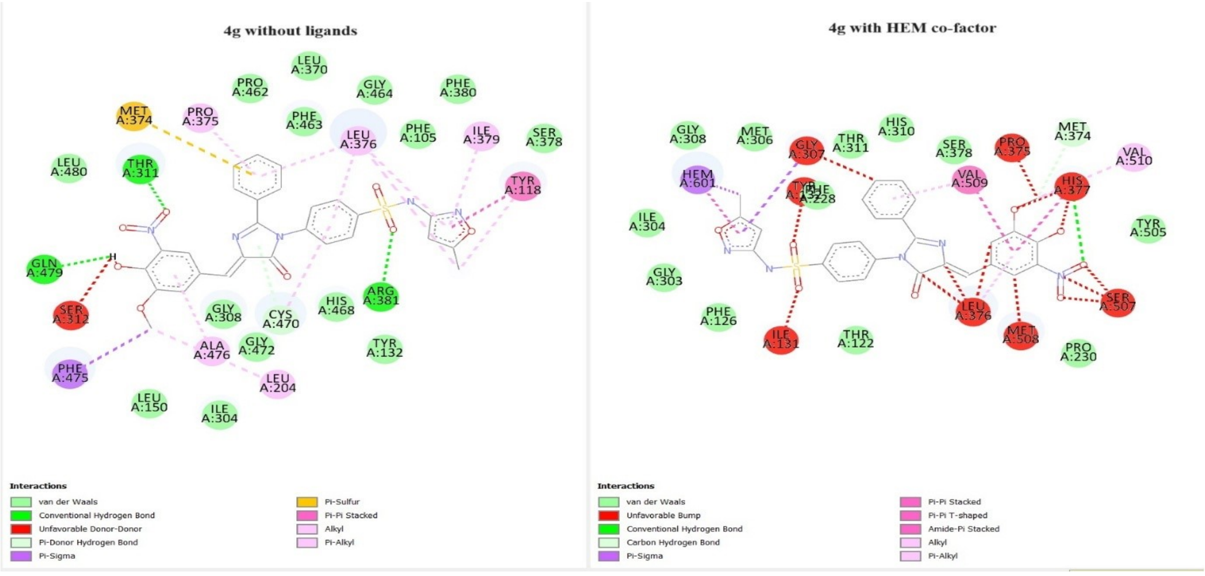

Fig. S65. Comparison between co-crystallized ligand bearing protein and protein structure having HEM co-factor of compound **4g** as a validation protocol.

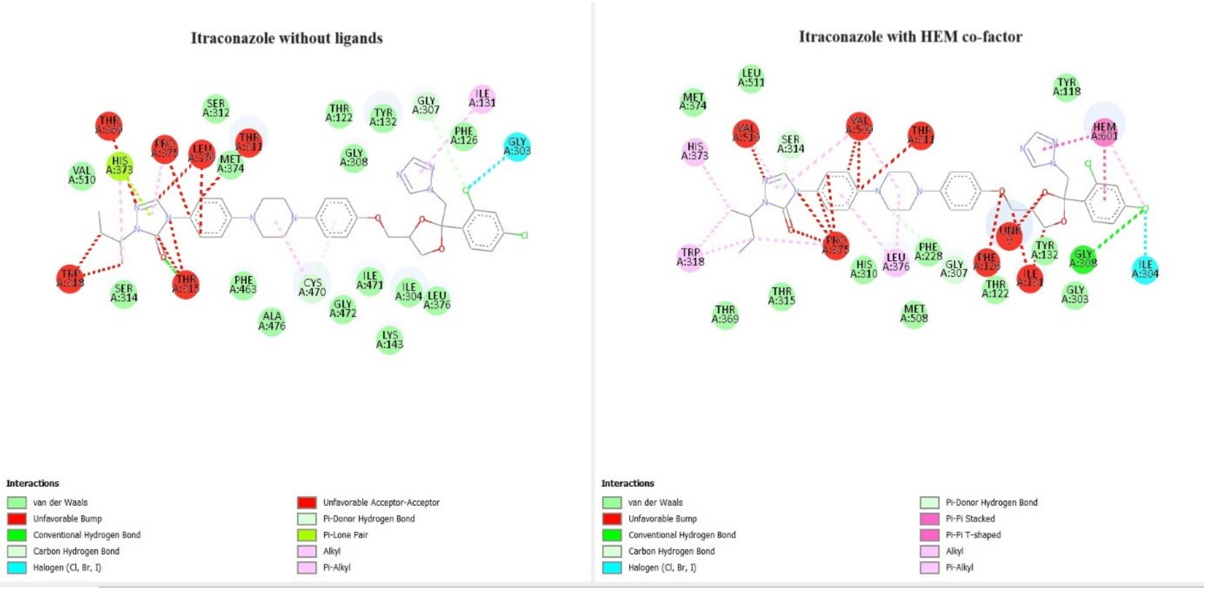

Fig. S66. Comparison between co-crystallized ligand bearing protein and protein structure having HEM co-factor of compound **Itraconazole** as a validation protocol.
